# Generalized Sparse Additive Models

**Published:** 2022

**Authors:** Asad Haris, Noah Simon, Ali Shojaie

**Affiliations:** Department of Earth, Ocean and Atmospheric Sciences, University of British Columbia, 2020 – 2207 Main Mall, Vancouver, BC, Canada V6T 1Z4; Department of Biostatistics, University of Washington, Seattle, WA 98195-7232, USA; Department of Biostatistics, University of Washington, Seattle, WA 98195-7232, USA

**Keywords:** Generalized Additive Models, Sparsity, Minimax, High-Dimensional, Penalized Regression

## Abstract

We present a unified framework for estimation and analysis of generalized additive models in high dimensions. The framework defines a large class of penalized regression estimators, encompassing many existing methods. An efficient computational algorithm for this class is presented that easily scales to thousands of observations and features. We prove minimax optimal convergence bounds for this class under a weak compatibility condition. In addition, we characterize the rate of convergence when this compatibility condition is not met. Finally, we also show that the optimal penalty parameters for structure and sparsity penalties in our framework are linked, allowing cross-validation to be conducted over only a single tuning parameter. We complement our theoretical results with empirical studies comparing some existing methods within this framework.

## 1. Introduction

In this paper, we model a response variable as an additive function of a potentially large number of covariates. The problem can be formulated as follows: we are given n observations with response $y_{i}\in \mathbb{R}$ and covariates $x_{i}\in {\mathbb{R}}^{p}$ for i=1,…,n. The goal is to fit the model

$$g\left(E\left(y_{i}|x_{i}\right)\right)=\beta +\sum_{j=1}^{p}f_{j}\left(x_{ij}\right),\;i=1,\ldots ,n,$$

for a prespecified *link* function g, unknown intercept β and, unknown component functions $f_{1},\ldots ,f_{p}$. The link function, g, is generally based on the outcome data-type, for example, g(x)=x or g(x)=log(x) for continuous or count response data, respectively. The estimands, $f_{1},\ldots ,f_{p}$, give the conditional relationships between each feature $x_{ij}$ and the outcome $y_{i}$ for all i and j. For identifiability, we assume $\sum_{i=1}^{n}f_{j}\left(x_{ij}\right)=0$ for all j=1,…,p. This model is known as a generalized additive model (GAM) (Hastie and Tibshirani, 1990). It extends the generalized linear model (GLM) where each $f_{j}$ is linear, and is a popular choice for modeling different types of response variables as a function of covariates. GAMs are popular because they extend GLMs to model non-linear conditional relationships while retaining some interpretability (we can examine the effect of each covariate $x_{ij}$ individually on $y_{i}$ while holding all other variables fixed); they also do not suffer from the *curse of dimensionality*.

While there are a number of proposals for estimating GAMs, a popular approach is to encode the estimation in the following convex optimization problem:

(1) $$\hat{\beta },{\hat{f}}_{1},\ldots ,{\hat{f}}_{p}\leftarrow \underset{\beta \in \mathbb{R},f_{1},\ldots ,f_{p}\in ℱ}{\text{argmin}}-n^{-1}\sum_{i=1}^{n}\ell \left(y_{i},\beta +\sum_{j=1}^{p}f_{j}\left(x_{ij}\right)\right)+\lambda_{st}\sum_{j=1}^{p}P_{st}\left(f_{j}\right).$$

Here ℱ is some suitable function class; $\ell (y_{i},\theta )$ is the log-likelihood of $y_{i}$ under parameter θ; $P_{st}$ is a structure-inducing penalty to control the wildness of the estimated functions, ${\hat{f}}_{j}$; and $\lambda_{st}>0$ is a penalty parameter which modulates the trade-off between goodness-of-fit and structure/smoothness of estimates. The class ℱ is a general convex space, for example, $ℱ=L^{2}[0,1]$. Functions $-\ell (y_{i},\theta )$ and $P_{st}(f_{j})$ are convex in θ and $f_{j}$, respectively. The objective function in (1) is convex and for small dimension, p, can be solved via a general-purpose convex solver. However, many modern data sets are high-dimensional, often with more features than observations, that is, p>n. Fitting even GLMs is challenging in such settings as conventional methods are known to overfit the data. A common assumption in the high-dimensional setting is *sparsity*, which states that, only a small (but unknown) subset of features is informative for the outcome. In this case, it is desirable to apply feature selection: to build a model for which only a small subset of f^j≡0.

A number of estimators have been proposed for fitting GAMs with sparsity. These estimators are generally solutions to a convex optimization problem. Though they differ in details, we show that most of these optimization problems can be written as:

(2) $$\hat{\beta },{\hat{f}}_{1},\ldots ,{\hat{f}}_{p}\leftarrow \underset{\beta \in \mathbb{R},f_{1},\ldots ,f_{p}\in ℱ}{\text{argmin}}-n^{-1}\sum_{i=1}^{n}\ell \left(y_{i},\beta +\sum_{j=1}^{p}f_{j}\left(x_{ij}\right)\right)+\lambda_{st}\sum_{j=1}^{p}P_{st}\left(f_{j}\right)+\lambda_{sp}\sum_{j=1}^{p}{\left\|f_{j}\right\|}_{n},$$

where ${\left\|f_{j}\right\|}_{n}^{2}=n^{-1}\sum_{i=1}^{n}{\left\{f_{j}\left(x_{ij}\right)\right\}}^{2}$ is a group lasso-type penalty (Yuan and Lin, 2006) for feature-wise sparsity, and $\lambda_{sp}$ a sparsity-related tuning parameter (Ravikumar et al., 2009; Meier et al., 2009; Koltchinskii and Yuan, 2010; Raskutti et al., 2012; Yuan and Zhou, 2016; Lou et al., 2016; Petersen et al., 2016; Sadhanala and Tibshirani, 2019; Tan and Zhang, 2019). However, previous proposals consists of gaps around efficient computation (Koltchinskii and Yuan, 2010; Raskutti et al., 2012; Yuan and Zhou, 2016; Tan and Zhang, 2019) and/or optimal statistical convergence properties (Ravikumar et al., 2009; Lou et al., 2016; Petersen et al., 2016; Sadhanala and Tibshirani, 2019). General-purpose convex solvers have also been suggested (Koltchinskii and Yuan, 2010; Raskutti et al., 2012; Yuan and Zhou, 2016) as an alternative for solving problem (2), but they roughly scale as $O(n^{3}p^{3})$ and are hence inefficient. This manuscript aims to bridge these gaps.

We present a general framework for sparse GAMs with two major contributions, a general algorithm for computing (2) and a theorem for establishing convergence rates. Briefly, our algorithm is based on accelerated proximal gradient descent. This reduces (2) to repeatedly solving a univariate penalized least squares problem. In many cases, this algorithm has a per-iteration complexity of O(np)—precisely that of state-of-the-art algorithms for the lasso (Friedman et al., 2010; Beck and Teboulle, 2009b). Our main theorem establishes fast convergence rates of the form $\text{max}(s\text{ log }p/n,s\xi_{n})$, where s is the number of signal variables and $\xi_{n}$ is the minimax rate of the univariate regression problem, that is, problem (1) with p=1. Nonparametric rates are established for a wide class of structural penalties $P_{st}$ with $\xi_{n}=n^{-2m/\left(2m+1\right)}$, popular choices of $P_{st}$ include m-th order Sobolev and Hölder norms, total variation norm of the m-th derivative and, norms of Reproducing Kernel Hilbert Spaces (RKHS). Parametric rates are also established with $\xi_{n}=T_{n}/n$ via a truncation-penalty; the number of parameters, $T_{n}$, can be fixed or allowed to grow with sample size.

The highlight of this paper is the generality of the proposed framework: not only does it encompass many existing estimators for high-dimensional GAMs, but also estimators for low-dimensional fully nonparametric models and, parametric models in low or high dimensions. Brief examples of our framework’s generalizability include: establishing minimax convergence rates (under well-studied assumptions) where only sub-optimal rates existed (Lou et al., 2016; Ravikumar et al., 2009; Meier et al., 2009; van de Geer, 2010); recovering minimax rates for a general class of loss functions as opposed to only least squares (Raskutti et al., 2012; Yuan and Zhou, 2016; Tan and Zhang, 2019); establishing consistency (albeit at a slower rate) while relaxing strong assumptions on the design matrix and function class (Raskutti et al., 2012; Yuan and Zhou, 2016; Tan and Zhang, 2019).

Extending GAMs (1), to GSAMs (2), appears to simply be a matter of adding a sparsity penalty. However, our manuscript proves a surprising result: sparsity in GAMs can only be achieved if $P_{st}$ is a semi-norm penalty, as opposed to a squared semi-norm. Thus, the originally proposed GAMs (Hastie and Tibshirani, 1990) cannot be extended to highdimensions by simply adding a sparsity penalty. Finally, as a byproduct of our general theorem, we also determine that $\lambda_{st}=\lambda_{sp}^{2}$ in (2), results in optimal convergence rates, reducing the problem to a single tuning parameter. Empirical studies showed a single tuning parameter to yield comparable or better performance compared to finding two tuning parameters over a grid.

The rest of the paper is organized as follows. In Section 2, we detail our framework and discuss various choices of structural penalties, $P_{st}$, illustrating that our framework encompasses many existing proposals. In Section 3 we present an algorithm for solving the optimization problem (2) for a broad class of $P_{st}$ penalties, and establish their theoretical convergence rates in Section 4. We explore the empirical performance of various choices of $P_{st}$ in simulation in Section 5, and in an application to the Boston housing data set and gene expression data sets in Section 6. Concluding remarks are in Section 7.

## 2. General Framework for Additive Models

In this section, we present our general framework for estimating sparse GAMs, discuss its salient features, and review some existing methods as special cases. Before presenting our framework, we introduce some notation. For any function f and response/covariate pair, (y,x), let −ℓ(f)≡−ℓ(y,f(x)) denote a loss function; given data $\left(y_{1},x_{1}\right),\ldots ,\left(y_{n},x_{n}\right)$, let $P_{n}\ell (f)\equiv n^{-1}\sum_{i=1}^{n}\ell \left(y_{i},f\left(x_{i}\right)\right)$ denote an empirical average; and $\|f{\|}_{n}^{2}\equiv n^{-1}\sum_{i=1}^{n}f{\left(x_{i}\right)}^{2}$ denote the empirical norm. With some abuse of notation, we will use the shorthand $f_{j}$ to denote the function $f_{j}◦\pi_{j}$ where $\pi_{j}(x)=x_{j}$ for $x\in {\mathbb{R}}^{p}$.

Our general framework for obtaining a *G**eneralized* *S**parse* *A**dditive* *M**odel* (GSAM) encompasses estimators that can be obtained by solving the following problem:

(3) $$\hat{\beta },{\hat{f}}_{1},\ldots ,{\hat{f}}_{p}\leftarrow \underset{\beta \in \mathbb{R},f_{1},\ldots ,f_{p}\in ℱ}{\text{argmin}}\underset{\text{Goodness}-\text{of}-\text{fit}}{\underset{︸}{-P_{n}\ell \left(\beta +\sum_{j=1}^{p}f_{j}\right)}}+\underset{\text{structure}-\text{inducing}}{\underset{︸}{\lambda^{2}\sum_{j=1}^{p}P_{st}\left(f_{j}\right)}}+\underset{\text{sparsity}-\text{inducing}}{\underset{︸}{\lambda \sum_{j=1}^{p}{\left\|f_{j}\right\|}_{n}}}\cdot$$

This optimization problem balances three terms. The first is a loss function based on goodness-of-fit to the observed data; the least squares loss, $-\ell (f)=(y-f(x){)}^{2}$, is commonly used for continuous response. Our general framework requires only convexity and differentiability of −ℓ(y,θ), with respect to θ. Later we consider loss functions given by the negative log-likelihood of exponential family distributions. The second piece is a penalty to induce smoothness/structure of the function estimates. Our framework requires $P_{st}$ to be a *semi-norm* on ℱ. This choice is motivated by both statistical theory and computational efficiency; we discuss this along with possible choices of $P_{st}$ in the following sub-sections. The final piece is a sparsity penalty $∥\cdot {∥}_{n}$, which encourages models with ${\hat{f}}_{j}\equiv 0$ for many j. For some choices of $P_{st}$ the smoothness and sparsity pattern of $f_{j}$ are intrinsically linked (for example, Ravikumar et al., 2009; Lou et al., 2016); for other structure-inducing penalties the formulation (3) appears to decouple structure and sparsity. However, this manuscript highlights the surprising role of $P_{st}$ in obtaining an appropriate sparsity pattern. Briefly, if $P_{st}$ is a squared norm then either all ${\hat{f}}_{j}\equiv 0$ or all f^j≡0. We detail this result and its extension to semi-norms in Section 2.2. Throughout this manuscript, we require the function class ℱ to be a convex cone, for example, $L^{2}(\mathbb{R})$. Later for some specific results, we will additionally require ℱ to be a linear space.

As noted before, the tuning parameters for structure ($\lambda^{2}$) and sparsity (λ) are coupled in our framework. The theoretical consequence of this is that, for properly chosen λ, we get rate-optimal estimates, up to a constant (details in Section 4). The practical consequence is that we have a single tuning parameter. While fine tuning an estimator with two tuning parameters can lead to improved prediction performance, a single tuning parameter is adequate for most choices of $P_{st}$ as seen in our empirical experiments of Section 5.

Furthermore, our framework relaxes the usual distributional requirements of i.i.d. response from an exponential family; we require only $y_{i}$ independent and $E\{y_{i}-E(y_{i})\}$ to be sub-Gaussian (or sub-Exponential). This demonstrates the generality of our framework and highlights our main innovation: the efficient algorithm of Section 3 and theoretical results of Section 4 apply to a very broad class of estimators, fill in the gaps of existing work and, can easily be applied for the development of future estimators.

### 2.1 Structure Inducing Penalties

We now present some possible choices of the structural penalty $P_{st}$ followed by a discussion of the conditions on $P_{st}$ that lead to desirable estimation and computation. The main requirement is that $P_{st}$ is a semi-norm: a functional that obeys all the rules of a norm except one—for nonzero f we may have $P_{st}(f)=0$. Some potential choices for smoothing semi-norms are:

**Table:** 

1. k-th order Sobolev	$P_{st}\leftarrow P_{\text{sobolev}}\left(f^{\left(k\right)}\right)=\sqrt{\int_{x}{\left\{f^{\left(k\right)}(x)\right\}}^{2}dx}$;
2. k-th order total variation	$P_{st}\leftarrow TV(f^{\left(k\right)})$;
3. k-th order Hölder	$P_{st}\leftarrow P_{\text{holder}}(f^{\left(k\right)})={\text{sup}}_{x}|f^{\left(k\right)}(x)|$;
4. k-th order monotonicity	$P_{st}\leftarrow P_{mon}\left(f^{\left(k\right)}\right)\leftarrow I\left(f;\left\{f:f^{\left(k+1\right)}\geq 0\right\}\right)$;
5. M-th dimensional linear subspace	$P_{st}\leftarrow P_{lin}^{M}(f)=I\left(f;\text{span}\left\{g_{1},\ldots ,g_{M}\right\}\right)$;

here TV(·) is the total variation norm, $TV(f)=\text{sup}\left\{\sum_{i=1}^{o}\left|f\left(z_{i+1}\right)-f\left(z_{i}\right)\right|:z_{1}<\ldots <z_{o}\;\text{is}\;\text{a}\;\text{partition}\;\text{of}\;\left[0,1\right]\right\}$, and I is a convex indicator function defined as I(f;𝒜)=0 if f∈𝒜 and I(f;𝒜)=∞ if f≠𝒜. As implied by the name, $P_{st}$ imposes smoothness or structure on individual components ${\hat{f}}_{j}$. For instance, $P_{\text{sobolev}}(f\prime \prime )$ is a common measure of smoothness; small λ values leads to wiggly fitted functions ${\hat{f}}_{j}$; on the other hand, sufficiently large λ values would lead to each component being a linear function. The convex indicator function, I(·), can impose specific structural properties on ${\hat{f}}_{j}$; for example, $P_{mon}(f)$ fits a model with each ${\hat{f}}_{j}$ a non-decreasing function.

The semi-norm requirement for $P_{st}$ is important because: (a) it implies convexity leading to a convex objective function, (b) the first order absolute homogeneity, $P_{st}(\alpha f)=|\alpha |P_{st}(f)$, is needed for the algorithm of Section 3 and, (c) the triangle inequality is used throughout the proof of our theoretical results of Section 4. For our context, we consider convex indicators of cones as a semi-norm because, the first order homogeneity condition can be relaxed. For our algorithm, we only require $P_{st}(\alpha f)=\alpha P_{st}(f)$ for α>0; for our theoretical results we treat convex indicators of cones as a special case and discuss them at the end of Section 4.2. For non-sparse GAMs of the form (1), the existing literature does not necessarily use a semi-norm penalty; a common choice of smoothing penalty is $P_{st}(f)=P_{\text{sobolev}}^{2}\left(f^{\prime \prime }\right)$. In the following subsection, we discuss the issues with using squared semi-norm penalties in high dimensions, particularly their impact on the sparsity of estimated component functions.

### 2.2 Using a Squared Smoothness Penalty

Given a semi-norm $P_{\text{semi}}$, using $P_{st}=P_{\text{semi}}^{2}$ in (3) may give poor theoretical performance (as noted in Meier et al., 2009, for $P_{\text{semi}}=P_{\text{sobolev}})$ and, can also be computationally expensive (as disscussed in Section 3). In this subsection, we show a surprising result: using a squared semi-norm penalty results in fitting models that are either not sparse (all ${\hat{f}}_{j}\neq 0$) or not flexible (all $f_{j}$ belong to some restrictive parametric class). In other words, the original GAMs (Hastie and Tibshirani, 1990) cannot be extended to high dimensions/sparsity by simply adding a sparsity penalty. This highlights a key contribution of this manuscript: not only do we present a framework for fitting GSAM but also, prove that näıve GAM extensions are not feasible.

In greater detail, using $P_{st}=P_{\text{norm}}^{2}$ where $P_{\text{norm}}$ is a norm, leads to an active set, S={j:f^j≡0}, for which either |S|=0 or |S|=p. If $P_{st}=P_{\text{semi}}^{2}$, for a semi-norm $P_{\text{semi}}$, we can get 0<|S|<p; however, now all ${\hat{f}}_{j}\in {ℱ}_{0}$, where ${ℱ}_{0}$ is a *finite-dimensional* function class. In contrast, using $P_{st}=P_{\text{semi}}$ can give active sets such that 0<|S|<p and each ${\hat{f}}_{j}$ can be modeled nonparametrically.

Before presenting our main results, we note that throughout this section we deal with finite valued semi-norms, this excludes convex indicator penalties. It should be noted that many convex indicator penalties impose a parametric structure, thus excluding them from the discussion of this section. Other convex indicator penalties are challenging to deal with in this context and other settings (see for example, Remark 18). We now present our result in Lemma 1 for a squared norm penalty followed by the extension to squared semi-norms in Corollary 2 (proofs in Appendix D).

**Lemma 1** *Let* ℱ *be a nonparametric function class in the following sense: for any covariate-response pair* $\left(y,x\right)\in {\mathbb{R}}^{n\times 2}$ *there exists some* f∈ℱ *such that* $f(x_{i})=y_{i}$ *for all* i. *Consider the optimization problem*

(4) $${\hat{f}}_{1},\ldots ,{\hat{f}}_{p}\leftarrow \underset{f_{1},\ldots ,f_{p}\in ℱ}{\text{argmin}}\frac{1}{n}\sum_{i=1}^{n}{\left(y_{i}-\sum_{j=1}^{p}f_{j}\left(x_{ij}\right)\right)}^{2}+\sum_{j=1}^{p}\left\{\lambda_{st}P_{st}^{2}\left(f_{j}\right)+\lambda_{sp}{\left\|f_{j}\right\|}_{n}\right\},$$

*for a norm* $P_{st}$ *on* ℱ. *Then*, *for any* $\lambda_{sp}$, *either* ${\hat{f}}_{j}\equiv 0$ *for all* j *or* f^j≡0 *for all* j.

**Corollary 2** *For a semi-norm* $P_{st}$ *in* (4), *we define its null set as* ${ℱ}_{0}\subset ℱ$ *such that* $P_{st}(f_{0})=0$ *for all* $f_{0}\in {ℱ}_{0}$ (*note that* ${ℱ}_{0}$ *contains the zero function*). *Consider an arbitrary*, *non-empty*, *index subset* I⊂{1,…,p}. *If* ${\hat{f}}_{j^{\prime }}=0$ *for all* j′∈I, *then for all* $j\in I^{c}$, ${\hat{f}}_{j}\in {ℱ}_{0}$.

The above results imply that using a squared semi-norm means sacrificing either sparsity or flexibility in our modeling approach. In most cases the set ${ℱ}_{0}$ is parametric, e.g, a commonly used penalty $P_{st}=P_{\text{sobolev}}(f\prime \prime )$, leads to ${ℱ}_{0}$ as the set of linear functions. Thus we can either fit sparse, parametric models or non-sparse, nonparametric models but *not both*. In other words, for parametric regression we can simply add a sparsity-inducing penalty; for example, the elastic net (Zou and Hastie, 2005), which adds a sparsity penalty to ridge regression, leading to sparse linear models. In contrast, simply adding a sparsity penalty to traditional GAMs (Hastie and Tibshirani, 1990) is not sufficient because, fitted models will not be sparse GAMs.

### 2.3 Relationship of Existing Methods to GSAM

We now discuss some of the existing methods for sparse additive models in greater detail, and demonstrate that many existing proposals are special cases of our GSAM framework. One of the first proposals for sparse additive models, SpAM (Ravikumar et al., 2009), uses a basis expansion and solves

$$\underset{\beta_{1},\ldots ,\beta_{j}\in {\mathbb{R}}^{M}}{\text{argmin}}{\left\|y-\sum_{j=1}^{p}\sum_{m=1}^{M}\beta_{jm}\psi_{jm}\right\|}_{n}^{2}+\lambda \sum_{j=1}^{p}{\left\|\sum_{m=1}^{M}\beta_{jm}\psi_{jm}\right\|}_{n},$$

where $\psi_{jm}={\left[\psi_{m}\left(x_{1j}\right),\ldots ,\psi_{m}\left(x_{nj}\right)\right]}^{⊤}\in {\mathbb{R}}^{n}$ for basis functions $\psi_{1},\ldots ,\psi_{M}$. This is a GSAM with $P_{st}=I\left(f;\text{span}\left\{\psi_{1},\ldots ,\psi_{M}\right\}\right)$. The SpAM proposal is extended to partially linear models in SPLAM (Lou et al., 2016). There, a similar basis expansion is used, though with the particular choice $\psi_{1}(x)=x$. The SPLAM estimator solves

$$\underset{\beta_{1},\ldots ,\beta_{j}\in {\mathbb{R}}^{M}}{\text{argmin}}{\left\|y-\sum_{j=1}^{p}\sum_{m=1}^{M}\beta_{jm}\psi_{jm}\right\|}_{n}^{2}+\lambda_{1}\sum_{j=1}^{p}{\left\|\sum_{m=1}^{M}\beta_{jm}\psi_{jm}\right\|}_{n}+\lambda_{2}\sum_{j=1}^{p}{\left\|\sum_{m=2}^{M}\beta_{jm}\psi_{m}\right\|}_{n},$$

and is also a GSAM with

$$P_{st}=I\left(f;\text{span}\left\{\psi_{1},\ldots ,\psi_{M}\right\}\right)+\sum_{j=1}^{p}{\left\|{\text{Proj}}_{\text{span}\left(\psi_{2},\ldots ,\psi_{M}\right)}(f)\right\|}_{n},$$

where ${\text{Proj}}_{A}$ is the projection operator onto the set A. The recently proposed extensions of trend filtering to additive models are other examples (Petersen et al., 2016; Sadhanala and Tibshirani, 2019); these methods can be written in our GSAM framework with $P_{st}(f)=TV(f)$.

Meier et al. (2009) give two proposals: the first solves the optimization problem

$$\underset{f_{1},\ldots ,f_{p}\in ℱ}{\text{argmin}}{\left\|y-\sum_{j=1}^{p}f_{j}\right\|}_{n}^{2}+\sum_{j=1}^{p}\lambda_{sp}\sqrt{{\left\|f_{j}\right\|}_{n}^{2}+\lambda_{st}P_{st}^{2}\left(f_{j}\right)},$$

and is not a GSAM; they note that this proposal gives a suboptimal rate of convergence. The second is a GSAM of the form (3) with $P_{st}(f)=P_{\text{sobolev}}(f\prime \prime )$. At the time, Meier et al. (2009) focused on the first proposal as no computationally efficient method for solving the second one was known to them. In a follow-up paper, van de Geer (2010) studied the theoretical properties of a GSAM with an alternative, *diagonalized smoothness* structural penalty. The diagnolized smoothness penalty for a function with basis expansion $f_{\beta }(x)=\sum_{j=1}^{n}\psi_{j}\left(x\right)\beta_{j}$, is defined as

$$P_{st}\left(f_{\beta }\right)={\left(\sum_{j=1}^{n}j^{2m}\beta_{j}^{2}\right)}^{1/2},$$

for a smoothness parameter m.

Koltchinskii and Yuan (2010), Raskutti et al. (2012) and Yuan and Zhou (2016) discuss a similar framework to GSAMs; however, they only consider structural penalties $P_{st}$, which are norms of Reproducing Kernel Hilbert Spaces (RKHS). Furthermore, they do not discuss efficient algorithms for solving the convex optimization problem. Using properties of RKHS, they note that their estimator is the minimum of a d=np dimensional second order cone program (SOCP). The computation for general-purpose SOCP solvers scales roughly as $d^{3}$. Thus for even moderate p and n, these problems quickly become intractable. Recently, Tan and Zhang (2019) studied GSAMs beyond the RKHS framework similar to this manuscript, however, there are important differences. Firstly, Tan and Zhang (2019) consider only the least squares loss. Secondly, the authors do not present an algorithm for estimation; instead they prove that under a least squares loss and special smoothness penalties the solution to the optimization problem is finite dimensional. Thirdly, their results (like the minimax rates proved by Yuan and Zhou, 2016) include the more general notion of *weak sparsity* (van de Geer, 2016); however, extending this notion beyond the least squares loss is left for future research. All of the above mentioned proposals either fail to provide an efficient computational algorithm or have sub-optimal convergence rates. There are also a number of other proposals that do not quite fall in the GSAM framework (Chouldechova and Hastie, 2015; Fan et al., 2012; Yin et al., 2012).

## 3. General-Purpose Algorithm

Here we give a general algorithm for fitting GSAMs based on proximal gradient descent (Parikh and Boyd, 2014). We begin with some notation. We denote by $\dot{\ell }(y,\theta )$ and $\ddot{\ell }(y,\theta )$ the first and second derivatives of ℓ with respect to θ. For functions $f,g:{\mathbb{R}}^{p}\to \mathbb{R}$, let $\langle f,\dot{\ell }(g)\rangle_{n}\equiv n^{-1}\sum_{i=1}^{n}f\left(x_{i}\right)\left\{\dot{\ell }\left(y_{i},g\left(x_{i}\right)\right)\right\}$, $P_{n}\dot{\ell }(g)\equiv n^{-1}\sum_{i=1}^{n}\dot{\ell }\left(y_{i},g\left(x_{i}\right)\right)$ and, $\|f+\dot{\ell }(g){\|}_{n}^{2}\equiv n^{-1}\sum_{i=1}^{n}{\left\{f\left(x_{i}\right)+\dot{\ell }\left(y_{i},g\left(x_{i}\right)\right)\right\}}^{2}$.

We begin with a second order Taylor expansion of the loss at some arbitrary point $\beta^{0}+\sum_{j=1}^{p}f_{j}^{0}$. For this, we first apply Taylor’s theorem to $\ell (y_{i},\beta +\theta_{i1}+\ldots +\theta_{ip})$ as a (p+1) variate function of $\left(\beta ,\theta_{i1},\ldots ,\theta_{ip}\right)$. Note that for $|\ddot{\ell }(y,\theta )|\leq L$, the Hessian matrix, $H_{p+1}$, of $\ell (y_{i},\beta +\theta_{i1}+\ldots +\theta_{ip})$ obeys the inequality $a^{T}H_{p+1}a\leq (p+1)L\|a{\|}_{2}^{2}$ for all $a\in {\mathbb{R}}^{p+1}$ (Zhan, 2005). This gives us the following bound:

$$-P_{n}\ell \left(\beta +\sum_{j=1}^{p}f_{j}\right)\leq -P_{n}\ell \left(\beta^{0}+\sum_{j=1}^{p}f_{j}^{0}\right)-\left(\beta -\beta^{0}\right)P_{n}\dot{\ell }\left(\beta^{0}+\sum_{j=1}^{p}f_{j}^{0}\right)-\sum_{j=1}^{p}{\langle f_{j}-f_{j}^{0},\dot{\ell }\left(\beta^{0}+\sum_{j=1}^{p}f_{j}^{0}\right)\rangle }_{n}+\frac{(p+1)L}{2}{\left(\beta -\beta^{0}\right)}^{2}+\sum_{j=1}^{p}\frac{(p+1)L}{2}{\left\|f_{j}-f_{j}^{0}\right\|}_{n}^{2},$$

which leads to the following majorizing inequality

(5) $$-P_{n}\ell \left(\beta +\sum_{j=1}^{p}f_{j}\right)\leq \frac{(p+1)L}{2}{\left[\beta -\left\{\beta^{0}+\frac{1}{(p+1)L}P_{n}\dot{\ell }\left(\beta^{0}+\sum_{j=1}^{p}f_{j}^{0}\right)\right\}\right]}^{2}+\sum_{j=1}^{p}\frac{(p+1)L}{2}{\left\|f_{j}-\left\{f_{j}^{0}+\frac{1}{(p+1)L}\dot{\ell }\left(\beta^{0}+\sum_{j=1}^{p}f_{j}^{0}\right)\right\}\right\|}_{n}^{2}+W,$$

where W is not a function of β or $f_{j}$ for any j. Instead of minimizing the original problem (3), we minimize the majorizing surrogate

(6) $$\frac{1}{2}{\left[\beta -\left\{\beta^{0}+tP_{n}\dot{\ell }\left(\beta^{0}+\sum_{j=1}^{p}f_{j}^{0}\right)\right\}\right]}^{2}+\frac{1}{2}\sum_{j=1}^{p}{\left\|f_{j}-\left\{f_{j}^{0}+t\dot{\ell }\left(\beta^{0}+\sum_{j=1}^{p}f_{j}^{0}\right)\right\}\right\|}_{n}^{2}+t\lambda^{2}\sum_{j=1}^{p}P_{st}\left(f_{j}\right)+t\lambda \sum_{j=1}^{p}{\left\|f_{j}\right\|}_{n},$$

where $t=\{(p+1)L{\}}^{-1}$. Minimizing (6) and re-centering our Taylor series at the current iteration, is precisely the proximal gradient recipe. Updating the intercept β, is simply $\hat{\beta }\leftarrow \beta^{0}+tP_{n}\dot{\ell }\left(\beta^{0}+\sum_{j=1}^{p}f_{j}^{0}\right)$. Components $f_{1},\ldots ,f_{p}$, can be updated in parallel by solving the univariate problems:

(7) $${\hat{f}}_{j}\leftarrow \underset{f\in ℱ}{\text{argmin}}\frac{1}{2}{\left\|\left\{f_{j}^{0}+t\dot{\ell }\left(\beta^{0}+\sum_{j=1}^{p}f_{j}^{0}\right)\right\}-f\right\|}_{n}^{2}+t\lambda^{2}P_{st}\left(f\right)+t\lambda \|f{\|}_{n}.$$

At first, this problem still appears difficult due to the combination of structure and sparsity penalties. However, the following Lemma shows that things greatly simplify.

**Lemma 3** *Suppose* $P_{st}$ *is a semi-norm*, *and* r *is an n-vector*. *Consider the optimization problems*

(8) $$\underset{f\in ℱ}{\text{argmin}}\frac{1}{2}\|r-f{\|}_{n}^{2}+\lambda_{1}P_{st}(f)+\lambda_{2}\|f{\|}_{n},$$

(9) $$\underset{f\in ℱ}{\text{argmin}}\frac{1}{2}\|r-f{\|}_{n}^{2}+\lambda_{1}P_{st}(f).$$

*If* $\tilde{f}$ *is a solution to* (9)*; then* $\hat{f}$ *is a solution to* (8) *where* $\hat{f}$ *is defined as*

(10) $$\hat{f}={\left(1-\lambda_{2}/\|\tilde{f}{\|}_{n}\right)}_{+}\tilde{f},$$

*with* $\left(z{)}_{+}=\text{max}(z,0\right)$. *Additionally*, *if* $\lambda_{2}\geq ∥r{∥}_{n}$, *then* $\hat{f}\equiv 0$.

The proof is given in Appendix E. Using Lemma 3, we can get the solution to (7) by solving a problem in the form of (9), a classical univariate smoothing problem, and then applying (10), the simple soft-scaling operator. Putting this together, leads to Algorithm 1 below for solving (3). The general recipe used to derive Algorithm 1, is the well known proximal gradient descent algorithm. Thus, well established convergence results in the literature can be used (see for example, Beck and Teboulle, 2009b), under mild conditions: we require a convex ℓ with Lipschitz first derivative. These conditions hold for many loss functions particularly the negative log-likelihood of exponential family distributions. Convergence of the infinite-dimensional optimization over ℱ follows from the finite-dimensional analog of problem (3); we prove this in Appendix E.

Algorithm 1 is simple and can be quite fast: the time complexity is largely determined by the difficulty of solving the univariate smoothing problem of step 5. In many cases this takes O(n) operations, allowing an iteration of proximal gradient descent to run in O(np) operations. Complexity order O(np) is the per-iteration time complexity of state-of-the-art algorithms for the lasso (Friedman et al., 2010; Beck and Teboulle, 2009a).

Any step-size t can be used in Algorithm 1 so long as inequality (5) holds for $f_{j}^{0}\equiv f_{j}^{k-1}$ and $f_{j}\equiv f_{j}^{k}$ when (p+1)L is replaced by $t^{-1}$. Note that if $t\leq \{L(p+1){\}}^{-1}$ this will always hold. While a fixed step size t ensures theoretical convergence, in practice we can achieve a substantial speedup by adaptively selecting t for each iteration. We could use $t_{k}={\left\{L\left(p_{\text{active}}^{k}+1\right)\right\}}^{-1}$, where $p_{\text{active}}^{k}$ is the number of j for which either of $f_{j}^{k-1}$ or $f_{j}^{k}$ is non-zero. Since we are interested in sparse models, generally $p_{\text{active}}^{k}\ll p$, leading to a substantial efficiency gain. If we do not have a suitable bound for L, we could use a datadependent scheme such as the backtracking line search (Beck and Teboulle, 2009b). The algorithm can also take advantage of Nesterov-style acceleration (Nesterov, 2007), which improves the worst-case convergence rate after k steps from $O(k^{-1})$ or $O(k^{-2})$.

An important special case is the least squares loss $-\ell (y,\theta )=(y-\theta {)}^{2}$. In this case, we can use a block coordinate descent algorithm which can be more efficient than Algorithm 1, and does not require a step-size calculation. We present the full details of the algorithm in Appendix A.


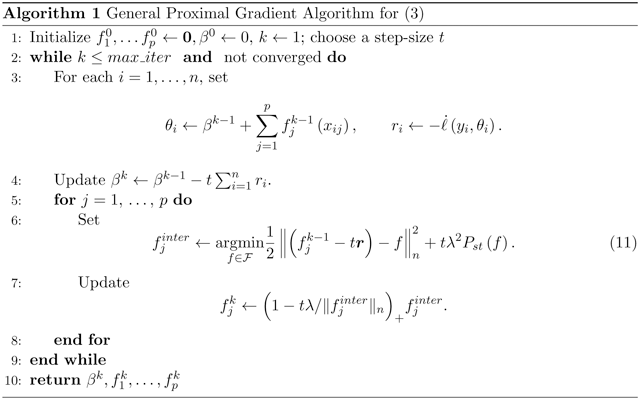


Algorithm 1 is developed for a given λ value. Alternatively, we recommend using a decreasing sequence of λ values, linear on the log scale starting at $\lambda_{\text{max}}=∥y{∥}_{n}$. Another computational consideration is the method of tuning parameter selection: our numerical experiments suggest K-fold cross validation as a suitable choice; however, we note that other tuning parameter selection techniques such as generalized cross validation, AIC and BIC can be used. Finding a theoretically optimal tuning parameter method, or proving the estimator selected by cross validation obtains the same fast convergence rate, is a challenging problem which we defer to future work.

As noted above, the main computational hurdle in Algorithm 1 is solving the univariate problem (9). In the following subsection, we discuss this step in greater detail for various smoothness penalties.

### 3.1 Solving the Univariate Subproblem

For many semi-norm smoothers there are already efficient solvers for solving (9): with the k-th order total variation penalty, (9) can be solved exactly in 2n operations for k=0 (Johnson, 2013), or iteratively in roughly O((k+1)n) operations for k≥1 (Ramdas and Tibshirani, 2015); with the convex indicator of an M-dimensional linear subspace, (9) can be solved in $O(M^{2}n)$ operations using linear regression; using a monotonicity indicator, (9) can be solved with the pool adjacent violators algorithm in O(n) operations (Ayer et al., 1955).

For many other choices of $P_{st}$, we do not have efficient algorithms for solving (9); however, we might have fast algorithms for the slightly different optimization problem:

(12) $${\tilde{f}}_{\tilde{\lambda }}\leftarrow \underset{f\in ℱ}{\text{argmin}}\frac{1}{2}\|r-f{\|}_{n}^{2}+\tilde{\lambda }P_{st}^{\tau }\left(f\right),$$

for τ>1. For example, the k-th order Sobolev penalty (Wahba, 1990) can be solved exactly in O(kn) operations for τ=2. In the following Lemma, we show that the solution to (12) can be leveraged to solve the harder problem (9).

**Lemma 4** *Given an n-vector* r, *a convex linear space* ℱ *over the field* ℝ, *and real* τ>1, *consider the optimization problems:*

$${\hat{f}}_{\lambda }\leftarrow \underset{f\in ℱ}{\text{argmin}}\frac{1}{2}\|r-f{\|}_{n}^{2}+\lambda P_{st}\left(f\right);$$

$${\tilde{f}}_{\lambda }\leftarrow \underset{f\in ℱ}{\text{argmin}}\frac{1}{2}\|r-f{\|}_{n}^{2}+\lambda P_{st}^{\tau }\left(f\right);$$

$$f_{\text{null}}\leftarrow \underset{f\in ℱ}{\text{argmin}}\frac{1}{2}\|r-f{\|}_{n}^{2}+I\left(f\in ℱ:P_{st}\left(f\right)=0\right);$$

$$f_{\text{interp}}\leftarrow \underset{f\in ℱ}{\text{argmin}}P_{st}^{\tau }(f)+I\left(r_{i}=f\left(x_{i}\right)\;for\;all\;i\right),$$

*where* $P_{st}(\cdot )$ *is a semi-norm on* ℱ. *Assume that the directional derivative*

$$\nabla_{h}P_{st}^{\tau }(f)=\underset{\varepsilon \to 0}{\text{lim}}\frac{P_{st}^{\tau }(f+\varepsilon h)-P_{st}^{\tau }(f)}{\varepsilon },$$

*exists for all* h∈ℱ. *If* $P_{st}\left({\hat{f}}_{\lambda }\right)\neq 0$ *and* $\tau \tilde{\lambda }P_{st}^{\tau -1}\left({\tilde{f}}_{\tilde{\lambda }}\right)=\lambda$, *then* ${\hat{f}}_{\lambda }={\tilde{f}}_{\tilde{\lambda }}$.

*To determine if* $P_{st}(\hat{f})=0$, *let* $ℱ={ℱ}_{1}⊕{ℱ}_{2}$, *where* ⊕ *is such that*, *for all* f∈ℱ *we have* $f=f_{0}+f_{⊥}$ *where* $\langle f_{0},f_{⊥}\rangle_{n}=0$ *and* $P_{st}(f)=P_{st}(f_{⊥})$. *Furthermore*, *let* $P_{st}^{*}$ *be the dual norm over* ${ℱ}_{2}$, *given by*

(13) $$P_{st}^{*}\left(f_{⊥}\right)=\text{sup}\;\left\{\left|{\langle f_{⊥},f_{⊥}^{\prime }\rangle }_{n}\right|:P_{st}\left(f_{⊥}^{\prime }\right)\leq 1,f_{⊥}^{\prime }\in {ℱ}_{2}\right\}.$$

*Then* $f_{\text{interp}}-f_{\text{null}}\in {ℱ}_{2}$ *and* ${\hat{f}}_{\lambda }=f_{\text{null}}$ *if* $\lambda \geq P_{st}^{*}\left(f_{\text{interp}}-f_{\text{null}}\right)$.

The proof is given in Appendix E. This lemma allows us to first check if we should shrink entirely to a null fit with $P_{st}(\hat{f})=0$ (usually a finite dimensional function), based on the dual semi-norm of the interpolating function $f_{\text{interp}}$. If we do not shrink to $P_{st}(\hat{f})=0$, then there is an equivalence between $\hat{f}$ and $\tilde{f}$; and the problem is reduced to finding $\tilde{\lambda }$ with $\tau \tilde{\lambda }P_{st}^{\tau -1}\left({\tilde{f}}_{\tilde{\lambda }}\right)=\lambda$ for the originally specified λ. This can be done in a number of ways; most simply by a combination of grid search and then local bisection noting that a) we need not try any $\tilde{\lambda }$-values above $\lambda_{max}\equiv ∥f_{\text{interp}}{∥}_{n}$ (by Lemma 3), and b) $\tilde{\lambda }P_{st}\left({\tilde{f}}_{\tilde{\lambda }}\right)$ is a smooth function of $\tilde{\lambda }$. In fact, the grid search will often be unnecessary as we will generally have a good guess from the previous iterate of the proximal gradient algorithm, and can leverage the fact that $P_{st}\left({\tilde{f}}_{\tilde{\lambda }}\right)$ and $P_{st}\left({\hat{f}}_{\lambda }\right)$ are both smooth functions of r. To assess the computational impact of an added grid search, we looked at the run-time for the proximal problem with $P_{st}=P_{\text{sobolev}}$ (which requires a grid search) and with $P_{st}\in \{TV(f^{\left(0\right)}),TV(f^{\left(1\right)}),TV(f^{\left(2\right)})\}$ (which does not use Lemma 4). For 100 replications of the proximal problem on a quadcore Intel^®^Core^™^, i7-10510U CPU @1.80GHz, the median run-time with n=500 for $P_{st}=P_{\text{sobolev}}$ was 693.20 μs. In contrast, the median run-time for $P_{st}(f)=TV(f^{\left(k\right)})$ for k=0,1,2 was 514.15, 2968.30, 4884.90 μs, respectively. These median run-times were calculated via a small simulation study; details of this experiment along with detailed timing results are presented in Appendix B.

To complete the discussion, we give the explicit form of the dual norm (13) for the case where $P_{st}(f)=\|D\vec{f}{\|}_{q}$ for a matrix $D\in {\mathbb{R}}^{M\times n}$, a vector $\vec{f}={\left[f\left(x_{1}\right),\ldots ,f\left(x_{n}\right)\right]}^{⊤}\in {\mathbb{R}}^{n}$, and q≥1. Such penalties are common in the literature, for example, when $P_{st}$ is the Sobolev semi-norm, total variation norm, or any RKHS norm. For $P_{st}(f)=\|D\vec{f}{\|}_{q}$, the dual norm is given by

$$P_{st}^{*}(f)={\left\|D{\left(D^{⊤}D\right)}^{-}\vec{f}\right\|}_{\tilde{q}},$$

where $(D^{⊤}D{)}^{-}$ is the Moore-Penrose pseudo inverse of $D^{⊤}D$ and $\tilde{q}$ satisfies $1/q+1/\tilde{q}=1$.

## 4. Theoretical Results

Here we prove rates of convergence for GSAMs, estimators that fall within our framework (3). We first present the so-called *slow rates*, which require few assumptions, followed by *fast rates*, which require compatibility and margin conditions (defined and discussed below). Our fast rates match the minimax rates under Gaussian data with a least squares loss (Raskutti et al., 2009) and, our slow rates can be seen as an additive generalization of the lasso slow rates (Dalalyan et al., 2017). For both slow and fast rates, we first present a deterministic result; this result simply states that if we are within a special set, 𝒯, then the convergence rates hold. We then show that under suitable conditions (stated and discussed below) on the loss function, smoothness penalty, and data, we lie in 𝒯 with high probability. Throughout, we also allow for mean model misspecification with an additional *approximation error* term in the convergence rates; if the true mean model is additive, then this term disappears.

To the best of our knowledge, the closest results to our work were established by Koltchinskii and Yuan (2010). However, they consider a more restrictive setting of Reproducing Kernel Hilbert Spaces (RKHS); where each additive component $f_{j}$ belongs to a RKHS ${ℋ}_{j}$, and $P_{st}$ is the norm on ${ℋ}_{j}$. Our work gives these rates for all semi-norm penalties and function classes ℱ, associated with certain non-restrictive entropy conditions. Before presenting the main results, we present some notation and definitions which will be used throughout the section.

### 4.1 Definitions and Notation

We consider here properties of the solution to

(14) $$\hat{\beta },{\hat{f}}_{1}\ldots ,{\hat{f}}_{p}\leftarrow \underset{\beta \in ℛ,{\left\{f_{j}\right\}}_{j=1}^{p}\in ℱ}{\text{argmin}}-P_{n}\ell \left(\beta +\sum_{j=1}^{p}f_{j}\right)+\lambda \sum_{j=1}^{p}\left\{{\left\|f_{j}\right\|}_{n}+\lambda P_{st}\left(f_{j}\right)\right\},$$

where ℛ⊆ℝ and ℱ is some univariate function class. Note that in (14) we optimize β over ℛ; this is because we need ℛ to be a bounded for proving the slow rates, the stronger *compatibility condition* allows us to take ℛ=ℝ for proving fast rates.

For a function $f(x)=\beta +\sum_{j=1}^{p}f_{j}\left(x_{j}\right)$ we use the shorthand notation

$$I(f)\equiv \sum_{j=1}^{p}\left\{{\left\|f_{j}\right\|}_{n}+\lambda P_{st}\left(f_{j}\right)\right\},$$

which defines a semi-norm on the function f. Furthermore, for any index set S⊂{1,…,p} we define $I_{S}(f)$ as $I_{S}(f)=\sum_{j\in S}\left\{{\left\|f_{j}\right\|}_{n}+\lambda P_{st}\left(f_{j}\right)\right\}$. We denote the target function by $f^{0}$ where

$$f^{0}\leftarrow \underset{f\in {ℱ}^{0}}{\text{arg}\;\text{min}}-P\ell (f),$$

for some function class ${ℱ}^{0}$ and, where $P\ell (f)=n^{-1}\sum_{i=1}^{n}E\left\{\ell \left(y_{i},f\left(x_{i}\right)\right)\right\}$. We say the target function belongs to some class ${ℱ}^{0}$ to signify that $f^{0}$ does not need to belong to ℱ. We require no assumptions on the class ${ℱ}^{0}$ for the slow-rates of Theorem 7; we can take ${ℱ}^{0}$ to be the class of all measurable functions. For the fast rates we will require the margin condition on a subset of ${ℱ}^{0}$.

We define the *excess risk* for a function f as $ℰ(f)=P\left\{\ell \left(f^{0}\right)-\ell (f)\right\}$, and we denote by $\nu_{n}(\cdot )$ the *empirical process term*, which is defined as

$$\nu_{n}(f)=\left(P_{n}-P\right)\{-\ell (f)\}=-\frac{1}{n}\sum_{i=1}^{n}\left\{\ell \left(y_{i},f\left(x_{i}\right)\right)-E\ell \left(y_{i},f\left(x_{i}\right)\right)\right\}.$$

Define the δ-*covering number*, $N(\delta ,ℱ,∥\cdot {∥}_{Q})$, as the size of the smallest δ-cover of ℱ with respect to the norm $∥\cdot {∥}_{Q}$ induced by measure Q. We denote the δ-*entropy* of ℱ by $H(\delta ,ℱ,∥\cdot {∥}_{Q})\equiv \text{log}N(\delta ,ℱ,∥\cdot {∥}_{Q})$. Given fixed covariates $x_{1},\ldots ,x_{n}\in {\mathbb{R}}^{p}$, we denote the empirical measure by $Q_{n}$ where $Q_{n}=n^{-1}\sum_{i=1}^{n}\delta_{x_{i}}$, and for covariate j; we denote by $Q_{j,n}$ the empirical measure of $\left(x_{1,j},\ldots ,x_{n,j}\right)$. We define two different types of entropy bounds for a function class ℱ.

**Definition 5 (Logarithmic Entropy)** *A univariate function class*, ℱ, *is said to have a logarithmic entropy bound if*, *for all* j=1,…,p, *and* γ>0, *we have*

(15) $$H\left(\delta ,\left\{f_{j}\in ℱ:{\left\|f_{j}\right\|}_{n}+\gamma P_{st}\left(f_{j}\right)\leq 1\right\},\|\cdot {\|}_{Q_{j,n}}\right)\leq A_{0}T_{n}\;\text{log}\;\left(1/\delta +1\right),$$

*for some constant* $A_{0}$, *and parameter* $T_{n}$.

**Definition 6 (Polynomial Entropy with Smoothness)** *A univariate function class*, ℱ, *is said to have a polynomial entropy bound with smoothness if*, *for all* j=1,…,p *and* γ>0, *we have*

(16) $$H\left(\delta ,\left\{f_{j}\in ℱ:\|f_{j}{\|}_{n}+\gamma P_{st}\left(f_{j}\right)\leq 1\right\},\|\cdot {\|}_{Q_{j,n}}\right)\leq A_{0}{\left(\delta \gamma \right)}^{-\alpha },$$

*for some constant* $A_{0}$, *parameter* α∈(0,2).

The concept of entropy is commonly used in the literature, particularly in nonparametric statistics and empirical processes, to quantify the size of function classes. The logarithmic entropy bound (15) holds for most finite dimensional classes of dimension $T_{n}$. For instance, it holds for $ℱ=L^{2}(\mathbb{R})$ with $P_{st}\left(f_{j}\right)=I\left(f_{j};\text{span}\left\{x,x^{2},\ldots ,x^{T_{n}}\right\}\right)$. The bound (16) commonly holds for broader function classes, for example, for $ℱ=L^{2}([0,1])$ with $P_{st}(f_{j})=P_{\text{sobolev}}(f^{\left(k\right)})$ and α=1/k.

To simplify our presentation of bounds on the convergence rate, we use A≲B to denote A≤cB for some constant c>0. We write A≍B if A≲B and B≲A.

### 4.2 Main Results

We now present our main results: upper bounds for the excess risk of GSAMs, specifically, bounds for $ℰ\left(\hat{\beta }+\sum_{j=1}^{p}{\hat{f}}_{j}\right)$. The following theorem shows that $ℰ\left(\hat{\beta }+\sum_{j=1}^{p}{\hat{f}}_{j}\right)≾\lambda$ over a special set 𝒯. In the corollary that follows, we show that for appropriate λ values, and certain type of loss functions, we are within 𝒯 with high probability.

**Theorem 7 (Slow Rates for GSAM)** *Let* $\hat{f}=\hat{\beta }+\sum_{j=1}^{p}{\hat{f}}_{j}$ *be as defined in* (14), *and let* $f^{*}=\beta^{*}+\sum_{j=1}^{p}f_{j}^{*}$ *be an arbitrary additive function with* $\sum_{i=1}^{N}f_{j}^{*}\left(x_{ij}\right)=0$ *and* $\beta^{*}\in ℛ$. *Assume that* −ℓ(·) *and* $P_{st}$ *are convex and that* ${\text{sup}}_{\beta \in ℛ}|\beta |<R$. *Define* $M^{*}$ *such that*

$$\rho M^{*}=ℰ\left(f^{*}\right)+2\lambda I\left(f^{*}\right)+2R\rho ,$$

*where* λ≥4ρ. *Furthermore*, *define the set* 𝒯 *as follows*

$$𝒯=\left\{Z_{M^{*}}\leq \rho \left(M^{*}+2R\right)\right\},\;\text{where}\;Z_{M^{*}}=\underset{I\left(f-f^{*}\right)\leq M^{*}}{\text{sup}}\left|\nu_{n}(f)-\nu_{n}\left(f^{*}\right)\right|.$$

*Then*, *on the set* 𝒯,

$$ℰ(\hat{f})+\lambda I\left(\hat{f}-f^{*}\right)\leq \rho M^{*}+\rho (2R)+2\lambda I\left(f^{*}\right)+ℰ\left(f^{*}\right).$$

**Corollary 8** *Let* $\hat{f}$, $f^{*}$ *and* ℛ *be as defined in Theorem 7*. *Assume that for any function* f *the loss* ℓ(·) *is such that*

$$-\ell (f)=-\ell \left(y_{i},f\left(x_{i}\right)\right)=ay_{i}f\left(x_{i}\right)+b\left(f\left(x_{i}\right)\right),$$

*for some* a∈ℝ\{0} *and function* b:ℝ→ℝ. *Further assume that for* i=1,…,n, $y_{i}-E\left(y_{i}\right)$ *are independent*, *uniformly sub-Gaussian:*

$$\underset{i=1,\ldots ,n}{\text{max}}K^{2}\left[E\text{exp}{\left\{y_{i}-E\left(y_{i}\right)\right\}}^{2}/K^{2}-1\right]\leq \sigma_{0}^{2}.$$

*Finally*, *suppose* $ℰ(f^{*})=O(\lambda )$ *and* $I(f^{*})=O(1)$. *Then*, *with probability at-least* $1-2\text{ exp}(-C_{1}n\rho^{2})–C\text{ exp}(-C_{1}n\rho^{2})$, *we have the following cases:*

1. *If* ℱ *has a logarithmic entropy bound*, *then for* $\lambda ≍\rho ≍\kappa \;\text{max}\left(\sqrt{\frac{T_{n}}{n}},\sqrt{\frac{\text{log}\;p}{n}}\right)$,
$$ℰ(\hat{f})+\lambda I\left(\hat{f}-f^{*}\right)≾\text{max}\left(\sqrt{\frac{T_{n}}{n}},\sqrt{\frac{\text{log}\;p}{n}}\right),$$
  *with constants* $\kappa =\kappa (a,K,\sigma_{0},A_{0})$, $C_{1}=C_{1}(K,\sigma_{0})$, $C=C(K,\sigma_{0})$ *and* $C_{2}=C_{2}(C,\kappa )$.
2. *if* ℱ *has a polynomial entropy with smoothness*, *then for* $\lambda ≍\rho ≍\kappa \;\text{max}\left(n^{-\frac{1}{2+\alpha }},\sqrt{\frac{\text{log}\;p}{n}}\right)$,
$$ℰ(\hat{f})+\lambda I\left(\hat{f}-f^{*}\right)≾\text{max}\;\left(n^{-\frac{1}{2+\alpha }},\sqrt{\frac{\text{log}\;p}{n}}\right)$$
  *with constants* $\kappa =\kappa (a,K,\sigma_{0},A_{0},\alpha )$, $C_{1}=C_{1}(K,\sigma_{0})$, $C=C(K,\sigma_{0})$ *and* $C_{2}=C_{2}(C,\kappa )$.

In the above corollary, the assumption $I(f^{*})=O(1)$ is often reasonable in high-dimensions; if omitted, with the same high probability, the above rates will be multiplied by the term $\sum_{j=1}^{p}{\left\|f_{j}^{*}\right\|}_{n}$. Now for high-dimensions, we commonly assume sparsity, $f^{*}=\sum_{j\in S}f_{j}^{*}$, where |S| is small. The dependence of the rate on sparsity can be directly expressed by the inequality $\sum_{j\in S}{\left\|f_{j}^{*}\right\|}_{n}\leq |S|{\text{max}}_{j\in S}{\left\|f_{j}^{*}\right\|}_{n}$. Another possible assumption for high dimensions is *weak sparsity*, which states that, the effect size of most component functions is very small. In this case, the preceding inequality would not be tight but we essentially have $\sum_{j=1}^{p}{\left\|f_{j}^{*}\right\|}_{n}=O(1)$.

We now proceed to show the fast rates of convergence. To establish these rates, we require the *compatibility* and *margin* conditions. The compatibility condition, is based on the idea that I(f) and ∥f∥ are somehow compatible for some norm ∥·∥. This condition is common in the high-dimensional literature for proving fast rates (see van de Geer and Bühlmann, 2009, for a discussion of compatibility and related conditions for the lasso). The margin condition, is based the idea that if ℰ(f) is small then $∥f-f^{0}∥$ should also be small. This is another common condition in the literature for handling general convex loss functions (see for example, Negahban et al., 2011; van de Geer, 2008).

**Definition 9 (Compatibility Condition)** *The compatibility condition is said to hold for an index set* S⊂{1,2,…,p}, *with compatibility constant* ϕ(S)>0, *if for all* γ>0 *and all functions* f *of the form* $f(x)=\beta +\sum_{j=1}^{p}f_{j}\left(x_{j}\right)$ *that satisfy* $\sum_{j\in S^{c}}{\left\|f_{j}\right\|}_{n}+\gamma \sum_{j=1}^{p}P_{st}\left(f_{j}\right)\leq |\beta |+3\sum_{j\in S}{\left\|f_{j}\right\|}_{n}$, *it holds that*

$$|\beta |/2+\sum_{j\in S}{\left\|f_{j}\right\|}_{n}\leq \|f\|\sqrt{\left|S\right|}/\phi \left(S\right),$$

*for some norm* ∥·∥.

**Definition 10 (Margin Condition)** *The margin condition holds if there is strictly convex function* G *such that* G(0)=0 *and for all* $f\in {ℱ}_{\text{local}}^{0}\subset {ℱ}^{0}$ *we have*

$$ℰ(f)\geq G\left(\left\|f-f^{0}\right\|\right),$$

*for some norm* ∥·∥ *on* ${ℱ}_{0}$; *here* ${ℱ}_{\text{local}}^{0}$ *is a neighborhood of* $f^{0}$ (*for example*, ${ℱ}_{\text{local}}^{0}=\{f:{\left\|f-f^{0}\right\|}_{\infty }\leq \eta \}$). *In typical cases*, *the margin condition holds with* $G(u)=cu^{2}$, *for a positive constant c*. *We refer to this special case as the quadratic margin condition*.

The norm ∥·∥ used in the definitions above is most often the empirical norm, $∥\cdot {∥}_{n}$. Our proof is the same for any norm ∥·∥, as long as the same norm is used for both conditions. Note that the margin condition is strictly a condition on the loss function ℓ(·), implying that it is not dependent on the class, ℱ, or dimension, p. While the margin condition is established for well-known choices of ℓ(·) (see for example, van de Geer, 2016), in Appendix H, we present a framework for verifying the quadratic margin condition for loss functions of the form: $-\ell (f)=ay_{i}f(x_{i})+b(f(x_{i}))$. While the compatibility condition is difficult to prove, the *theoretical compatibility condition* (defined below) can be verified under suitable conditions. In Appendix H, we prove that (under mild conditions), the theoretical compatibility condition implies the original compatibility condition with high probability.

**Definition 11 (Theoretical Compatibility Condition)** *The theoretical compatibility condition is said to hold for an index set* S⊂{1,2,…,p}, *for a compatibility constant $\tilde{\phi }(S)$*, *if for some* η∈(0,1/5), *all* λ>0, *and all functions of the form* $f(x)=\beta +\sum_{j=1}^{p}f_{j}\left(x_{j}\right)$ *that satisfy* $\sum_{j\in S^{c}}\left\|f_{j}\right\|+\frac{1-5\eta }{1-\eta }\sum_{j=1}^{p}\lambda P_{st}\left(f_{j}\right)\leq |\beta |+\frac{3(1+\eta )}{1-\eta }\sum_{j\in S}\left\|f_{j}\right\|$, *it holds that*

$$|\beta |+\sum_{j\in S}\left\|f_{j}\right\|\leq \frac{\sqrt{\left|S\right|}\|f\|}{\tilde{\phi }(S)},$$

*where* $∥f{∥}^{2}=\int [f(x){]}^{2}dQ(x)$ *is the population level* $L_{2}$ *norm*.

The theoretical compatibility condition holds trivially when we have independent covariates. In general, establishing verifying it depends on the smoothness penalty $P_{st}$; for example, for the Sobolev norm, Meier et al. (2009) established sufficient conditions for the compatibility condition to hold. An important special case, is when $P_{st}(f)$ projects component functions to a finite dimensional space (for example, Ravikumar et al., 2009; Lou et al., 2016). In this case, our condition reduces to the well-known, group lasso compatibility condition, for which sufficient conditions are well established in the literature (for example, Bühlmann and van de Geer, 2011).

We now present our second theorem which establishes the bound $ℰ\left(\hat{\beta }+\sum_{j=1}^{p}{\hat{f}}_{j}\right)≾s\lambda^{2},$, where λ is the slow rate of Theorem 7, and s is the number of non-zero components of $f^{*}=\beta +\sum_{j=1}^{p}f_{j}^{*}$, a sparse additive approximation of $f^{0}$. As in Theorem 7, the bound holds over a set 𝒯; Corollary 13 following the theorem shows that we lie in 𝒯 with high probability.

**Theorem 12 (Fast Rates for** GSAM**)** *Suppose* −ℓ(·) *and* $P_{st}$ *are convex functions and with* $\hat{f}$ *and* $f^{*}$ *as defined in Theorem 7*. *Assume that* $f^{*}$ *is sparse with* $|S_{*}|=s$ where $S_{*}=\left\{j:f_{j}^{*}\neq 0\right\}$, *and that the compatibility condition holds for* $S_{*}$. *Further assume the quadratic margin condition holds with constant c*, *and that for a function* $f(x)=\beta +\sum_{j=1}^{p}f_{j}\left(x_{j}\right)$, $f\in {ℱ}_{\text{local}}^{0}$ *if and only if* $|\beta -\beta^{*}|+I(f-f^{*})\leq M^{*}$. *The constant* $M^{*}$ *is defined as*

$$\rho M^{*}=ℰ\left(f^{*}\right)+\frac{16s\lambda^{2}}{c\phi^{2}\left(S_{*}\right)}+2\lambda^{2}\sum_{j\in S_{*}}P_{st}\left(f_{j}^{*}\right),$$

*and* ρ *is such that* λ≥8ρ. *Furthermore*, *define the set* 𝒯 *as*

$$𝒯=\left\{Z_{M^{*}}\leq \rho M^{*}\right\},\;\text{where}\;\;Z_{M^{*}}=\underset{\left|\beta -\beta^{*}\right|+I\left(f-f^{*}\right)\leq M^{*}}{\text{sup}}\left|\nu_{n}(f)-\nu_{n}\left(f^{*}\right)\right|.$$

*Then*, *on the set* 𝒯,

$$ℰ(\hat{f})+\lambda I\left(\hat{f}-f^{*}\right)\leq 4\rho M^{*}=4ℰ\left(f^{*}\right)+\frac{64s\lambda^{2}}{c\phi^{2}\left(S_{*}\right)}+8\lambda^{2}\sum_{j\in S_{*}}P_{st}\left(f_{j}^{*}\right).$$

**Corollary 13** *Let* $\hat{f}$ *and* $f^{*}$ *be as defined in Theorem 7 and assume the conditions of Theorem 12*. *Furthermore*, *for any function f assume the loss* ℓ(·) *is such that*

$$-\ell (f)=-\ell \left(y_{i},f\left(x_{i}\right)\right)=ay_{i}f\left(x_{i}\right)+b\left(f\left(x_{i}\right)\right),$$

*for some* a∈ℝ\{0} *and function* b:ℝ→ℝ. *Further assume that for* i=1,…,n, $y_{i}-Ey_{i}$ *are independent*, *uniformly sub-Gaussian:*

$$\underset{i=1,\ldots ,n}{\text{max}}K^{2}\left[E\;\text{exp}\left\{{\left(y_{i}-Ey_{i}\right)}^{2}/K^{2}\right\}-1\right]\leq \sigma_{0}^{2}.$$

*Finally suppose* $ℰ(f^{*})=O(s\lambda^{2}/\phi^{2}(S_{*}))$ *and* $s^{-1}\sum_{j\in S_{*}}P_{st}\left(f_{j}^{*}\right)=O(1)$. *Then*, *with probability at-least* $1–2\text{ exp}(-C_{1}n\rho^{2})–C\text{ exp}(-C_{2}n\rho^{2})$, *we have the following cases:*

1. *If* ℱ *has a logarithmic entropy bound*, *for* $\lambda ≍\rho ≍\kappa \;\text{max}\;\left(\sqrt{\frac{T_{n}}{n}},\sqrt{\frac{\text{log}\;p}{n}}\right),$,
$$ℰ(\hat{f})+\lambda I\left(\hat{f}-f^{*}\right)≾\text{max}\;\left(s\frac{T_{n}}{n},s\frac{\text{log}p}{n}\right),$$
  *with constants* $\kappa =\kappa (a,K,\sigma_{0},A_{0})$, $C_{1}=C_{1}(K,\sigma_{0})$, $C=C(K,\sigma_{0})$ *and* $C_{2}=C_{2}(C,\kappa )$.
2. *If* ℱ *has a polynomial entropy bound with smoothness*, *then for* $\lambda ≍\rho ≍\kappa \;\text{max}\;\left(n^{-\frac{1}{2+\alpha }},\sqrt{\frac{\text{log}\;p}{n}}\right)$,
(17) $$ℰ(\hat{f})+\lambda I\left(\hat{f}-f^{*}\right)≾\text{max}\;\left(sn^{-\frac{2}{2+\alpha }},s\frac{\text{log}\;p}{n}\right),$$
  *with constants* $\kappa =\kappa (a,K,\sigma_{0},A_{0},\alpha )$, $C_{1}=C_{1}(K,\sigma_{0})$, $C=C(K,\sigma_{0})$ *and* $C_{2}=C_{2}(C,\kappa )$.

We will discuss the significance of our theoretical results in the next subsection by specializing them to some well-studied special cases. Before discussing these specializations, we conclude this section by further generalizing Theorem 12. We will now assume a more general margin condition, for which we need to define the additional notion of a *convex conjugate*.

**Definition 14 (Convex Conjugate)** *Let* G *be a strictly convex function on* [0,∞) *with* G(0)=0. *The convex conjugate of* G, *denoted by* H, *is defined as*

$$H(v)=\underset{u}{\text{sup}\;}\{uv-G(u)\},v\geq 0.$$

*For the special case of* $G(u)=cu^{2}$, *one has* $H(v)=v^{2}/(4c)$.

**Theorem 15 (Fast Rates)** *Assume the conditions of Theorem 12 and define* $M^{*}$ *as*

$$\rho M^{*}=ℰ\left(f^{*}\right)+H\;\left(\frac{8\lambda \sqrt{s}}{\phi \left(S_{*}\right)}\right)+2\lambda^{2}\sum_{j\in S_{*}}P_{st}\left(f_{j}^{*}\right),$$

*where* H(·) *is the convex conjugate of* G. *Then*, *on the set* 𝒯,

$$ℰ(\hat{f})+\lambda I\left(\hat{f}-f^{*}\right)\leq 4\rho M^{*}.$$

**Remark 16 (Additional tuning parameters)** *Note that our convergence rates include the term* $\sum_{j\in S_{*}}P_{st}\left(f_{j}^{*}\right)$, *or constants which depend on it*. *For some choices of* $P_{st}$ *this can lead to poor finite sample performance*. *In such cases*, *prediction performance can be improved by solving instead*

(18) $$\hat{\beta },{\hat{f}}_{1},\ldots ,{\hat{f}}_{p}\leftarrow \underset{\beta \in \mathbb{R},f_{1},\ldots ,f_{p}\in ℱ}{\text{argmin}}-{\mathbb{P}}_{n}\ell \left(\beta +\sum_{j=1}^{p}f_{j}\right)+(1-\zeta )\lambda \sum_{j=1}^{p}P_{st}\left(f_{j}\right)+\zeta \lambda \sum_{j=1}^{p}{\left\|f_{j}\right\|}_{n},$$

*where* ζ∈[0,1] *is an additional tuning parameter*. *In theory*, *using two tuning parameters should lead to improved prediction*, *however in practice*, *tuning parameter selection over a discrete grid can become computationally cumbersome*. *A moderately-sized search grid might not yield a lower MSE and in fact*, *can lead to substantially higher MSE*, *particularly for large n*. *We illustrate this phenomenon via a small simulation study in* Appendix C: *to the simulation study of* Section 5, *specifically Scenarios 3 and 4*, *we additionally fit GSAMs by solving* (18). *Using a grid of ten* ζ *values*, *we observe improved prediction in some cases however*, *a ten-grid is too coarse to exhibit uniformly lower MSE*.

**Remark 17 (Constants in convergence rates)** *Our convergence rates are presented* up to constants. *To illustrate this fact*, *we consider the problem*

(19) $$\hat{\beta },{\hat{f}}_{1},\ldots ,{\hat{f}}_{p}\leftarrow \underset{\beta \in \mathbb{R},f_{1},\ldots ,f_{p}\in ℱ}{\text{argmin}}-{\mathbb{P}}_{n}\ell \left(\beta +\sum_{j=1}^{p}f_{j}\right)+\Theta \lambda^{2}\sum_{j=1}^{p}P_{st}\left(f_{j}\right)+\lambda \sum_{j=1}^{p}{\left\|f_{j}\right\|}_{n},$$

*for a constant* Θ *that does not depend on n or p*. *While* (19) *will have a different convergence rate than those presented in Theorems 7—15*, *the two rates will only differ by a constant that depends on* Θ. *Optimizing these constants is an interesting open problem that is beyond the scope of this manuscript*.

**Remark 18 (Convex indicator penalties)** *The above results do not directly extend to some convex indicator penalties*. *For some convex indicator penalties*, *such as $P_{st}(f)=I\left(f;\left\{f:f^{\prime }\geq 0\right\}\right)$*, *we require a third type of entropy condition:*

***Definition 19* (*Polynomial Entropy without Smoothness*)** *The univariate function class*, ℱ, *is said to have a polynomial entropy without smoothness bound if for all* j=1,…,p *we have*

$$H\;\left(\delta ,\left\{f_{j}\in ℱ:{\left\|f_{j}\right\|}_{n}+\gamma P_{st}\left(f_{j}\right)\leq 1\right\},\|\cdot {\|}_{Q_{j,n}}\right)\leq A_{0}\delta^{-\alpha },$$

*for some constant* $A_{0}$, *parameter* α∈(0,2) *and all* γ>0.

*Our results do not extend to convex indicator penalties because our proof relies on the fact that* $f_{j}-f_{j}^{*}\in ℱ\;\text{for}\;f_{j},f_{j}^{*}\in ℱ$ *; function classes with polynomial entropy without smoothness do not usually have this property*. *We defer the extension to convex indicator structural penalties to future work*.

**Remark 20 (Sub-Exponential residuals)** *In Corollaries 8 and 13 we can replace the requirement of uniformly sub-Gaussian residuals by the weaker condition of* uniformly sub-Exponential residuals. *To be precise*, *we would require*

$$\underset{i=1,\ldots ,n}{\text{max}}K^{2}\left[E\;\text{exp}{\left\{y_{i}-E\left(y_{i}\right)\right\}}^{2}/K^{2}-1-\left|y_{i}-E\left(y_{i}\right)\right|/K\right]\leq \sigma_{0}^{2}.$$

*However*, *sub-Exponential residuals would firstly require bounds for the* δ-*entropy with* bracketing. *The* δ-*entropy with bracketing is a stronger notion than* δ-*entropy without bracketing* (*the* δ-*entropy with bracketing is always larger than the* δ-*entropy without bracketing*). *Secondly*, *we would also require uniform bounds for each univariate function*, *specifically*, *we need*

$$\underset{j=1,\ldots ,p}{\text{max}}\underset{x}{\text{sup}\;}\left|f_{j}(x)/{\left\|f_{j}\right\|}_{n}\right|\leq R.$$

**Remark 21 (Comparison of rates to existing work)** *Here*, *we highlight the key differences between our theoretical result and existing work*. Koltchinskii and Yuan (2010) *and* Raskutti et al. (2012) *establish same rates of convergence as those in Corollaries 8 and 13*. *However*, *their work requires stronger assumptions*. *Both papers are restricted to the setting reproducing of kernel hilbert spaces* (*RKHS*) *and only allow an RKHS norm as the choice of smoothness penalty*. *They also assume known bounds on the additive functions or individual components*. *Additionally*, Raskutti et al. (2012) *assumes independence of covariates as opposed to a more general compatibility condition*. *The work of* Yuan and Zhou (2016), *extends the RKHS framework with results capturing the notion of* weak sparsity*; assuming bounds on the term* $\sum_{j=1}^{p}{\left\|f_{j}^{*}\right\|}_{n}^{q}\;\text{for}\;0\leq q<1$ *for* 0≤q<1 *they present rates* (*up to a constant*) *of the form*

(20) $$n^{-\frac{2}{2+\alpha }}+{\left(\frac{\text{log}\;p}{n}\right)}^{1-q/2}.$$

*Similarly*, Tan and Zhang (2019) *present rates of the form*

(21) $${\left(n^{-\frac{1}{2+\alpha (1-q)}}+\sqrt{\frac{\text{log}\;p}{n}}\right)}^{2-q}.$$

*Both* (20) *and* (21) *match our established rates for the limiting case of* q=0. *While* Tan and Zhang (2019) *relax some of the strong assumptions of* Yuan and Zhou (2016), *both papers deal exclusively with the least squares loss function*. *It is not clear if results like* (20) *and* (21) *can be established for general loss functions*.

Tan and Zhang (2019) *also present convergence rates in greater generality*, *namely*, *in terms of the integral of the entropy number*. *However the only special case they consider are function classes with polynomial entropy with smoothness*. *For this special case*, *their convergence rates match ours under a least squares loss; it is not clear if their results extend to GAMs nor is it clear what their rates are for other commonly used function classes*.

### 4.3 Special Cases of GSAM

In this subsection, we illustrate the main strength of our framework, namely its generalizability. We specialize our theoretical results to, various existing GSAMs, fully non-parametric regression, and also to (sparse-)GLMs. As per a reviewer’s suggestion, in Table A.1 in Appendix A, we summarize existing GSAMs and their limitations which our framework overcomes.

Firstly, the proposals of Ravikumar et al. (2009) and Lou et al. (2016), established convergence rates substantially slower than the minimax rates and only for the least squares loss. Our general framework, establishes the following convergence rates for both methods: $ℰ(\hat{f})≾\text{max}\;(sM/n,s\;\text{log}\;p/n)+ℰ\left(f^{*}\right)$, where M is the order of the basis expansion used for each ${\hat{f}}_{j}$. For an additive $f^{0}$, $ℰ(f^{*})$ is decreasing in M; and we require a value of M which balances the two terms in the rate. For function classes with polynomial entropy with smoothness, we recover rates (17) with $M≍n^{\frac{\alpha }{2+\alpha }}$. As noted in Section 4.2, the margin condition holds for a large class of loss functions; for both methods, the compatibility condition reduces to the well-studied, group lasso compatibility condition.

Next we consider the proposal of Meier et al. (2009) (see also Bühlmann and van de Geer, 2011; van de Geer, 2010); their theoretical results were limited to the least squares loss and the resulting convergence rate takes the form $ℰ(\hat{f})≾{\left(s\;\text{log}\;p/n\right)}^{2/(2+\alpha )}$. This rate is sub-optimal compared to our fast rate (17). Established rates for the diagnolized smoothness penalty of van de Geer (2010), were also sub-optimal and of the order $s(\text{log }p)n^{-2/(2+\alpha )}$. Our work bridges the following gaps in the theoretical work of Meier et al. (2009) and van de Geer (2010): (a) we establish minimax rates under identical compatibility conditions, (b) we extend their result beyond least squares loss functions and, (c) we establish *slow rates* under virtually no assumptions. As another example, we consider our own previous work (Haris et al., 2018), a GSAM which uses wavelet basis functions. Once the univariate problem (p=1) was solved for the wavelet bases, extending it to GSAM was trivially achieved using the results of this manuscript. The above examples demonstrate that not only do our theoretical results and proximal gradient descent algorithm improve existing results in the literature, but also, can be applied to fully develop any GSAM as long as we can solve the uni-variate problem of the form (12).

Next we show how some seemingly unrelated problems can also be treated as special cases of our framework. Firstly, we recover the special case of univariate nonparametric regression, that is, with p=1: the compatibility condition trivially holds leading to the usual rates $ℰ(\hat{f})≾n^{-2/(2+\alpha )}$. Next, we recover the multivariate nonparametric regression problem: suppose we have a single (but multivariate) component function $f_{1}:{\mathbb{R}}^{p}\to \mathbb{R}$. For various choices of $P_{st}$, the bound (16) holds with α=p/m for some smoothness parameter m. Again, the compatibility condition holds trivially, leading to the usual nonparametric rate $n^{-2m/(2m+p)}$. Finally, parametric regression models are also special cases of GSAM. Using a convex indicator for $P_{st}$, we can constrain each $f_{j}$ to be a linear function leading to GLMs. For low-dimensional GLMs, Corollary 13 gives the usual parametric rate, p/n. For high-dimensional GLMs, not only does our theorem recover the lasso rate, but our compatibility condition also matches that of lasso (Bühlmann and van de Geer, 2011).

## 5. Simulation Study

In this section, to complement our theoretical results, we conduct a simulation study to study the finite sample performance of various GSAMs as a function of n. The GSAMs we study are existing techniques in the literature obtained by various choices of the smoothness penalty, $P_{st}(\cdot )$. Our aim is to study the convergence of various methods with increasing n. For a more detailed simulation study we refer the reader to the original papers for each method (cited below). We consider the following choices for $P_{st}(\cdot )$:

1. **SpAM** (Ravikumar et al., 2009). $P_{st}(f)=I\left(f;\text{span}\left\{\psi_{1},\ldots ,\psi_{M}\right\}\right)$ for M∈{3,6,10,20,30,50,80}. We use the SAMR-package (Zhao et al., 2014).
2. **SSP** (Meier et al., 2009). $P_{st}(f)=\sqrt{\int_{x}{\left(f^{\left(2\right)}(x)\right)}^{2}dx}$, the Sobolev smoothness penalty (SSP). Given the lack of efficient software for this method, we implemented it using the algorithm and results of Section 3.
3. **TF** (Sadhanala and Tibshirani, 2019). $P_{st}(f)=\int_{x}|f^{\left(k+1\right)}(x)|dx$ for k∈{0,1,2}, trend filtering for additive models. We implemented this method using the algorithm of Section 3 where the univariate sub-problem (11) was solved using the R package glmgen (Arnold et al., 2014).

**Figure 1:**
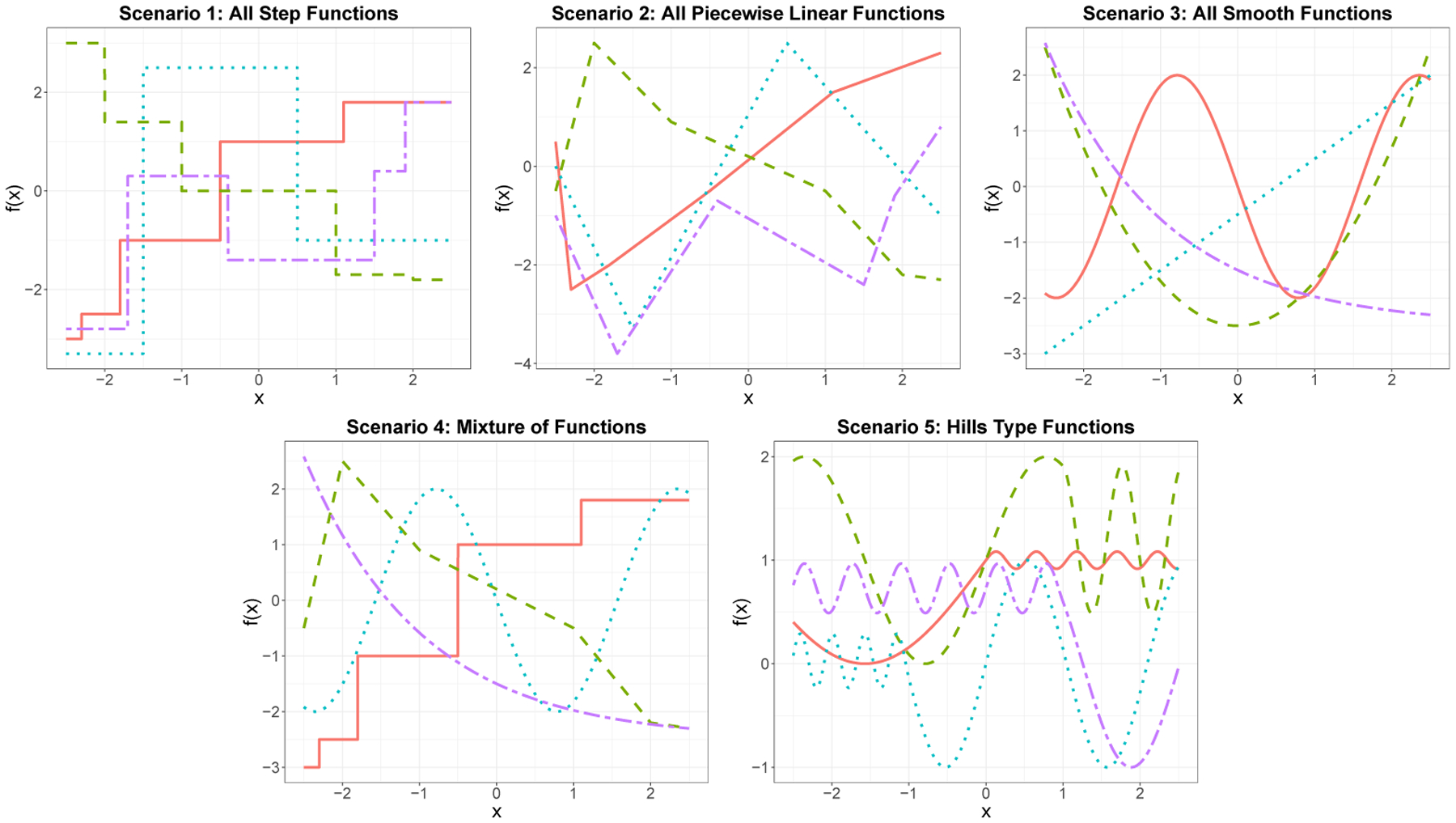
Plot of the 4 signal functions for each of the five simulation settings.

We simulate data for each of five simulation scenarios as follows: Given a sample size n and a number of covariates p, we draw 50 different n×p training design matrices X where each element is drawn from 𝒰(−2.5,2.5). We replicate each of the 50 design matrices 10 times leading to a total of 500 design matrices. The response is generated as $y_{i}=f_{1}(x_{i1})+f_{2}(x_{i2})+f_{3}(x_{i3})+f_{4}(x_{i4})+\varepsilon_{i}$ where $\varepsilon_{i}\sim 𝒩(0,1)$. The remaining covariates are noise variables. We also generate an independent test set for each replicate with sample size n/2. We vary the sample size, n∈{100,200,…,800} and consider both, a low-dimensional (p=6) and high-dimensional (p=100) settings. We consider five different choices of the signal functions as shown in Figure 1.

We fit each method over a sequence of 50 λ values on the training set, and select the tuning parameter $\lambda^{*}$ which minimizes the test error $\left({\left\|y_{\text{text}}-\hat{y}\right\|}_{n}^{2}\right)$. For the estimated model $\hat{f_{\lambda *}}$, we report the mean square error $\left(\text{MSE}\;;{\left\|{\hat{f}}_{\lambda^{*}}-f^{0}\right\|}_{n}^{2}\right)$ as a function of n.

**Figure 2:**
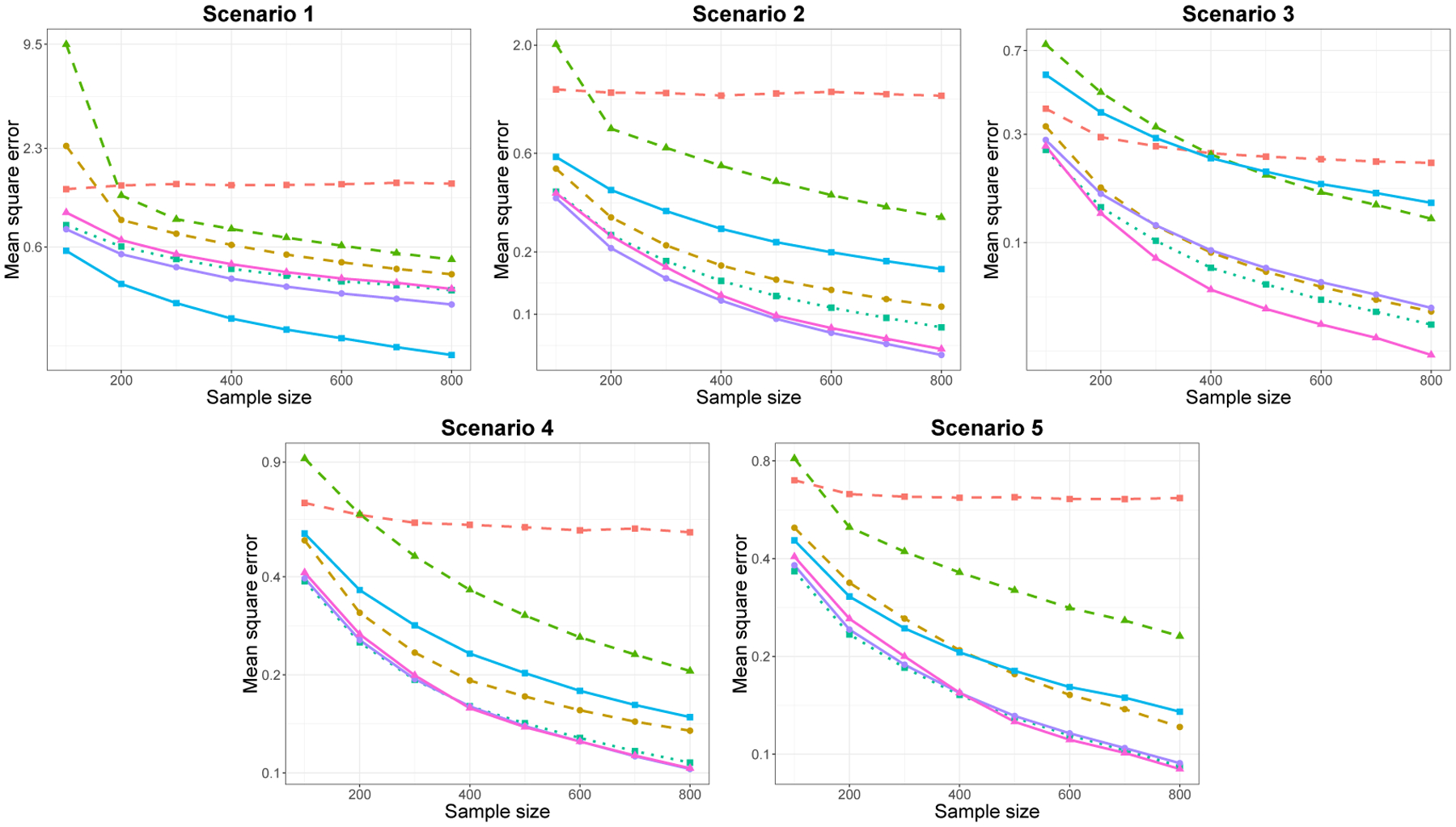
Plot of MSEs versus sample size for each of five scenarios for p=6, averaged over 500 replications. The dashed lines correspond to SpAM with small (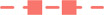), moderate (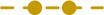) and high (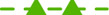) number of basis functions. The solid lines correspond to trend filtering of order k=0 (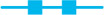), 1(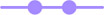) and 2 (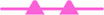). SSP is represented by the dotted line (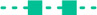).

**Figure 3:**
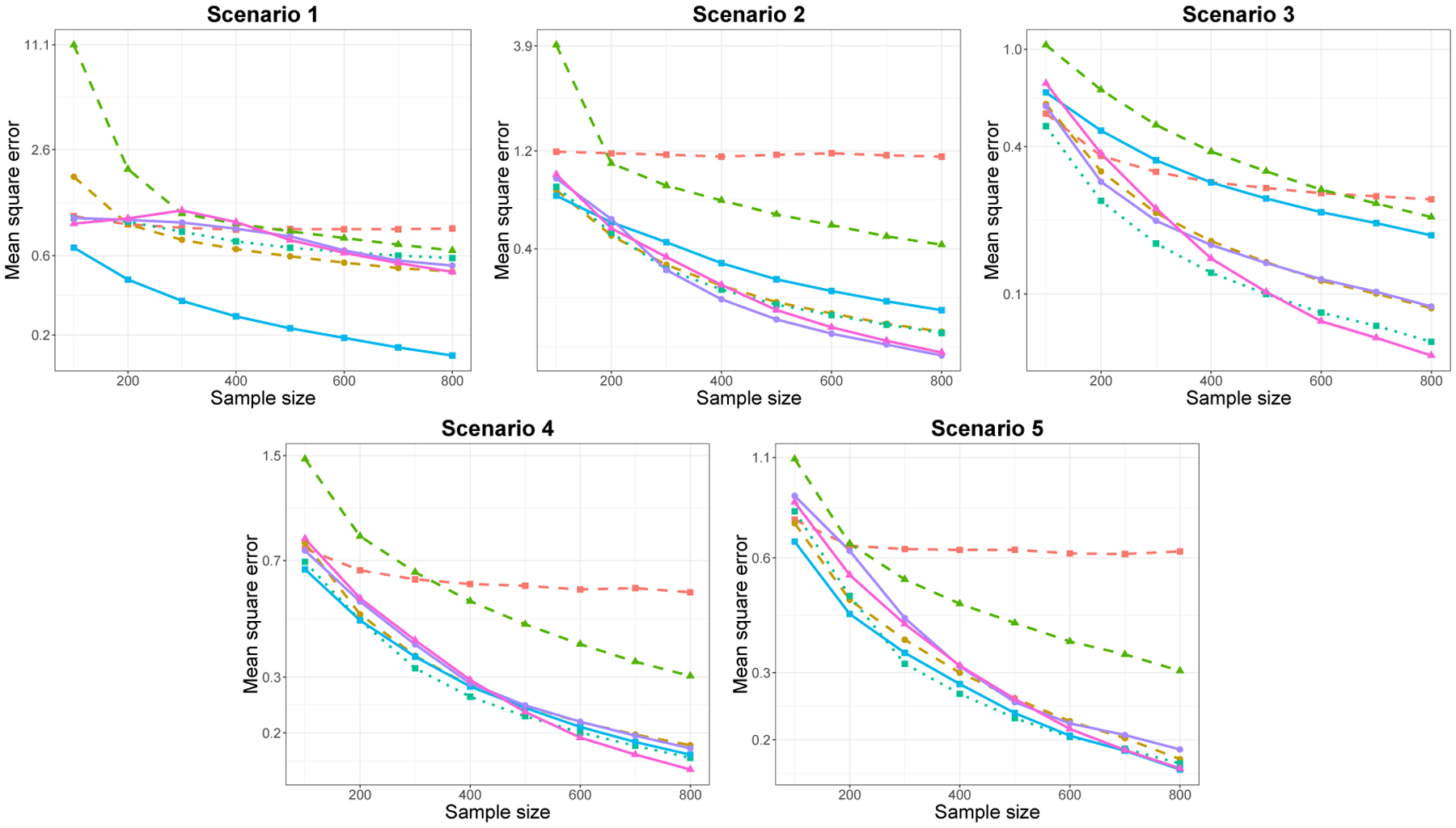
Plots of MSE versus sample size for each of five scenarios for p=100, averaged over 500 replications. The line types and colors are the same as in Figure 2.

In Figures 2 and 3, we plot the MSE as a function of n for the low and high-dimensional setting, respectively. For each simulation scenario, we plot the performance of SpAM for three different choices of M: low, moderate and high number of basis functions, M. The exact value of M presented varies by scenario, for example, in Scenario 4, low, moderate and high values of M correspond to M=3, 10 and 30, respectively. In both low- and high-dimensional settings, we observe similar relative performances between the methods, with more variability in results for the high-dimensional setting. While there is no uniformly superior method, for all, except Scenario 1, the Sobolev smoothness penalty and trend filtering of orders 1 and 2 had comparably good performances. Unsurprisingly, trend filtering of order 0 exhibits superior performance in Scenario 1, where each component is piecewise constant. In each scenario, the bias-variance trade-off of SpAM depends on the choice of M: too small or large values of M lead to high prediction error compared to other methods.

**Figure A.1:**
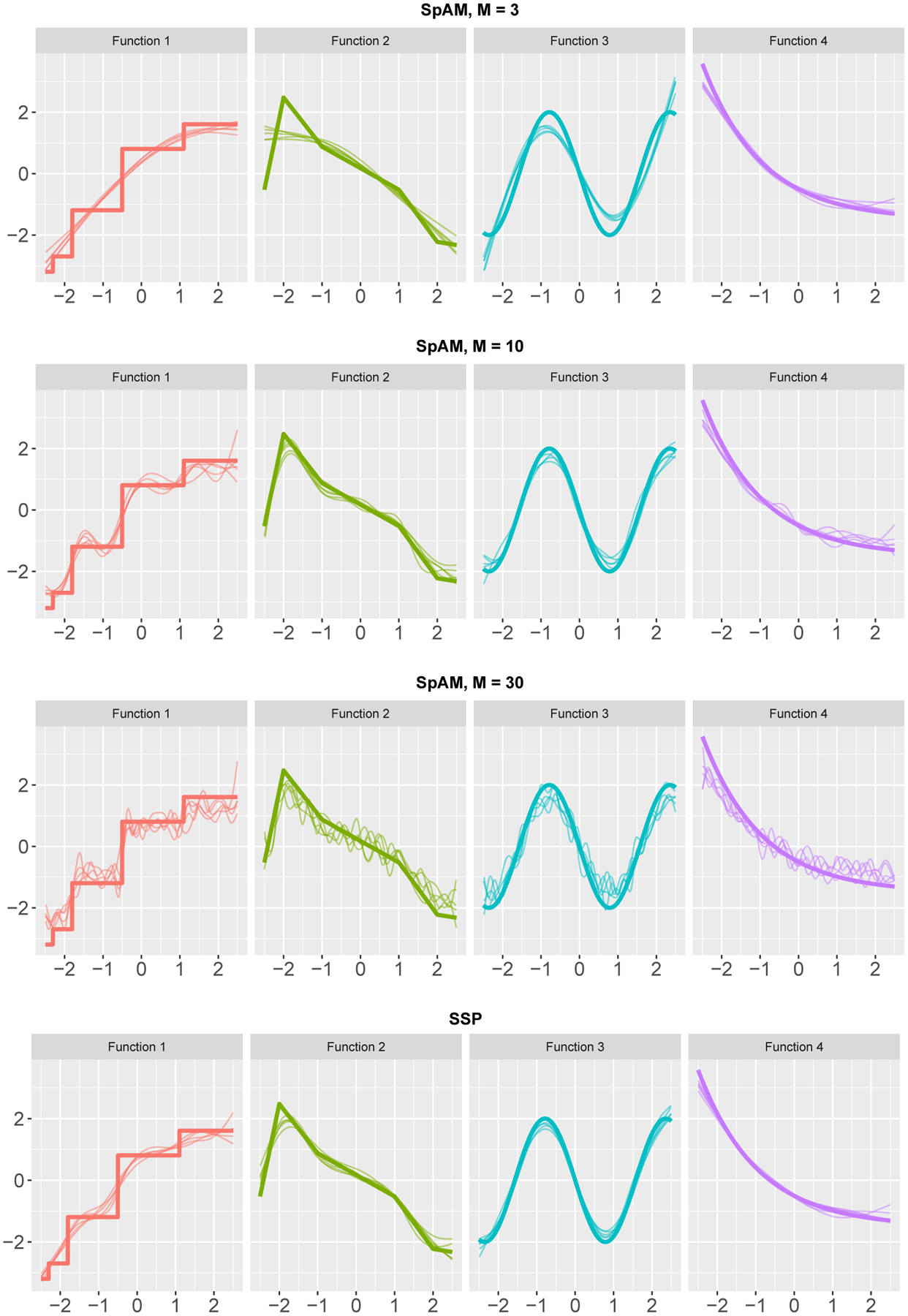
Examples of estimated signal functions by SpAM (Ravikumar et al., 2009) and Sobolev Smoothness Penalty (Meier et al., 2009) for Scenario 4.

**Figure A.2:**
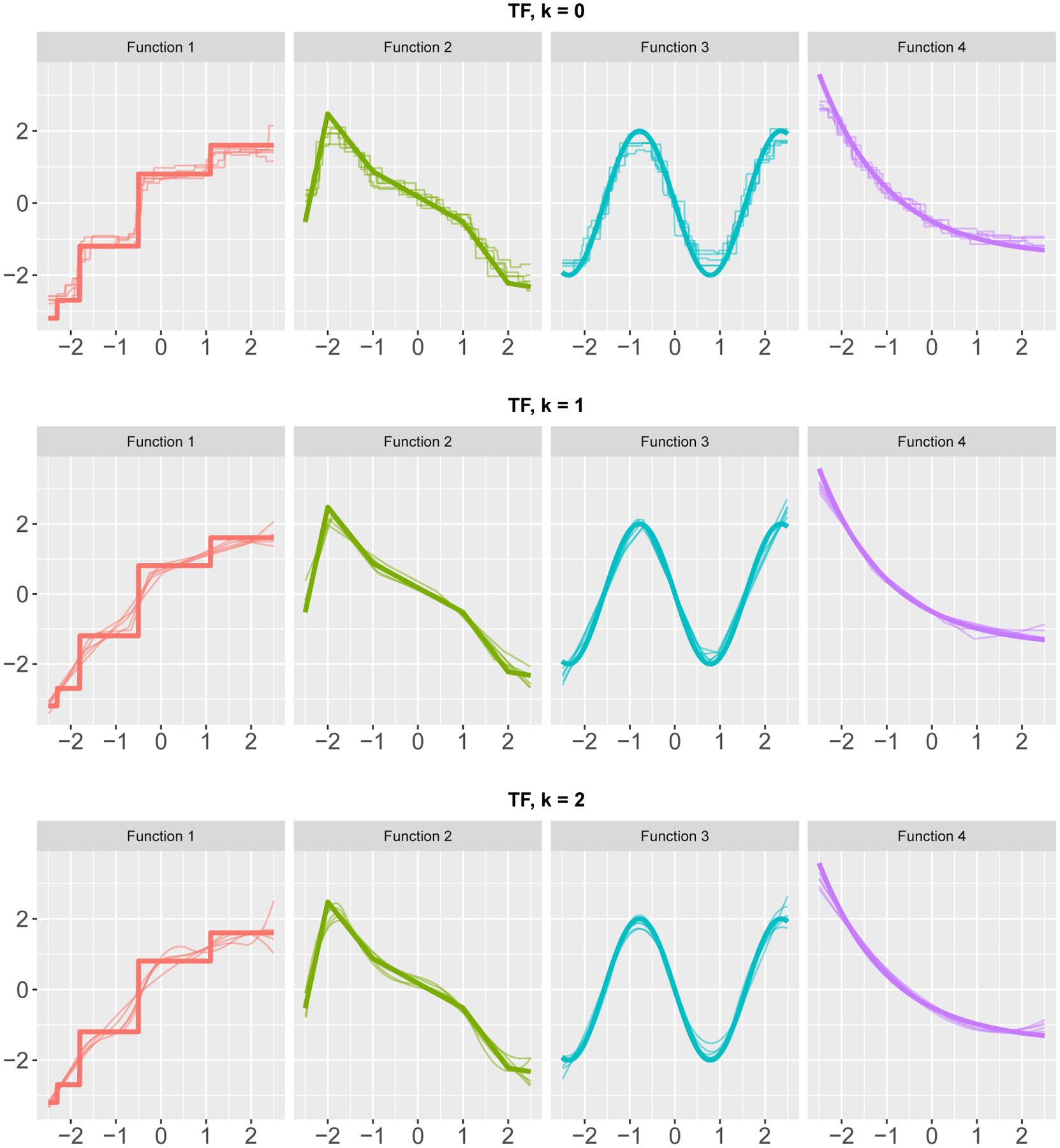
Examples of estimated signal functions by Trend Filtering (Sadhanala and Tibshirani, 2019) for Scenario 4.

In Appendix A, we plot examples of fitted functions for the various methods. The dependence on M for SpAM, is further illustrated in Figure A.1, where we plot functions estimated by SpAM for high-dimensional Scenario 4 with n=500. We observe large bias for M=3 (especially for the piecewise constant and linear functions) and high variance for M=30. In the same figure, we also plot functions estimated by the SSP; SSP estimates exhibit a similar bias to that of SpAM with M=10, but with a substantially smaller variance. Figure A.2 similarly plots fitted example functions for trend filtering. Trend filtering with k=0 estimates the piecewise constant function well, but estimating the other $f_{j}$’s by piecewise constant functions incurs additional variance. Trend filtering with k=1 and 2 estimates all other signal functions well.

## 6. Data Analysis

### 6.1 Boston Housing Data

We use the methods of Section 5 to predict the value of owner-occupied homes in the suburbs of Boston using census data from 1970. The data consists of n=506 measurements and 10 covariates, and has been studied in the additive models literature (Ravikumar et al., 2009; Lin and Zhang, 2006). As done in the data analysis by Ravikumar et al. (2009), we add 10 noise covariates uniformly generated on the unit interval and 10 additional noise covariates obtained by randomly permuting the original covariates.

We fit SSP, SpAM with M=2 and 3 basis functions, and TF with orders k=0,1,2; we also fit the lasso (Tibshirani, 1996). Approximately 75% of the observations are used as training set, and the mean square prediction error on the test set is reported. The final model is selected using 5-fold cross validation using the ‘1 standard error rule’. Results are presented for 100 splits of the data into training and test sets.

**Figure 4:**
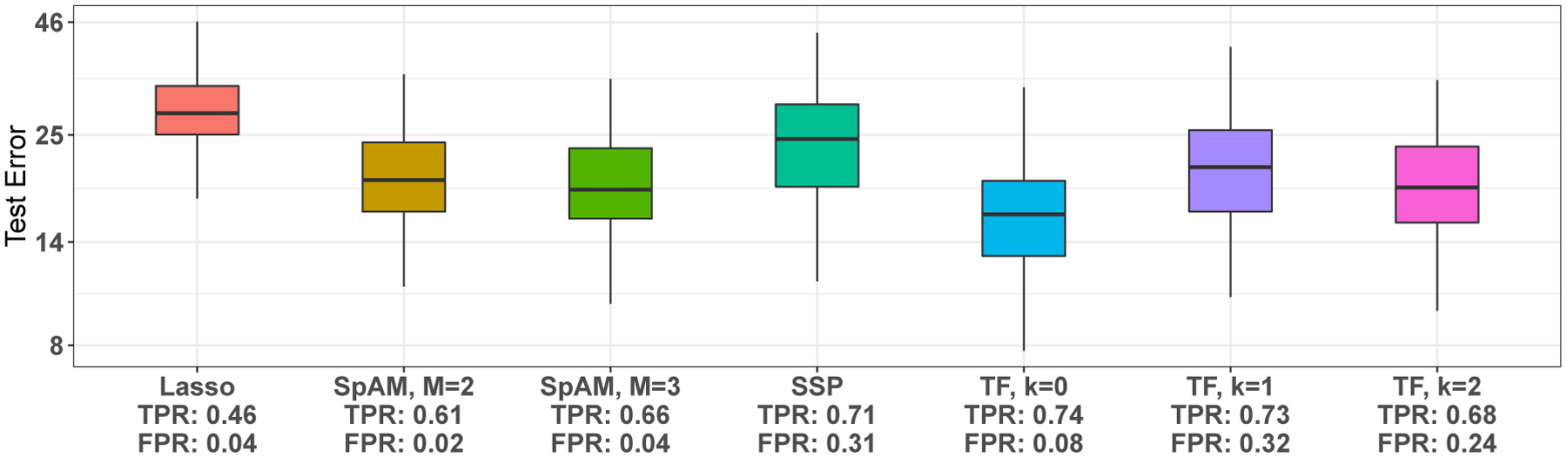
Box-plot of test errors for 100 different train/test splits of the data for each method. The average TPR and FPR was calculated using the original 10 covariates as ‘signal’ variables and remaining 20 as noise variables.

**Figure A.3:**
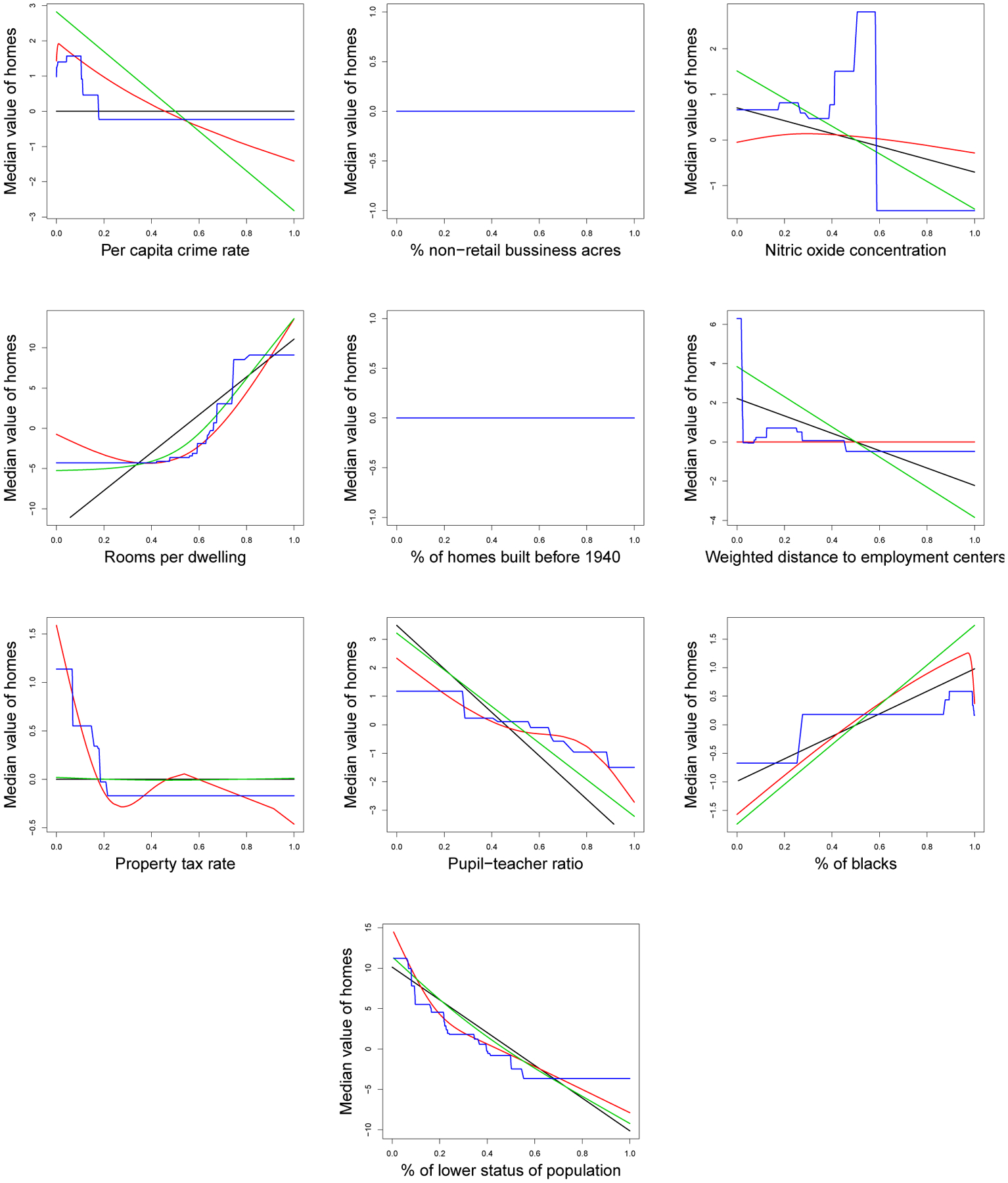
Plots of fitted functions for the original 10 covariates for a single split of the data into training and test sets for lasso (—), SpAM (—) with M=3 basis functions, SSP (—) and, TF (—) of order k=0.

The box-plots of test error in the test set are shown in Figure 4. Since we added noise variables for the purpose of this analysis, we also state the average true positive rate (TPR) and false positive rate (FPR) in Figure 4. The box-plots demonstrate superior performance of TF of order k=0 over other methods in terms of lowest prediction error and highest TPR. The FPR of TF with k=0 is also low (under 10%). In Figure A.3 of Appendix A, we plot fitted functions for one split of the data for lasso, SpAM with M=3, SSP and, TF with k=0 for the 10 covariates of the original data set. A striking feature of TF fits is that many component functions are constant for extreme values of the covariates.

### 6.2 Gene Expression Data

We now fit GSAMs for classification of gene expression data. We used the Curated Microarray Database (CuMiDa) (Feltes et al., 2019): a repository of gene-expression data sets curated from the Gene Expression Omnibus (GEO). Using gene expression measurements, we aimed to classify observations as either cancer cells or normal cells. We consider the following data sets/cancer types:

1. *Lung*: 54,675 gene expression measurements from 114 lung tissue samples from nonsmoking women with non-small cell lung carcinoma; data set consists of 56 tumor, and 58 normal tissue samples. Available on CuMiDa with accession number GSE19804.
2. *Prostate*: 12,620 gene expression measurements from 124 prostate tissue samples; data set consists of 64 primary prostate tumor, and 60 normal tissue samples. Available on CuMiDa with accession number GSE6919_U95B.
3. *Breast*: 35,980 gene expression measurements from 289 breast tissue samples; data set consists of 143 breast adenocarcinoma, and 143 normal tissue samples. Available on CuMiDa with accession number GSE70947.
4. *Oral cavity*: 54,675 gene expression measurements from 103 mucosa cell samples; data set consists of 74 samples with oral cavity cancer, and 29 normal cell samples. Available on CuMiDa with accession number GSE42743.

Our goal is to correctly classify samples as either normal or cancer samples. We split the data as follows: 60% as training, 20% as validation and 20% as test data. On the training data we fit the lasso, SpAM with M∈{2,3,10}, SSP and, TF with k∈{0,1}. TF with k=2 was excluded due to numerical instability of the current implementation of the algorithm in glmgen; SpAM with other values of M yielded similar performance and thus the results are omitted here. All methods were fit for a sequence of λ values, using $\left(\lambda_{sp},\lambda_{st})=(\lambda ,\lambda^{2}\right)$ for GSAMs. The λ value with the smallest area under the curve (AUC) for the ROC curve on the validation set was selected, and the corresponding model was used to classify samples in the test set. The experiment was repeated for 50 splits of the data into training, validation and testing.

In Table 1, we report the mean AUC on the test set and the estimated standard error based on 50 replications of the experiment. For the lung and breast cancer data sets, we observe similar performance between the lasso and other additive models; this suggests a low signal in the data to detect non-linearities. However, for prostate and oral cavity cancer, we observe a substantial gain (mean AUC beyond one SE) when using a GSAM instead of a linear model. In summary, this data analysis validates our intuition and theoretical results: using a GSAM will lead to comparable or better performance than using a linear model.

## 7. Conclusion

In this paper, we introduced a general framework for non-parametric high-dimensional sparse additive models. We show that many existing proposals, such as SpAM (Ravikumar et al., 2009), SPLAM (Lou et al., 2016), Sobolev smoothness (Meier et al., 2009), and trend filtering additive models (Sadhanala and Tibshirani, 2019; Petersen et al., 2016), fall within our framework.

We established a proximal gradient descent algorithm which has a lasso-like per-iteration complexity for certain choices of the structural penalty. The computational framework presented in this paper, effectively reduce the problem of fitting high-dimensional GSAMs to fitting a univariate regression model with the relevant smoothness penalty. While algorithms and theoretical results for specific GSAMs, as well as some theoretical results for certain types of general GSAMs, exists, to the best of our knowledge, the general algorithm for GSAMs is a key novel contribution in this paper. Our theoretical analyses in Section 4 showed both fast rates, which match minimax rates under Gaussian noise, as well as slow rates, which only require a few weak assumptions.

The R package GSAM, available on https://github.com/asadharis/GSAM, implements the methods described in this paper.

## Figures and Tables

**Table 1:** Table results for the analysis of gene expression data. For 100 different splits of the data into a training, testing and validation set, we report mean AUC along with $10^{3}\times$ mean SE, on the validation set. The method with the highest mean AUC is highlighted for each cancer type.

Method	Cancer type			
	*Lung*	*Prostate*	*Breast*	*Oral cavity*
	n=114; p=54,675	n=124; p=12,620	n=289; p=35,980	n=103; p=54,675
Lasso	0.985 (3.46)	0.713 (14.07)	0.951 (3.93)	0.930 (10.43)
SpAM, M=2	0.982 (3.49)	0.726 (13.56)	0.948 (3.89)	0.923 (14.14)
SpAM, M=3	0.984 (3.29)	0.763 (12.94)	**0.955** (3.50)	0.918 (13.06)
SpAM, M=10	0.970 (5.48)	0.727 (13.46)	0.946 (4.08)	0.940 (8.17)
SSP	0.987 (2.72)	0.765 (11.71)	0.934 (4.69)	0.950 (7.08)
TF, k=0	0.980 (4.07)	0.761 (13.04)	0.935 (4.66)	**0.953** (7.32)
TF, k=1	**0.988** (2.65)	**0.771** (12.50)	0.936 (4.22)	0.947 (9.14)

**Figure B.1:**
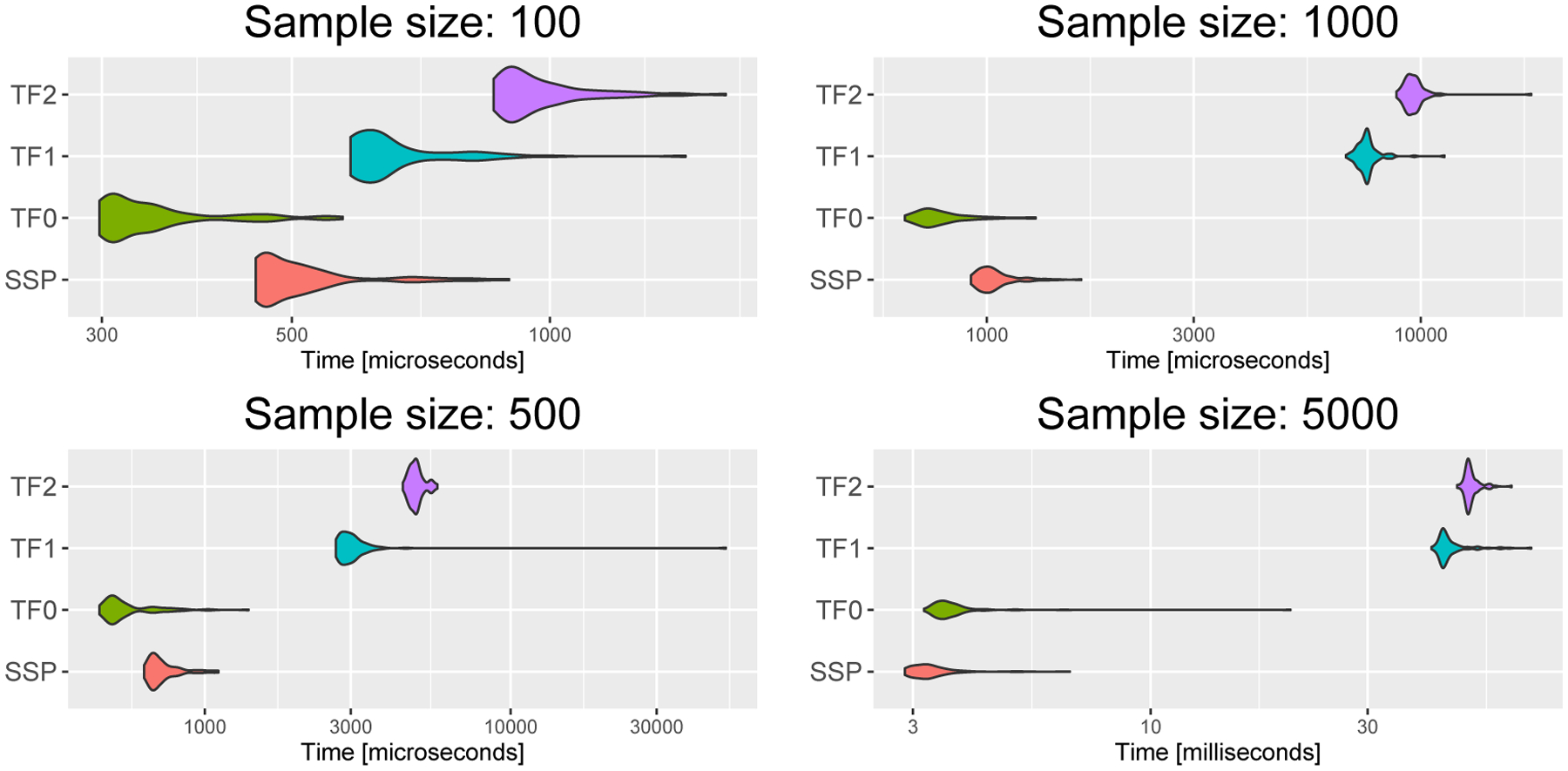
Timing results for 100 implementations of the proximal problem for SSP and trend filtering.

**Figure C.1:**
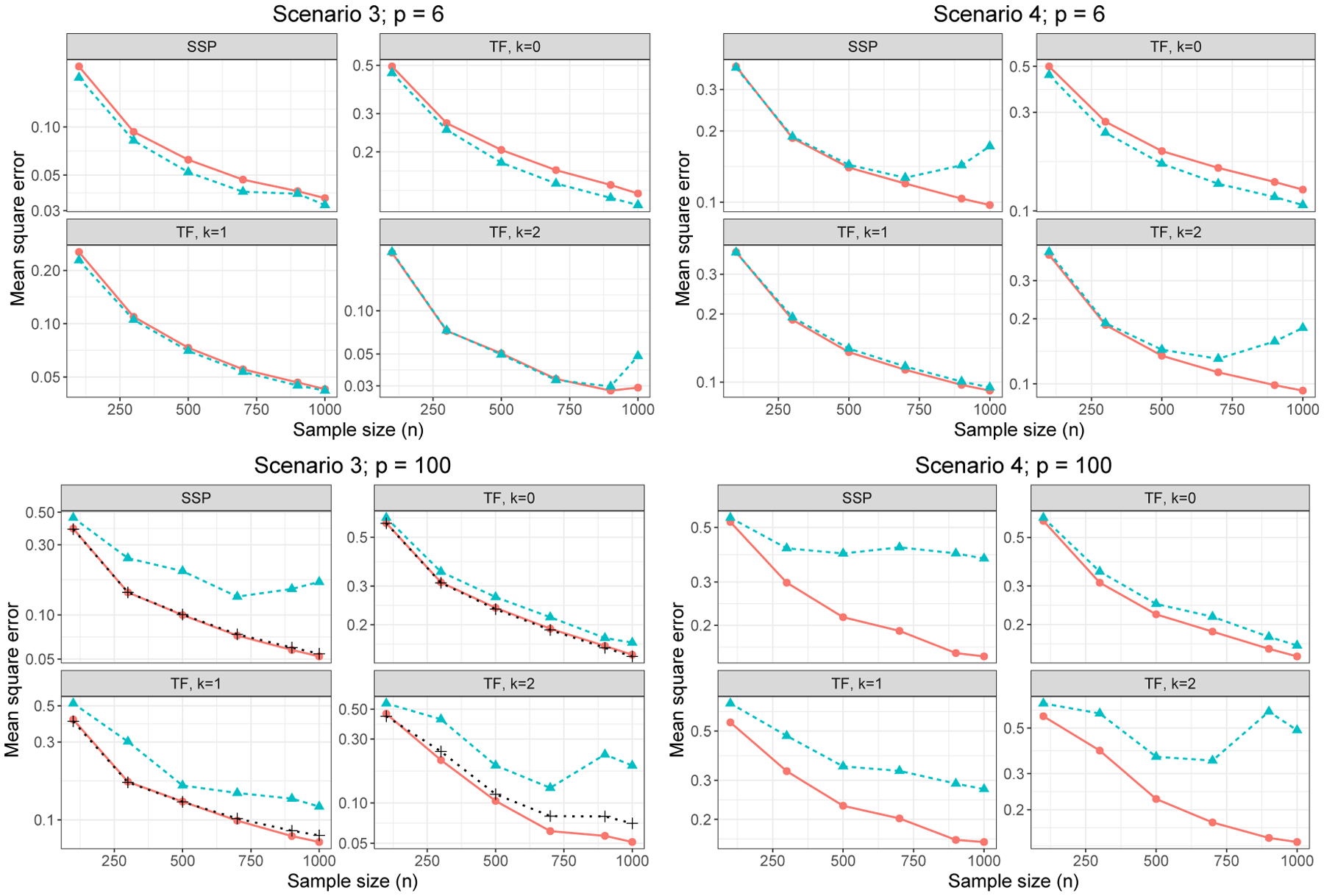
Plot of oracle MSEs versus sample size for each of scenarios 3 and 4 for p=6 and 100, averaged over 100 replications. The lines correspond to GSAM with coupled (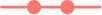) and decoupled (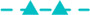) tuning parameters. For Scenario 3 with p=100, we also consider a finer grid of ζ values for decoupled tuning parameters (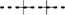).

## Appendix A. Additional Figures, Table and Algorithm

Algorithm A.1 is the block coordinate descent algorithm that can be used to estimate GSAMs.

Figure A.1 shows estimated functions for SpAM with 3,10 and 30 basis functions, and for SSP.

Figure A.2 shows estimated functions for trend filtering of orders k=0,1,2.

Figure A.3 shows estimated functions for the various methods for the analysis of Boston housing data.

Table A.1 summary of existing GSAMs in the literature and their shortcomings (lack of efficient algorithms and/or limited theoretical results).

**Table A.1:** Summary of methods generalized by our GSAM framework. The various methods in the literature are grouped according to (a) limited theoretical results (only for least squares or sub-optimal rates) and (b) absence of an efficient algorithm.

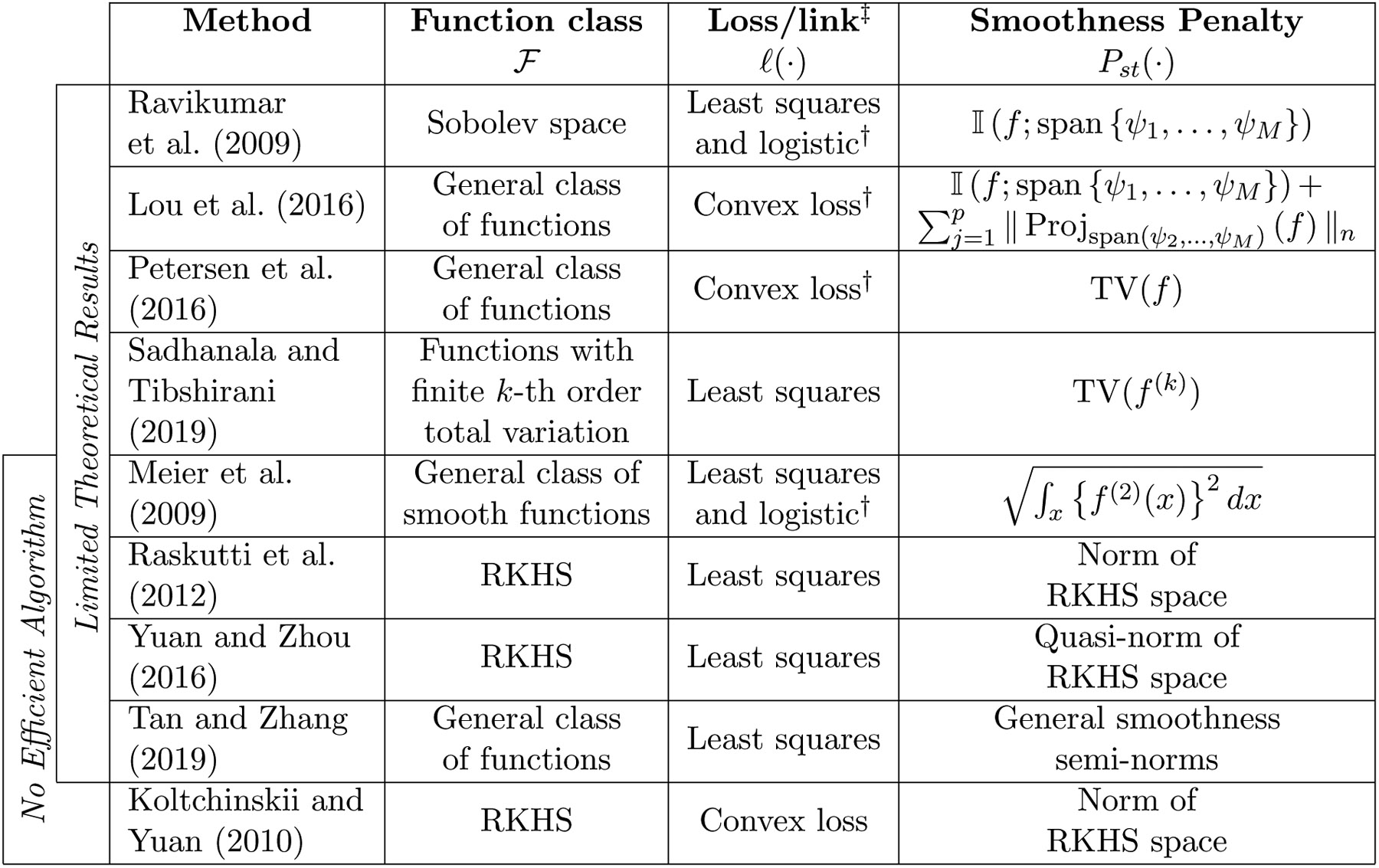

†: Theoretical results only available for least squares loss/identity link function.

‡: The link function is generally incorporated into the loss function, for example, least squares loss corresponds to the identity link and logistic loss to the logit link.

## Appendix B. Numerical Experiments for Comparing Run-Times

In this appendix, we present a detailed comparison of the run-times for solving the univariate proximal problem for various choices of the smoothness penalty, $P_{st}$. In greater detail, we study the proximal problem:

$$\underset{f\in ℱ}{\text{min}\;}\frac{1}{2}\|r-f{\|}_{n}^{2}+\lambda \|f{\|}_{n}+\lambda^{2}P_{st}(f).$$

We generated data as $x_{i}\sim \text{Unif}[-1,1]$ and $r_{i}=\text{sin}(1.5\pi x_{i})/2+\varepsilon_{i}$ where $\varepsilon_{i}\sim 𝒩(0,0.5^{2})$ for i=1,…,n. We considered sample sizes n=100, 500, 1000 and 5000.

In Table B.1 we present the timing results for repeatedly implementing the proximal solver 100 times for n=500. Unsurprisingly, SpAM is the fastest method as it can be viewed as a standardized group lasso problem (Simon and Tibshirani, 2012) where each proximal problem has a closed-form solution. The SSP penalty is slightly slower than trend filtering for order k=0 but still orders of magnitude faster than trend filtering for k=1,2.

In Figure B.1, we present violin plots for the timing results comparing SSP to trend filtering. This figure validates our previous observations: despite the added grid search, SSP is still much faster than trend filtering of order k=1,2. Another interesting feature is that SSP is the fastest method for n=5000.

While it is encouraging to see competitive computation time despite an added grid search, we must highlight one limitation of this experiment: each method was implemented based on existing code in R packages, and other publicly available sources. Therefore, there are bound to be differences in the efficacy of the code written including the choice of programming language used. For instance the SSP proximal problem is solved using FORTRAN, whereas others use C++; additionally, speed is impacted by other functions which might be written in R, for example, using for loops to construct the matrix of basis functions.

**Table B.1:** Summary of timing results for 100 implementations of proximal solver for n=500.

	Time (*μs*)					
	Min	Lower Quantile	Mean	Median	Upper Quantile	Max
SpAM, M=3	23.70	26.10	36.61	30.30	42.95	138.20
SpAM, M=5	25.30	28.90	40.15	32.90	42.20	143.60
SpAM, M=10	31.80	35.05	44.20	39.15	49.25	102.60
SpAM, M=15	38.00	42.95	55.48	46.85	56.90	202.00
SpAM, M=20	42.50	47.55	57.31	51.60	61.75	163.70
SpAM, M=30	55.00	61.00	75.79	69.25	77.10	248.20
SpAM, M=50	77.00	85.10	100.75	92.35	102.60	225.50
SSP	633.00	670.20	733.72	693.20	761.10	1107.80
TF, k=0	452.80	489.80	571.64	514.15	579.05	1390.20
TF, k=1	2681.40	2815.95	3495.12	2968.30	3134.55	50508.80
TF, k=2	4436.40	4710.05	4921.01	4884.90	5008.40	5759.70

## Appendix C. Numerical Experiments for Additional Tuning Parameters

In this appendix, we empirically investigate the impact of decoulping the tuning parameters for sparsity and smoothness. Recall our decoupled GSAM:

(C.1)
$$\hat{\beta },{\hat{f}}_{1},\ldots ,{\hat{f}}_{p}\leftarrow \underset{\beta \in \mathbb{R},f_{1},\ldots ,f_{p}\in ℱ}{\text{argmin}}-{\mathbb{P}}_{n}\ell \left(\beta +\sum_{j=1}^{p}f_{j}\right)+(1-\zeta )\lambda \sum_{j=1}^{p}P_{st}\left(f_{j}\right)+\zeta \lambda \sum_{j=1}^{p}{\left\|f_{j}\right\|}_{n},$$

for a second tuning parameter ζ∈[0,1]. To demonstrate the performance of decoupling tuning parameters, we implemented (C.1) using some of the data from our simulation study in Section 5. In greater detail, we consider Scenario 3 (all smooth functions) and Scenario 4 (mixture of functions) from the simulation study in Section 5. We generated data with n∈{100,300,500,700,900,1000} and p∈{6,100}. We implement both versions of GSAM, with the smoothness penalties, SSP and TF with k=0,1,2. For the originally proposed GSAM (with coupled tuning parameters), we use a sequence of fifty λ values and, for (C.1), we additionally used a sequence of ten $\zeta \in [10^{-3},1-10^{-5}]$ values (resulting in a 10 × 50 grid of tuning parameters). We report the *oracle* mean square error (MSE):

$${\text{MSE}}_{\text{coupled}}=\underset{\lambda }{\text{min}\;}{\left\|{\hat{f}}_{\lambda }-f^{0}\right\|}_{n}^{2},$$

$${\text{MSE}}_{\text{decoupled}}=\underset{\zeta ,\lambda }{\text{min}\;}{\left\|{\hat{f}}_{\zeta ,\lambda }-f^{0}\right\|}_{n}^{2}.$$

All results were averaged over 100 replications of the data.

In Figure C.1 we plot the oracle MSE as a function of the sample size. For low-dimensional data we observe that decoupling the tuning parameters can lead to a lower MSE in some cases. For example, in Scenario 3, with p=6 we observe the decoupled GSAM to have lower MSE for all methods for almost all sample sizes. However, even with p=6 we note decoupled tuning parameters lead to a high MSE for large n whereas coupled tuning parameters have a monotone decreasing MSE curve. This phenomenon is exacerbated for p=100, where we observe the coupled tuning parameters GSAM to uniformly beat the decoupled version in terms of oracle MSE. This behavior is likely due to the precision of our tuning parameters’ grid: using a finer grid of ζ values could lead to a lower MSE but at a high computational cost. We study the use of a finer grid for Scenario 3 with p=100: the third line in the panel is MSE_decoupled_ for a sequence of thirty ζ∈[0.700,0.999] values. We clearly observe a substantial reduction of oracle MSE from the original decoupled GSAM; additionally, for most methods, for some n, the decoupled GSAM outperforms the coupled GSAM.

## Appendix D. Proof of Results in Section 2.2

**Proof** [Proof of Lemma 1] For brevity, we write our optimization problem as

$$\hat{f_{1}},\ldots ,\hat{f_{p}}\leftarrow \underset{f_{1},\ldots ,f_{p}\in ℱ}{\text{argmin}}ℒ\left(f_{1},\ldots ,f_{p}\right),$$

where ℒ(·) is the objective function. The proof follows by contradiction: we assume some $\hat{f_{j}}\equiv 0$ while others are non-zero, and looking at the sub-gradient conditions we arrive at a contradiction.

In greater detail, assume without loss of generality, that for some k<p, ${\hat{f}}_{1},\ldots ,{\hat{f}}_{k}\neq 0$ and ${\hat{f}}_{k+1},\ldots ,{\hat{f}}_{p}=0$. Define the paths ${\hat{f}}_{j,\varepsilon_{j}}={\hat{f}}_{j}+\varepsilon_{j}h_{j}$ for any direction $h_{j}\in ℱ$ for j=1,…,p. The sub-gradient conditions state, that for any direction $h_{j}\in ℱ$, we must have

$${0\in \partial_{\varepsilon_{j}}ℒ\left({\hat{f}}_{1,\varepsilon_{1}},\ldots ,{\hat{f}}_{p,\varepsilon_{p}}\right)|}_{\varepsilon_{1}=\ldots =\varepsilon_{p}=0},$$

for all j∈{1,…,p}, where $\partial_{\varepsilon_{j}}$ denotes the sub-gradient set with respect to $\varepsilon_{j}$. For the non-zero functions, j∈{1,…,k}, the sub-gradient conditions are:

$$-{\langle y-{\hat{f}}_{1}-{\hat{f}}_{2}-\ldots -{\hat{f}}_{k},h_{j}\rangle }_{n}+\lambda_{1}\partial_{\varepsilon_{j}}P_{st}^{2}\left({\hat{f}}_{j,\varepsilon_{j}}\right)+\lambda_{2}\frac{{\langle {\hat{f}}_{j},h_{j}\rangle }_{n}}{{\left\|{\hat{f}}_{j}\right\|}_{n}}∋0,$$

where ${\langle a,b\rangle }_{n}=n^{-1}\sum_{i=1}^{n}a\left(x_{i}\right)b\left(x_{i}\right)$. Since the sub-gradient conditions must be met for all $h_{j}\in ℱ$, we set $h_{j}={\hat{f}}_{j}$. This implies that

$${\partial_{\varepsilon_{j}}P_{st}^{2}\left({\hat{f}}_{j,\varepsilon_{j}}\right)|}_{\varepsilon_{j}=0}={\partial_{\varepsilon_{j}}P_{st}^{2}\left\{\left(1+\varepsilon_{j}\right){\hat{f}}_{j}\right\}|}_{\varepsilon_{j}=0}\;={\partial_{\varepsilon_{j}}{\left(1+\varepsilon_{j}\right)}^{2}|}_{\varepsilon_{j}=0}P_{st}^{2}\left\{{\hat{f}}_{j}\right\}\;=2P_{st}^{2}\left({\hat{f}}_{j}\right),$$

where the second equality follows from properties of a norm. Thus, for j∈{1,…,k}, we must have

(D.1)
$$-{\langle y-{\hat{f}}_{1}-{\hat{f}}_{2}-\ldots -{\hat{f}}_{k},{\hat{f}}_{j}\rangle }_{n}+2\lambda_{1}P^{2}\left({\hat{f}}_{j}\right)+\lambda_{2}{\left\|{\hat{f}}_{j}\right\|}_{n}=0,\;\Leftrightarrow \frac{{\langle y-{\hat{f}}_{1}-{\hat{f}}_{2}-\ldots -{\hat{f}}_{k},{\hat{f}}_{j}\rangle }_{n}}{\lambda_{2}{\left\|{\hat{f}}_{j}\right\|}_{n}}=1+\frac{2\lambda_{1}P^{2}\left({\hat{f}}_{j}\right)}{\lambda_{2}{\left\|{\hat{f}}_{j}\right\|}_{n}}.$$

On the other hand for j′∈{k+1,…,p},

$${\partial_{\varepsilon_{j^{\prime }}}P_{st}^{2}\left({\hat{f}}_{j^{\prime },\varepsilon j^{\prime }}\right)|}_{\varepsilon_{j^{\prime }}=0}={\partial_{\varepsilon_{j^{\prime }}}P_{st}^{2}\left(\varepsilon_{j^{\prime }}h_{j^{\prime }}\right)|}_{\varepsilon_{j^{\prime }}=0}\;={\partial_{\varepsilon_{j^{\prime }}}\varepsilon_{j^{\prime }}^{2}|}_{\varepsilon_{j^{\prime }}=0}P_{st}^{2}\left(h_{j^{\prime }}\right)=0.$$

$$\partial_{\varepsilon_{j^{\prime }}}{{\left\|{\hat{f}}_{j^{\prime },\varepsilon_{j^{\prime }}}\right\|}_{n}|}_{\varepsilon_{j^{\prime }}=0}=\partial_{\varepsilon_{j^{\prime }}}{{\left\|\varepsilon_{j^{\prime }}h_{j^{\prime }}\right\|}_{n}|}_{\varepsilon_{j^{\prime }}=0}\;={\partial_{\varepsilon_{j^{\prime }}}\left|\varepsilon_{j^{\prime }}\right||}_{\varepsilon_{j^{\prime }}=0}{\left\|h_{j^{\prime }}\right\|}_{n},$$

By definition of a sub-gradient we know that ${\partial_{\varepsilon_{j^{\prime }}}\left|\varepsilon_{j^{\prime }}\right||}_{\varepsilon_{j^{\prime }}=0}=[-1,1]$ Therefore, the sub-gradient conditions for j′∈{k+1,…,p} imply that

(D.2)
$$-{\langle y-{\hat{f}}_{1}-{\hat{f}}_{2}-\ldots -{\hat{f}}_{k},h_{j^{\prime }}\rangle }_{n}+\lambda_{2}U_{j^{\prime }}h_{j^{\prime }n}=0,$$

for some $U_{j^{\prime }}\in [-1,1]$. Now with (D.1) and (D.2), and a clever choice of $h_{j^{\prime }}$ we arrive at a contradiction: setting $h_{j^{\prime }}=h_{j}={\hat{f}}_{j}\neq 0$ for any j≤k,

$$U_{j^{\prime }}=\frac{{\langle y-{\hat{f}}_{1}-{\hat{f}}_{2}-\ldots -{\hat{f}}_{k},{\hat{f}}_{j}\rangle }_{n}}{\lambda_{2}{\left\|{\hat{f}}_{j}\right\|}_{n}}\text{by}\;\text{(D}.2)\;=1+\frac{2\lambda_{1}P^{2}\left(\hat{f_{j}}\right)}{\lambda_{2}{\left\|{\hat{f}}_{j}\right\|}_{n}}\text{by}\;\text{(D}.1)\;>1,$$

because ${\hat{f}}_{j}\neq 0$, which leads to a contradiction. ■

**Proof** [Proof of Corollary 2] The proof is essentially identical to that of Lemma 1. Assume without loss of generality that I={k+1,…,p}. Assume for contradiction that, there is some j={1,…,k} such that $\hat{f_{j}}\in ℱ\backslash {ℱ}_{0}$. Then as in the proof of Lemma 1, we can arrive at a contradiction showing $U_{j^{\prime }}>1$ for all $j^{\prime }\in I$. ■

## Appendix E. Proof of Results in Section 3

**Proof** [Proof of Lemma 3] If $\tilde{f}=0$, then $\tilde{f}=0$ is trivially the solution to (8). Thus, throughout this proof, we consider $\tilde{f}\neq 0$.

**Case 1:** $∥\tilde{f}{∥}_{n}\geq \lambda_{2}$. In this case $c\hat{f}=\tilde{f}$ where $c={\left(1-\lambda_{2}/∥\tilde{f}{∥}_{n}\right)}^{-1}$. Let $f_{T}\in ℱ$ be some arbitrary function and define the function $h=f_{T}-\hat{f}$. We will show that along the path $\hat{f}+\varepsilon h$ for all ε∈[0,1], the objective

(E.1)
$$\frac{1}{2}∥r-(\hat{f}+\varepsilon h){∥}_{n}^{2}+\lambda_{1}P_{st}(\hat{f}+\varepsilon h)+\lambda_{2}∥\hat{f}+\varepsilon h{∥}_{n}$$

is minimized at ε=0. We begin by noting that

$$\frac{1}{2}∥r-(\tilde{f}+\varepsilon ch){∥}_{n}^{2}+\lambda_{1}P_{st}(\tilde{f}+\varepsilon ch),$$

is minimized at ε=0 because

$$\tilde{f}+\varepsilon ch=\tilde{f}+\varepsilon cf_{T}-\varepsilon c\hat{f}=(1-\varepsilon )\tilde{f}+\varepsilon cf_{T}\in ℱ,$$

for all ε∈[0,1] since ℱ is a convex cone. By the sub-gradient condition, we have

$$-{\langle r-\tilde{f},ch\rangle }_{n}+\lambda_{1}\vartheta_{1}=0,$$

for some ${\vartheta_{1}\in \partial P_{st}(\tilde{f}+\varepsilon ch)|}_{\varepsilon =0}$, or equivalently

$$c\left[-{\langle r-c\hat{f},h\rangle }_{n}+\lambda_{1}\vartheta_{2}\right]=0,$$

for some ${\vartheta_{2}\in \partial P_{st}(\hat{f}+\varepsilon h)|}_{\varepsilon =0}$.

At $\hat{f}+\varepsilon h$, one possible sub-gradient of the objective (E.1) is

$$-{\langle r-\hat{f},h\rangle }_{n}+\lambda_{1}\vartheta_{2}+\lambda_{2}\frac{{\langle \hat{f},h\rangle }_{n}}{∥\hat{f}{∥}_{n}}.$$

By the definition of c, we have that $\lambda_{2}/∥\hat{f}{∥}_{n}=c\lambda_{2}/∥\tilde{f}{∥}_{n}=c(1-1/c)=c-1$, and thus the above sub-gradient is

$$-{\langle r-\hat{f},h\rangle }_{n}+\lambda_{1}\vartheta_{2}+(c-1){\langle \hat{f},h\rangle }_{n}=-{\langle r-c\hat{f},h\rangle }_{n}+\lambda_{1}\vartheta_{2}=0.$$

Thus we have shown that the objective function (E.1) is minimized at ε=0. Since $f_{T}$ was an arbitrary function, we conclude that $\hat{f}$ is the solution of (8).

**Case 2:** $∥\tilde{f}{∥}_{n}<\lambda_{2}$. In this case we will show that $\hat{f}\equiv 0$. For this, we consider the path $\varepsilon f_{T}$ for ε∈[0,1] for an arbitrary $f_{T}\in ℱ$. We will show that the function

(E.2)
$$\frac{1}{2}{\left\|r-\varepsilon f_{T}\right\|}_{n}^{2}+\lambda_{1}P_{st}\left(\varepsilon f_{T}\right)+\lambda_{2}{\left\|\varepsilon f_{T}\right\|}_{n},$$

is minimized at ε=0 and since $f_{T}$ is arbitrary that will complete the proof.

As in the previous case, we begin by looking at the sub-gradient conditions for $\tilde{f}$. The expression

$$\frac{1}{2}{\left\|r-\left(\tilde{f}+\varepsilon f_{T}\right)\right\|}_{n}^{2}+\lambda_{1}P_{st}\left(\tilde{f}+\varepsilon f_{T}\right),$$

is minimized at ε=0 by definition of $\tilde{f}$. This leads us to the sub-gradient condition

$$-{\langle r-\tilde{f},f_{T}\rangle }_{n}+\lambda_{1}\vartheta_{1}=0\Leftrightarrow \vartheta_{1}=\frac{{\langle r-\tilde{f},f_{T}\rangle }_{n}}{\lambda_{1}}.$$

Now we describe the the sub-gradient conditions for (E.2). All sub-gradients of (E.2) at ε=0 are given by

(E.3)
$$-{\langle r,f_{T}\rangle }_{n}+\nu_{1}\lambda_{1}P_{st}\left(f_{T}\right)+\nu_{2}\lambda_{2}{\left\|f_{T}\right\|}_{n},$$

for real values $(\nu_{1},\nu_{2})\in [-1,1{]}^{2}$. To complete the proof we need to find $\nu_{1}$ and $\nu_{2}$ such that (E.3) is 0 and, $(\nu_{1},\nu_{2})\in [-1,1{]}^{2}$. Setting $\nu_{1}=\vartheta_{1}/P_{st}(f_{T})$ and $\nu_{2}=\langle \tilde{f},f_{T}\rangle /\left(\lambda_{2}{\left\|f_{T}\right\|}_{n}\right)$ clearly makes (E.3) 0 and so we need only prove that our choice of $\nu_{1}$ and $\nu_{2}$ lie within the interval [−1, 1].

Showing $|\nu_{1}|\leq 1$ is equivalent to $\left|\vartheta_{1}|\leq P_{st}(f_{T}\right)$. $\vartheta_{1}$ is a member of the sub-gradient set

$${\partial P_{st}\left(\tilde{f}+\varepsilon f_{T}\right)|}_{\varepsilon =0}=\left\{u\geq 0:P_{st}\left(\tilde{f}+\eta f_{T}\right)-P(\tilde{f})\geq u\times \eta \;\;\;\forall \eta \geq 0\right\}.$$

Thus $\vartheta_{1}$ must satisfy the inequality

$$\vartheta_{1}\eta \leq P_{st}\left(\tilde{f}+\eta f_{T}\right)-P_{st}(\tilde{f})\;\leq P_{st}(\tilde{f})+\eta P_{st}\left(f_{T}\right)-P_{st}(\tilde{f})=\eta P_{st}\left(f_{T}\right),$$

where the second inequality holds because $P_{st}$ is a semi-norm. This proves that $\left|\vartheta_{1}|\leq P_{st}(f_{T}\right)$.

Showing $|\nu_{2}|\leq 1$ is easier and follows by definition:

$$\left|\nu_{2}\right|=\frac{\left|\langle \tilde{f},f_{T}\rangle \right|}{\lambda_{2}{\left\|f_{T}\right\|}_{n}}\leq \frac{\|\tilde{f}{\|}_{n}{\left\|f_{T}\right\|}_{n}}{\lambda_{2}{\left\|f_{T}\right\|}_{n}}=\frac{\|\tilde{f}{\|}_{n}}{\lambda_{2}},$$

which is less than 1 since $\|\tilde{f}{\|}_{n}<\lambda_{2}$.

**Sufficient conditions for** $\hat{f_{j}}\equiv 0$: for the second part of this Lemma, we proceed from the sub-gradient condition (E.3). If we set $\nu_{1}=0$, then $\hat{f}\equiv 0$ if for every direction $f_{T}$ there exists $\nu_{2}\in [-1,1]$ such that

$$\nu_{2}\lambda_{2}{\left\|f_{T}\right\|}_{n}={\langle r,f_{T}\rangle }_{n}.$$

Which is equivalent to

(E.4)
$$\left|\frac{{\langle r,f_{T}\rangle }_{n}}{{\left\|f_{T}\right\|}_{n}}\right|\leq \lambda_{2}.$$

If $\lambda_{2}\geq ∥r{∥}_{n}$, then (E.4) is satisfied for all $f_{T}$ because by the Cauchy-Schwarz inequality

$$\left|\frac{{\langle r,f_{T}\rangle }_{n}}{{\left\|f_{T}\right\|}_{n}}\right|\leq \left|\frac{\|r{\|}_{n}{\left\|f_{T}\right\|}_{n}}{{\left\|f_{T}\right\|}_{n}}\right|=\|r{\|}_{n}\leq \lambda_{2}.$$

■

**Proof** [Prof of Lemma 4] Consider an arbitrary direction ${\hat{f}}_{\lambda }+\varepsilon h$ for some function h and ε in an open interval. We first consider the case $P_{st}\left({\hat{f}}_{\lambda }\right)\neq 0$. In this case if the directional derivative $\nabla_{h}P_{st}^{\nu }(\hat{f})$ exists then so does the directional derivative of $P_{st}(\hat{f})$ and is given by

$$\nabla_{h}P_{st}\left({\hat{f}}_{\lambda }\right)=\frac{\nabla_{h}P_{st}^{\nu }\left({\hat{f}}_{\lambda }\right)}{\nu P_{st}^{\nu -1}\left({\hat{f}}_{\lambda }\right)}.$$

This follows from standard arguments for derivative of power functions. Here we present the simple case of integer valued ν>1.

$$\nabla_{h}P_{st}\left({\hat{f}}_{\lambda }\right)=\underset{\varepsilon \to 0}{\text{lim}}\frac{P_{st}\left({\hat{f}}_{\lambda }+\varepsilon h\right)-P_{st}\left({\hat{f}}_{\lambda }\right)}{\varepsilon }\;=\underset{\varepsilon \to 0}{\text{lim}}\frac{P_{st}\left({\hat{f}}_{\lambda }+\varepsilon h\right)-P_{st}\left({\hat{f}}_{\lambda }\right)}{\varepsilon }\times \frac{\sum_{l=1}^{\nu }{\left\{P_{st}\left({\hat{f}}_{\lambda }+\varepsilon h\right)\right\}}^{\nu -l}{\left\{P_{st}\left({\hat{f}}_{\lambda }\right)\right\}}^{l-1}}{\sum_{l=1}^{\nu }{\left\{P_{st}\left({\hat{f}}_{\lambda }+\varepsilon h\right)\right\}}^{\nu -l}{\left\{P_{st}\left({\hat{f}}_{\lambda }\right)\right\}}^{l-1}}\;=\underset{\varepsilon \to 0}{\text{lim}}\frac{P_{st}^{\nu }\left({\hat{f}}_{\lambda }+\varepsilon h\right)-P_{st}^{\nu }\left({\hat{f}}_{\lambda }\right)}{\varepsilon }\times \underset{\varepsilon \to 0}{\text{lim}}\frac{1}{\sum_{l=1}^{\nu }{\left\{P_{st}\left({\hat{f}}_{\lambda }+\varepsilon h\right)\right\}}^{\nu -l}{\left\{P_{st}\left({\hat{f}}_{\lambda }\right)\right\}}^{l-1}}\;=\nabla_{h}P_{st}^{\nu }\left({\hat{f}}_{\lambda }\right)\times \frac{1}{\sum_{l=1}^{\nu }{\left\{P_{st}\left({\hat{f}}_{\lambda }\right)\right\}}^{\nu -l}}=\frac{\nabla_{h}P_{st}^{\nu }\left({\hat{f}}_{\lambda }\right)}{\nu P_{st}^{\nu -1}\left({\hat{f}}_{\lambda }\right)}.$$

Now, by the gradient condition, for $f_{\varepsilon }=\hat{f_{\lambda }}+\varepsilon h$,

(E.5)
$$\frac{\partial }{\partial \epsilon }{\left[\frac{1}{2}{\left\|r-f_{\varepsilon }\right\|}_{n}^{2}+\lambda P_{st}\left(f_{\varepsilon }\right)\right]}_{\epsilon =0}=-{\langle r-{\hat{f}}_{\lambda },h\rangle }_{n}+\lambda \frac{\nabla_{h}P_{st}^{\nu }\left({\hat{f}}_{\lambda }\right)}{\nu P_{st}^{\nu -1}\left({\hat{f}}_{\lambda }\right)}=0,$$

Similarly, for the path $f_{\tilde{\varepsilon }}={\tilde{f}}_{\tilde{\lambda }}+\tilde{\varepsilon }h$, by the gradient condition,

$$\frac{\partial }{\partial \tilde{\varepsilon }}{\left[\frac{1}{2}{\left\|r-f_{\tilde{\varepsilon }}\right\|}_{n}^{2}+\tilde{\lambda }P_{st}^{\nu }\left(f_{\tilde{\varepsilon }}\right)\right]}_{\tilde{\varepsilon }=0}=-{\langle r-{\tilde{f}}_{\tilde{\lambda }},h\rangle }_{n}+\tilde{\lambda }\nabla_{h}P_{st}^{\nu }\left({\tilde{f}}_{\tilde{\lambda }}\right)=0.$$

This is exactly the optimality condition (E.5) with $\tilde{\lambda }=\lambda {\left(\nu P_{st}^{\nu -1}\left({\hat{f}}_{\lambda }\right)\right)}^{-1}$. Thus, if $\nu \tilde{\lambda }P_{st}^{\nu -1}\left({\tilde{f}}_{\tilde{\lambda }}\right)=\lambda$ then ${\hat{f}}_{\lambda }={\tilde{f}}_{\tilde{\lambda }}$.

Now to show the case $P_{st}(\hat{f})=0$, we need to find conditions for which the objective

$$\frac{1}{2}{\left\|f_{\text{interp}}-f\right\|}_{n}^{2}+\lambda P_{st}(f)$$

is minimized by $\hat{f}=f_{\text{null}}$. We consider the functions $f_{h,\varepsilon }=(1-\varepsilon )f_{\text{null}}+\varepsilon h$ for ε∈[0,1] and show that for all h∈ℱ, the objective

(E.6)
$$\frac{1}{2}{\left\|f_{\text{interp}}-f_{h,\varepsilon }\right\|}_{n}^{2}+\lambda P_{st}\left(f_{h,\varepsilon }\right)$$

is minimized at ε=0, if and only if $\lambda \geq P_{st}^{*}\left(f_{\text{interp}}-f_{\text{null}}\right)$.

To see this, note that all subgradients of (E.6) at ε=0, are of the form

$${\langle f_{\text{interp}}-f_{\text{null}},f_{\text{null}}-h\rangle }_{n}+\lambda \kappa P_{st}(h),$$

for κ∈[−1,1]. For 0 to be a sub-gradient of (E.6) we need to have

$$\lambda \kappa P_{st}(h)={\langle f_{\text{interp}}-f_{\text{null}},h-f_{\text{null}}\rangle }_{n}.$$

Consequently, (E.6) is minimized at ε=0 if and only if for all h∈ℱ

$${\langle f_{\text{interp}}-f_{\text{null}},h-f_{\text{null}}\rangle }_{n}\leq \lambda P_{st}(h).$$

Using the decomposition $h=h_{0}+h_{⊥}\in {ℱ}_{1}⊕{ℱ}_{2}$, the above condition becomes

$$\frac{{\langle f_{\text{interp}}-f_{\text{null}},h_{0}-f_{\text{null}}\rangle }_{n}}{P_{st}\left(h_{⊥}\right)}+\frac{{\langle f_{\text{interp}}-f_{\text{null}},h_{⊥}\rangle }_{n}}{P_{st}\left(h_{⊥}\right)}\leq \lambda .$$

Now if $f_{\text{interp}}-f_{\text{null}}\in {ℱ}_{2}$, then the first part of the LHS is 0 and the second part is bounded above by $P_{st}^{*}\left(f_{\text{interp}}-f_{\text{null}}\right)$. To complete the proof we show that $f_{\text{interp}}-f_{\text{null}}$ is, infact, a member of ${ℱ}_{2}$. For this, it suffices to show that $\langle f_{\text{interp}}-f_{\text{null}},f_{\text{null}}-h_{\text{null}}\rangle_{n}=0$ for all $h_{\text{null}}\in {ℱ}_{1}$. We know that $f_{\text{null}}$ is the solution to the problem

$$\underset{f\in {ℱ}_{1}}{\text{minimize}\;}\frac{1}{2}{\left\|f_{\text{interp}}-f\right\|}_{n}^{2};$$

in other words, for all $h_{\text{null}}\in {ℱ}_{1}$, the expression

$$\frac{1}{2}{\left\|f_{\text{interp}}-(1-\varepsilon )f_{\text{null}}-\varepsilon h_{\text{null}}\right\|}_{n}^{2},$$

is minimized by ε=0. Equivalently (by the gradient condition), for all $h_{\text{null}}\in {ℱ}_{1}$,

$${\langle f_{\text{interp}}-f_{\text{null}},f_{\text{null}}-h_{\text{null}}\rangle }_{n}=0.$$

■

**Proof** [Proof of Convergence of Infinite-dimensional problem] We begin by re-writing our optimization problem as follows:

(E.7)
$$\hat{\beta },{\hat{\eta }}_{1},\ldots ,{\hat{\eta }}_{p}\leftarrow \underset{\beta \in \mathbb{R},\eta_{1},\ldots ,\eta_{p}\in {\mathbb{R}}^{n}}{\text{argmin}}-{\mathbb{P}}_{n}\ell \left(\beta +\sum_{j=1}^{p}\eta_{j}\right)+\lambda^{2}\sum_{j=1}^{p}P_{st}\left(\eta_{j}\right)+\lambda \sum_{j=1}^{p}{\left\|\eta_{j}\right\|}_{n},\;P_{st}\left(\eta_{j}\right)=\underset{f\in ℱ}{\text{min}}P_{st}(f)+I\left(\eta_{ji}=f\left(x_{ji}\right)\text{for}\;\text{all}i\right),$$

in which case, the term $P_{st}(\eta_{j})$, in (E.7), is a semi-norm over ${\mathbb{R}}^{n}$. We recover our estimate by

(E.8)
$${\hat{f}}_{j}\leftarrow \underset{f\in ℱ}{\text{argmin}}\;P_{st}(f)+I\left({\hat{\eta }}_{ji}=f\left(x_{ji}\right)\text{for}\;\text{all}i\right).$$

Thus a proximal gradient descent algorithm will solve the finite-dimensional problem (E.7) by standard convergence guarantees. We then just need to solve (E.8) for a given ${\hat{\eta }}_{j}$. Our Algorithm 1, does exactly that. To see this, re-write our main proximal problem, (11):

(E.9)
$$\eta_{j}^{\text{inter}}\leftarrow \underset{\eta \in {\mathbb{R}}^{n}}{\text{argmin}}\frac{1}{2}{\left\|\left(\eta_{j}^{k-1}-tr\right)-\eta \right\|}_{n}^{2}+t\lambda^{2}P_{st}(\eta ),$$

(E.10)
$$f_{j}^{\text{inter}}\leftarrow \underset{f\in ℱ}{\text{argmin}}\;P_{st}(f)+I\left(\eta_{ji}^{\text{inter}}=f\left(x_{ji}\right)\text{for}\;\text{all}i\right).$$

Thus, we see that (E.9) generates a proximal gradient descent algorithm which solves (E.7) and (E.10) is exactly the problem (E.8). This completes the proof. ■

## Appendix F. Proofs of Results in Section 4.2

In this section we present the proof Theorem 7 and 15 for the sake of completeness. The arguments presented here are only a slight modification to those of Bühlmann and van de Geer (2011) for proving LASSO rates. One notable difference is that we explicitly handle an unpenalized intercept term; another is our handling of the structural penalty, $P_{st}(\cdot )$. Throughout the proofs, we will use the so-called *basic inequalities*. Hence, for the sake of convenience, we state and prove these basic inequalities as a separate lemma.

**Lemma F.1 (Basic Inequality)** *Let* $\hat{f}(x)=\hat{\beta }+\sum_{j=1}^{p}{\hat{f}}_{j}\left(x_{j}\right)$ *be as defined in* (14), *and let* $f^{*}(x)=\beta^{*}+\sum_{j=1}^{p}f_{j}^{*}\left(x_{j}\right)$ *be an arbitrary additive function with* $\beta^{*}\in ℛ$ *and* $f_{j}^{*}\in ℱ$. *Then we have the following basic inequality*

$$ℰ\left(\hat{f}\right)+\lambda I\left(\hat{f}\right)\leq -\left[\nu_{n}\left(\hat{f}\right)-\nu_{n}\left(f^{*}\right)\right]+\lambda I\left(f^{*}\right)+ℰ\left(f^{*}\right).$$

*If we further assume that* −ℓ(·) *and* $P_{st}(\cdot )$ *are convex*, *then for all* t∈(0,1) *and* $\tilde{f}=t\hat{f}+\left(1-t\right)f^{*}$ *we have the following basic inequality*

$$ℰ\left(\tilde{f}\right)+\lambda I\left(\tilde{f}\right)\leq -\left[\nu_{n}\left(\tilde{f}\right)-\nu_{n}\left(f^{*}\right)\right]+\lambda I\left(f^{*}\right)+ℰ\left(f^{*}\right).$$

**Proof** For the first inequality, note that

$$-P_{n}\ell \left(\hat{f}\right)+\lambda I\left(\hat{f}\right)\leq -P_{n}\ell \left(f^{*}\right)+\lambda I\left(f^{*}\right),$$

which is equivalent to

$$\lambda I\left(\hat{f}\right)\leq P_{n}\ell \left(\hat{f}\right)-P_{n}\ell \left(f^{*}\right)+\lambda I\left(f^{*}\right)\;\Leftrightarrow P\left(\ell \left(f^{*}\right)\right)-P\left(\ell \left(\hat{f}\right)\right)+\lambda I\left(\hat{f}\right)\leq P_{n}\ell \left(\hat{f}\right)-P\left(\ell \left(\hat{f}\right)\right)-P_{n}\ell \left(f^{*}\right)+P\left(\ell \left(f^{*}\right)\right)+\lambda I\left(f^{*}\right)\;\Leftrightarrow P\left(\ell \left(f^{*}\right)\right)-P\left(\ell \left(\hat{f}\right)\right)+\lambda I\left(\hat{f}\right)\leq -\left[\left(P_{n}-P\right)\left(-\ell \left(\hat{f}\right)\right)-\left(P_{n}-P\right)\left(-\ell \left(f^{*}\right)\right)\right]+\lambda I\left(f^{*}\right)\;\Leftrightarrow P\left(\ell \left(f^{*}\right)\right)-P\left(\ell \left(\hat{f}\right)\right)+\lambda I\left(\hat{f}\right)\leq -\left[\nu_{n}\left(\hat{f}\right)-\nu_{n}\left(f^{*}\right)\right]+\lambda I\left(f^{*}\right)\;\Leftrightarrow P\left(\ell \left(f^{*}\right)\right)-P\left(\ell \left(f^{0}\right)\right)+P\left(\ell \left(f^{0}\right)\right)-P\left(\ell \left(\hat{f}\right)\right)+\lambda I(\hat{f})\leq -\left[\nu_{n}\left(\hat{f}\right)-\nu_{n}\left(f^{*}\right)\right]+\lambda I\left(f^{*}\right)\;\Leftrightarrow -ℰ\left(f^{*}\right)+ℰ\left(\hat{f}\right)+\lambda I\left(\hat{f}\right)\leq -\left[\nu_{n}\left(\hat{f}\right)-\nu_{n}\left(f^{*}\right)\right]+\lambda I\left(f^{*}\right)$$

For the second inequality we have by convexity

$$-P_{n}\ell \left(\tilde{f}\right)+\lambda I\left(\tilde{f}\right)\leq t\left[-P_{n}\ell \left(\hat{f}\right)+\lambda I\left(\hat{f}\right)\right]+\left(1-t\right)\left[-P_{n}\ell \left(f^{*}\right)+\lambda I\left(f^{*}\right)\right]\;\leq -P_{n}\ell \left(f^{*}\right)+\lambda I\left(f^{*}\right),$$

after which we simply need to repeat the arguments for the previous basic inequality with $\hat{f}$ replaced by $\tilde{f}$. ■

**Proof** [Proof of Theorem 7] Define

(F.1)
$$t=\frac{M^{*}}{M^{*}+I\left(\hat{f}-f^{*}\right)},$$

and $\tilde{f}=t\hat{f}+\left(1-t\right)f^{*}$. Then (F.1) implies $I\left(\tilde{f}-f^{*}\right)\leq M^{*}$ and by Lemma F.1, we obtain

$$ℰ\left(\tilde{f}\right)+\lambda I\left(\tilde{f}\right)\leq Z_{M^{*}}+\lambda I\left(f^{*}\right)+ℰ\left(f^{*}\right).$$

By applying the triangle inequality $I\left(\tilde{f}-f^{*}+f^{*}\right)\geq I\left(\tilde{f}-f^{*}\right)-I\left(f^{*}\right)$, to the left hand side we obtain on the set 𝒯 (where $Z_{M^{*}}\leq \rho M^{*}+2R\rho$)

$$ℰ\left(\tilde{f}\right)+\lambda I\left(\tilde{f}-f^{*}\right)\leq \rho M^{*}+2R\rho +2\lambda I\left(f^{*}\right)+ℰ\left(f^{*}\right).$$

Recall the definition of $M^{*}$, given by

$$\rho M^{*}=ℰ\left(f^{*}\right)+2\lambda I\left(f^{*}\right)+2R\rho ,$$

from which we obtain

$$ℰ\left(\tilde{f}\right)+\lambda I\left(\tilde{f}-f^{*}\right)\leq 2\rho M^{*}\leq 2\frac{\lambda }{4}M^{*}\Rightarrow I\left(\tilde{f}-f^{*}\right)\leq \frac{M^{*}}{2}.$$

Now by the definition of $\tilde{f}$ we have

$$I\left(\hat{f}-f^{*}\right)=\frac{I\left(\tilde{f}-f^{*}\right)}{t}=I\left(\tilde{f}-f^{*}\right)\left[1+\frac{I\left(\hat{f}-f^{*}\right)}{M^{*}}\right]\leq \frac{M^{*}}{2}+\frac{I\left(\hat{f}-f^{*}\right)}{2},$$

which implies that $I\left(\hat{f}-f^{*}\right)\leq M^{*}$. Now we can repeat the above arguments with $\tilde{f}$ replaced by $\hat{f}$ which gives us

$$ℰ(\hat{f})+\lambda I\left(\hat{f}-f^{*}\right)\leq \rho M^{*}+\rho (2R)+2\lambda I\left(f^{*}\right)+ℰ\left(f^{*}\right).$$

■

**Proof** [Proof of Theorem 15] As in the proof of Theorem 7, we begin by defining t as

$$t=\frac{M^{*}}{M^{*}+\left|\hat{\beta }-\beta^{*}\right|+I\left(\hat{f}-f^{*}\right)},$$

and $\tilde{f}=t\hat{f}+\left(1-t\right)f^{*}$ which gives us $\left|\tilde{\beta }-\beta^{*}\right|+I\left(\tilde{f}-f^{*}\right)\leq M^{*}$ implying that, $\tilde{f}\in {ℱ}_{\text{local}}^{0}$.

Lemma F.1 implies that on the set 𝒯 (where $Z_{M^{*}}\leq \rho_{M^{*}}$) we have

(F.2)
$$ℰ\left(\tilde{f}\right)+\lambda I\left(\tilde{f}\right)\leq Z_{M^{*}}+{ℰ}^{*}+\lambda I\left(f^{*}\right)\leq \rho M^{*}+ℰ\left(f^{*}\right)+\lambda I\left(f^{*}\right).$$

Be definition of $S_{*}$ and I(·), we have by the triangle inequality

$$\lambda I\left(f^{*}\right)=\lambda \sum_{j\in S_{*}}\left\{{\left\|f_{j}^{*}\right\|}_{n}+\lambda P_{st}\left(f_{j}^{*}\right)\right\}\leq \lambda \sum_{j\in S_{*}}\left\{{\left\|f_{j}^{*}-{\tilde{f}}_{j}\right\|}_{n}+{\left\|{\tilde{f}}_{j}\right\|}_{n}+\lambda P_{st}\left(f_{j}^{*}\right)\right\},$$

and by the reverse triangle inequality we have

$$\lambda I\left(\tilde{f}\right)=\lambda \sum_{j\in S_{*}}\left\{{\left\|{\tilde{f}}_{j}\right\|}_{n}+\lambda P_{st}\left({\tilde{f}}_{j}\right)\right\}+\lambda \sum_{j\in S_{*}^{c}}\left\{{\left\|{\tilde{f}}_{j}\right\|}_{n}+\lambda P_{st}\left({\tilde{f}}_{j}\right)\right\}\;\geq \lambda \sum_{j\in S_{*}}\left\{{\left\|{\tilde{f}}_{j}\right\|}_{n}+\lambda P_{st}\left({\tilde{f}}_{j}-f_{j}^{*}\right)-\lambda P_{st}\left(f_{j}^{*}\right)\right\}+\lambda \sum_{j\in S_{*}^{c}}\left\{{\left\|{\tilde{f}}_{j}\right\|}_{n}+\lambda P_{st}\left({\tilde{f}}_{j}\right)\right\}\;=\lambda \sum_{j\in S_{*}}\left\{{\left\|{\tilde{f}}_{j}\right\|}_{n}+\lambda P_{st}\left({\tilde{f}}_{j}-f_{j}^{*}\right)-\lambda P_{st}\left(f_{j}^{*}\right)\right\}+\lambda \sum_{j\in S_{*}^{c}}\left\{{\left\|{\tilde{f}}_{j}-f_{j}^{*}\right\|}_{n}+\lambda P_{st}\left({\tilde{f}}_{j}-f_{j}^{*}\right)\right\},$$

where the last equality follows from the fact that $f_{j}^{*}=0$ for all $j\in S_{*}^{c}$. With the above two inequalities combined with (F.2) we get

$$ℰ\left(\tilde{f}\right)+\lambda \sum_{j\in S_{*}}\left\{{\left\|{\tilde{f}}_{j}\right\|}_{n}+\lambda P_{st}\left({\tilde{f}}_{j}-f_{j}^{*}\right)-\lambda P_{st}\left(f_{j}^{*}\right)\right\}+\lambda \sum_{j\in S_{*}^{c}}\left\{{\left\|{\tilde{f}}_{j}\right\|}_{n}+\lambda P_{st}\left({\tilde{f}}_{j}\right)\right\}\leq \lambda \sum_{j\in S_{*}}\left\{{\left\|f_{j}^{*}-{\tilde{f}}_{j}\right\|}_{n}+{\left\|{\tilde{f}}_{j}\right\|}_{n}+\lambda P_{st}\left(f_{j}^{*}\right)\right\}+\rho M^{*}+ℰ\left(f^{*}\right),$$

which simplifies to

(F.3)
$$ℰ\left(\tilde{f}\right)+\lambda \sum_{j\in S_{*}^{c}}{\left\|{\tilde{f}}_{j}-f_{j}^{*}\right\|}_{n}+\lambda \sum_{j=1}^{p}\lambda P_{st}\left({\tilde{f}}_{j}-f_{j}^{*}\right)\leq \lambda \sum_{j\in S_{*}}{\left\|f_{j}^{*}-{\tilde{f}}_{j}\right\|}_{n}+2\lambda^{2}\sum_{j\in S_{*}}P_{st}\left(f_{j}^{*}\right)+\rho M^{*}+ℰ\left(f^{*}\right).$$

Now we add $\lambda \left|\tilde{\beta }-\beta^{*}\right|+\lambda \sum_{j\in S_{*}}{\left\|{\tilde{f}}_{j}-f_{j}^{*}\right\|}_{n}$ to both sides of (F.3) to obtain

(F.4)
$$ℰ\left(\tilde{f}\right)+\lambda \left\{\left|\tilde{\beta }-\beta^{*}\right|+I\left(\tilde{f}-f^{*}\right)\right\}\leq 2\lambda \sum_{j\in S_{*}}{\left\|f_{j}^{*}-{\tilde{f}}_{j}\right\|}_{n}+\lambda \left|\tilde{\beta }-\beta^{*}\right|+\rho M^{*}+ℰ\left(f^{*}\right)+2\lambda^{2}\sum_{j\in S_{*}}P_{st}\left(f_{j}^{*}\right).$$

**Case I**. If

$$2\lambda \sum_{j\in S_{*}}{\left\|f_{j}^{*}-{\tilde{f}}_{j}\right\|}_{n}+\lambda \left|\tilde{\beta }-\beta^{*}\right|\leq \rho M^{*}+ℰ\left(f^{*}\right)+2\lambda^{2}\sum_{j\in S_{*}}P_{st}\left(f_{j}^{*}\right),$$

then (F.4) simplifies to

$$ℰ\left(\tilde{f}\right)+\lambda \left\{|\tilde{\beta }-\beta^{*}|+I\left(\tilde{f}-f^{*}\right)\right\}\leq 2\rho M^{*}+2ℰ\left(f^{*}\right)+4\lambda^{2}\sum_{j\in S_{*}}P_{st}\left(f_{j}^{*}\right)\leq 4\rho M^{*}\leq 4\frac{\lambda }{8}M^{*}=\lambda M^{*}/2,$$

which indicates that $\left|\tilde{\beta }-\beta^{*}\right|+I\left(\tilde{f}-f^{*}\right)\leq M^{*}/2$ which implies that $\left|\hat{\beta }-\beta^{*}\right|+I\left(\hat{f}-f^{*}\right)\leq M^{*}$ and hence we can redo the above arguments and replace $\tilde{f}$ by $\hat{f}$.

**Case II.** If instead

$$2\lambda \sum_{j\in S_{*}}{\left\|f_{j}^{*}-{\tilde{f}}_{j}\right\|}_{n}+\lambda \left|\tilde{\beta }-\beta^{*}\right|\geq \rho M^{*}+ℰ\left(f^{*}\right)+2\lambda^{2}\sum_{j\in S_{*}}P_{st}\left(f_{j}^{*}\right),$$

then we have

(F.5)
$$ℰ\left(\tilde{f}\right)+\lambda \left\{\left|\tilde{\beta }-\beta^{*}\right|+I\left(\tilde{f}-f^{*}\right)\right\}\leq 4\lambda \sum_{j\in S_{*}}{\left\|f_{j}^{*}-{\tilde{f}}_{j}\right\|}_{n}+2\lambda \left|\tilde{\beta }-\beta^{*}\right|.$$

This is equivalent to

$$ℰ\left(\tilde{f}\right)+\lambda \left\{\sum_{j\in S_{*}^{c}}{\left\|{\tilde{f}}_{j}-f_{j}^{*}\right\|}_{n}+\lambda \sum_{j=1}^{p}P_{st}\left({\tilde{f}}_{j}-f_{j}^{*}\right)\right\}\leq 3\lambda \sum_{j\in S_{*}}{\left\|f_{j}^{*}-{\tilde{f}}_{j}\right\|}_{n}+\lambda \left|\tilde{\beta }-\beta^{*}\right|,$$

which means we have that

$$\sum_{j\in S_{*}^{c}}{\left\|{\tilde{f}}_{j}-f_{j}^{*}\right\|}_{n}+\lambda \sum_{j=1}^{p}P_{st}\left({\tilde{f}}_{j}-f_{j}^{*}\right)\leq 3\sum_{j\in S_{*}}{\left\|f_{j}^{*}-{\tilde{f}}_{j}\right\|}_{n}+\left|\tilde{\beta }-\beta^{*}\right|,$$

and hence by the compatibility condition (F.5) reduces to

$$ℰ\left(\tilde{f}\right)+\lambda \left\{\left|\tilde{\beta }-\beta^{*}\right|+I\left(\tilde{f}-f^{*}\right)\right\}\leq 4\lambda \left\|\tilde{f}-f^{*}\right\|\sqrt{s_{*}}/\phi \left(S_{*}\right).$$

Since $\tilde{f}$ and $f^{*}$ are in ${ℱ}_{\text{local}}^{0}$, we invoke the inequality uv≤H(v)+G(u) to obtain

$$\frac{4\lambda \sqrt{s_{*}}\left\|\tilde{f}-f^{*}\right\|}{\phi \left(S_{*}\right)}\leq \frac{8\lambda \sqrt{s_{*}}}{\phi \left(S_{*}\right)}\left[\frac{\left\|\tilde{f}-f^{0}\right\|}{2}+\frac{\left\|f^{*}-f^{0}\right\|}{2}\right]\;\leq H\left(\frac{8\lambda \sqrt{s_{*}}}{\phi \left(S_{*}\right)}\right)+G\left(\frac{\left\|\tilde{f}-f^{0}\right\|}{2}+\frac{\left\|f^{*}-f^{0}\right\|}{2}\right).$$

By the convexity of G and the margin condition we obtain

$$\frac{4\lambda \sqrt{s_{*}}\left\|\tilde{f}-f^{*}\right\|}{\phi \left(S_{*}\right)}\leq H\left(\frac{8\lambda \sqrt{s_{*}}}{\phi \left(S_{*}\right)}\right)+\frac{ℰ\left(\tilde{f}\right)}{2}+\frac{ℰ\left(f^{*}\right)}{2}.$$

Hence we have

$$\frac{ℰ\left(\tilde{f}\right)}{2}+\lambda \left\{\left|\tilde{\beta }-\beta^{*}\right|+I\left(\tilde{f}-f^{*}\right)\right\}\leq H\left(\frac{8\lambda \sqrt{s_{*}}}{\phi \left(S_{*}\right)}\right)+\frac{ℰ\left(f^{*}\right)}{2}\leq \rho M^{*}\leq \lambda M^{*}/8,$$

which implies that $\left|\tilde{\beta }-\beta^{*}\right|+I\left(\tilde{f}-f^{*}\right)\leq M^{*}/2$ which in turn implies $\left|\hat{\beta }-\beta^{*}\right|+I\left(\hat{f}-f^{*}\right)\leq M^{*}$ and hence we can redo the above arguments and replace $\tilde{f}$ by $\hat{f}$.

Thus we have shown that

(F.6)
$$ℰ(\hat{f})+\lambda \left\{\left|\hat{\beta }-\beta^{*}\right|+I\left(\hat{f}-f^{*}\right)\right\}\leq 4\rho M^{*}.$$

■

## Appendix G. The Set 𝒯

Theorems 7 and 12 show inequalities holding over the set 𝒯. In this section we will show that 𝒯 occurs with high probability. This will be shown for the two different types of entropy bounds considered in Section 4. We consider the special case of loss functions linear in $Y_{i}$ as in Corollaries 8 and 13 and bound the term $\nu_{n}(f)-\nu_{n}(f^{*})$ in the following theorem.

**Theorem G.1** *Let* $x_{i}\in {\mathbb{R}}^{p}$ *and* $Y_{i}\in \mathbb{R}$ *denote the fixed covariates and response*, *respectively*, *for* i=1,…,n. *Assume that for any function* f *the loss* ℓ(·) *is such that*

$$-\ell \left(f\right)=-\ell \left(f,x_{i},Y_{i}\right)=aY_{i}f\left(x_{i}\right)+b\left(f\left(x_{i}\right)\right),$$

*for some* a∈ℝ/{0} *and function* b:ℝ→ℝ. *Further assume that* $Y_{i}-EY_{i}=Y_{i}-\mu_{i}$ *are uniformly sub-Gaussian:*

(G.1)
$$\underset{i=1,\ldots ,n}{\text{max}}K^{2}\left(Ee^{{\left(Y_{i}-\mu_{i}\right)}^{2}/K^{2}}-1\right)\leq \sigma_{0}^{2},$$

*then with probability at-least* $1-2\text{exp}\left[-n\rho^{2}C_{1}\right]-C\text{exp}\left[-n\rho^{2}C_{2}\right]$ *the following inequality holds*

(G.2)
$$\nu_{n}\left(f\right)-\nu_{n}\left(f^{*}\right)\leq \rho \left[\left|\beta -\beta^{*}\right|+\sum_{j=1}^{p}{\left\|f_{j}-f_{j}^{*}\right\|}_{n}+\lambda P_{st}\left(f_{j}-f_{j}^{*}\right)\right],$$

*for variables* ρ, λ *and positive constants* C, $C_{1}$, $C_{2}$ *which we specify in the following 3 cases*.

***Case 1*.** *If* ℱ *has a logarithmic entropy bound*, *then the inequality* (G.2) *holds with* $\rho =\kappa \text{ max}\left(\sqrt{\frac{T_{n}}{n}},\sqrt{\frac{\text{log}\;p}{n}}\right)$ *and* λ=1 *for constants* $\kappa =\kappa (a,K,\sigma_{0},A_{0})$, $C_{1}=C_{1}(K,\sigma_{0})$, $C=C(K,\sigma_{0})$ *and* $C_{2}=C_{2}(C,\kappa )$.

***Case 2*.** *If* ℱ *has a polynomial entropy bound with smoothness*, *then the inequality* (G.2) *holds with* $\rho =\kappa \text{ max}\;\left(n^{-\frac{1}{2+\alpha }},\sqrt{\frac{\text{log}\;p}{n}}\right)$ *for constants* $\kappa =\kappa (a,K,\sigma_{0},A_{0},\alpha )$, $C_{1}=C_{1}(K,\sigma_{0})$, $C=C(K,\sigma_{0})$ *and* $C_{2}=C_{2}(C,\kappa )$. *The parameter satisfies* λ≍ρ *and* λ≥8ρ.

In light of the above theorem, for the case of Theorem 7 where

$$Z_{M^{*}}=\underset{I\left(f-f^{*}\right)\leq M^{*}}{\text{sup}}\left|\nu_{n}\left(f\right)-\nu_{n}\left(f^{*}\right)\right|,$$

and β, $\beta^{*}\in ℛ$ where ℛ is uniformly bounded by R then we have with probability at-least $1-2\text{ exp}[-n\rho^{2}C_{1}]-C\text{ exp}[-n\rho^{2}C_{2}]$,

$$Z_{M^{*}}\leq \rho \left(M^{*}+2R\right).$$

In the case of Theorem 15 where

$$Z_{M^{*}}=\underset{\left|\beta -\beta^{*}\right|+I\left(f-f^{*}\right)\leq M^{*}}{\text{sup}}\left|\nu_{n}(f)-\nu_{n}\left(f^{*}\right)\right|,$$

we have with probability at-least $1-2\text{ exp}[-n\rho^{2}C_{1}]-C\text{ exp}[-n\rho^{2}C_{2}]$,

$$Z_{M^{*}}\leq \rho M^{*}$$

To prove Theorem G.1, we use a few technical lemmas from van de Geer (2000) namely Lemma 8.2 and Corollary 8.3; for the sake of completeness we state these lemmas in Appendix I.

**Proof** [Proof of Theorem G.1] We begin by noting that for any arbitrary function we have

$$\nu_{n}\left(f\right)=\left(P_{n}-P\right)\left(-\ell \left(f\right)\right)=\frac{1}{n}\sum_{i=1}^{n}\left[aY_{i}f\left(x_{i}\right)+b\left(f\left(x_{i}\right)\right)\right]-E\left[\frac{1}{n}\sum_{i=1}^{n}aY_{i}f\left(x_{i}\right)+b\left(f\left(x_{i}\right)\right)\right].$$

Since we assume the covariates $x_{1},\ldots ,x_{n}$ are fixed we obtain

$$\nu_{n}\left(f\right)=\frac{1}{n}\sum_{i=1}^{n}a\left(Y_{i}-\mu_{i}\right)f\left(x_{i}\right)\equiv a{\langle Y-\mu ,f\rangle }_{n},$$

where $\mu_{i}=EY_{i}$. Thus for additive functions f and $f^{*}$ we obtain

$$\nu_{n}\left(f\right)-\nu_{n}\left(f^{*}\right)=a{\langle Y-\mu ,f-f^{*}\rangle }_{n}=\frac{1}{n}\sum_{i=1}^{n}a\left(Y_{i}-\mu_{i}\right)\left[\beta -\beta^{*}+\sum_{j=1}^{p}f_{j}\left(x_{ij}\right)-f_{j}^{*}\left(x_{ij}\right)\right]\;=a\left(\beta -\beta^{*}\right)\frac{1}{n}\sum_{i=1}^{n}\left(Y_{i}-\mu_{i}\right)+\sum_{j=1}^{p}\frac{1}{n}\sum_{i=1}^{n}a\left(Y_{i}-\mu_{i}\right)\left(f_{j}\left(x_{ij}\right)-f_{j}^{*}\left(x_{ij}\right)\right)\;=a\left(\beta -\beta^{*}\right)(\bar{Y}-\bar{\mu })+\sum_{j=1}^{p}a{\langle Y-\mu ,f_{j}-f_{j}^{*}\rangle }_{n}.$$

From now on we will assume, without loss of generality, that |a|=1 since this constant is absorbed into a constant κ which we define later.

To control the first term, $\left(\beta -\beta^{*}\right)(\bar{Y}-\bar{\mu })$, we simply apply Lemma I.1. For the second part, we consider 2 cases.

**Case 1: Logarithmic Entropy.** We first note that if the entropy bound holds, then the same bound holds (upto a constant) for the class

$$\left\{\frac{f_{j}-f_{j}^{*}}{{\left\|f_{j}-f_{j}^{*}\right\|}_{n}+\lambda P_{st}\left(f_{j}-f_{j}^{*}\right)}:f_{j}\in ℱ\right\},$$

for some $f_{j}^{*}\in ℱ$. for all j=1,…,p. Now we apply Lemma I.2 to the above class by first noting that R≤1 and then using the bound for Dudley’s integral

$$A_{0}^{1/2}T_{n}^{1/2}\int_{0}^{1}{\text{log}}^{1/2}\left(\frac{1}{u}+1\right)du\leq {\tilde{A}}_{0}T_{n}^{1/2},$$

we have for all δ that satisfy

$$\delta \geq 2C{\tilde{A}}_{0}\sqrt{\frac{T_{n}}{n}},$$

where the constant C depends only on K and $\sigma_{0}$, we have

$$P\left(\underset{f_{j}\in ℱ}{\text{sup}}\left|\frac{\frac{1}{n}\sum_{i=1}^{n}\left(Y_{i}-\mu_{i}\right)\left(f_{j}\left(x_{ij}\right)-f_{j}^{*}\left(x_{ij}\right)\right)}{{\left\|f_{j}-f_{j}^{*}\right\|}_{n}+\lambda P_{st}\left(f_{j}-f_{j}^{*}\right)}\right|\geq \delta \right)\leq C\text{exp}\left[-\frac{n\delta^{2}}{4C^{2}}\right].$$

We can now take $\delta =\rho \geq 2C{\tilde{A}}_{0}\text{max}\left\{\sqrt{\frac{T_{n}}{n}},\sqrt{\frac{\text{log}p}{n}}\right\}\geq 2C{\tilde{A}}_{0}\sqrt{\frac{T_{n}}{n}}$ which holds for all $\kappa \geq 2C{\tilde{A}}_{0}$. Applying the above result with a union bound gives us

$$P\left(\underset{j=1,\ldots ,p}{\text{max}}\underset{f_{j}\in ℱ}{\text{sup}}\frac{\left|{\langle Y-\mu ,f_{j}-f_{j}^{*}\rangle }_{n}\right|}{{\left\|f_{j}-f_{j}^{*}\right\|}_{n}+\lambda P_{st}\left(f_{j}-f_{j}^{*}\right)}\geq \rho \right)\;\leq pC\text{exp}\left[-\frac{n\rho^{2}}{4C^{2}}\right]=C\text{exp}\left[-\frac{n\rho^{2}}{4C^{2}}+\text{log}\;p\right]\;=C\text{exp}\left[-n\rho^{2}\left(\frac{1}{4C^{2}}-\frac{\text{log}\;p}{n\rho^{2}}\right)\right]\leq C\text{exp}\left[-n\rho^{2}C_{2}\right],$$

for a constant $C_{2}>0$ that depends on C and ${\tilde{A}}_{0}$. To see this, note that

$$\frac{1}{4C^{2}}-\frac{\text{log}\;p}{n\rho^{2}}=\frac{1}{4C^{2}}-\frac{1}{\kappa \text{max}\left\{\frac{T_{n}}{\text{log}\;p},1\right\}}\geq \frac{1}{4C^{2}}-\frac{1}{\kappa },$$

which is positive if $\kappa >4C^{2}$. Thus we can take the constant κ such that $\kappa >\text{max}\left\{4C^{2},2C{\tilde{A}}_{0}\right\}$. Hence κ depends on $C(K,\sigma_{0})$ and ${\tilde{A}}_{0}\left(A_{0}\right)$.

**Case 2: Polynomial Entropy with Smoothness.** Now we note that same entropy bound holds for the class

$$\tilde{ℱ}=\left\{\frac{f_{j}-f_{j}^{*}}{{\left\|f_{j}-f\right\|}_{jn}^{*}+\lambda P_{st}\left(f_{j}-f_{j}^{*}\right)}:f_{j}\in ℱ\right\},$$

and we can now apply Lemma I.2 by noting that

$$\int_{0}^{1}H^{1/2}\left(u,\tilde{ℱ},Q_{n}\right)du\leq {\tilde{A}}_{0}\lambda^{-\alpha /2},$$

for some constant ${\tilde{A}}_{0}={\tilde{A}}_{0}\left(A_{0}\right)$. For some $C=C(K,\sigma_{0})$ and all $\delta \geq 2C{\tilde{A}}_{0}\lambda^{-\alpha /2}n^{-1/2}$ we have

(G.3)
$$P\left(\underset{f_{j}\in ℱ}{\text{sup}}\frac{\left|{\langle Y-\mu ,f_{j}-f_{j}^{*}\rangle }_{n}\right|}{{\left\|f_{j}-f_{j}^{*}\right\|}_{n}+\lambda P_{st}\left(f_{j}-f_{j}^{*}\right)}\geq \delta \right)\leq C\text{exp}\left[-\frac{n\delta^{2}}{4C^{2}}\right].$$

Since λ≥ρ we note that $2C{\tilde{A}}_{0}\lambda^{-\alpha /2}n^{-1/2}\leq 2C{\tilde{A}}_{0}\rho^{-\alpha /2}n^{-1/2}$ and that

$$2C{\tilde{A}}_{0}\rho^{-\alpha /2}n^{-1/2}\leq \rho \Leftrightarrow \rho \geq {\left(2C{\tilde{A}}_{0}\right)}^{\frac{2}{2+\alpha }}n^{-\frac{1}{2+\alpha }}.$$

Which holds by definition since $\rho =\kappa \;\text{max}\;\left(\sqrt{\frac{\text{log}\;p}{n}},n^{-\frac{1}{2+\alpha }}\right)\geq \kappa n^{-\frac{1}{2+\alpha }}$ and κ is sufficiently large (any $\kappa \geq {\left(2C{\tilde{A}}_{0}\right)}^{\frac{2}{2+\alpha }}$ would suffice). Therefore, we can take δ=ρ in (G.3) along with a union bound to obtain

$$P\left(\underset{j=1,\ldots ,p}{\text{max}}\underset{f_{j}\in ℱ}{\text{sup}}\frac{\left|{\langle Y-\mu ,f_{j}-f_{j}^{*}\rangle }_{n}\right|}{{\left\|f_{j}-f_{j}^{*}\right\|}_{n}+\lambda P_{st}\left(f_{j}-f_{j}^{*}\right)}\geq \rho \right)\leq pC\text{exp}\left[-\frac{n\rho^{2}}{4C^{2}}\right]\;=C\text{exp}\left[-n\rho^{2}\left(\frac{1}{4C^{2}}-\frac{\text{log}\;p}{n\rho^{2}}\right)\right]\;\leq C\text{exp}\left[-n\rho^{2}C_{2}\right],$$

for some positive constant $C_{2}=C_{2}(C,{\tilde{A}}_{0})$ exactly as in Case 1. ■

## Appendix H. On the Margin and Compatibility Coniditions

In this appendix, we present a detailed discussion of the margin and compatibility conditions. These conditions are the main assumptions made for our theoretical results and in this appendix we discuss suitable conditions under which the conditions hold.

### H.1 Margin Condition

We now present a general framework for proving the quadratic margin condition for loss functions given by the negative log-likelihood of exponential family distributions. Specifically, for loss functions of the type

(H.1)
$$-\ell \left(f\right)=-\ell \left(y_{i};f\left(x_{i}\right)\right)=ay_{i}f\left(x_{i}\right)+b\left(f\left(x_{i}\right)\right).$$

The excess risk is

$$ℰ(f)=P\left\{\ell \left(f^{0}\right)-\ell \left(f\right)\right\}\;=\frac{1}{n}\sum_{i=1}^{n}\left\{-aE\left(y_{i}\right)\right\}\left\{f^{0}\left(x_{i}\right)-f\left(x_{i}\right)\right\}-\left[b\left\{f^{0}\left(x_{i}\right)\right\}-b\left\{f\left(x_{i}\right)\right\}\right]\;=\frac{1}{n}\sum_{i=1}^{n}\left[b^{\prime }\left\{f^{0}\left(x_{i}\right)\right\}\right]\left\{f^{0}\left(x_{i}\right)-f\left(x_{i}\right)\right\}-\left[b\left\{f^{0}\left(x_{i}\right)\right\}-b\left\{f\left(x_{i}\right)\right\}\right],$$

where the last equality holds because the expectation of the score function is zero. Now by Taylor’s theorem for b(·) and the mean value theorem for $b^{\prime }(\cdot )$, the above simplifies to

$$ℰ(f)=n^{-1}\sum_{i=1}^{n}b^{\prime \prime }\left(\zeta_{1i}\right){\left\{f^{0}\left(x_{i}\right)-f_{i}\left(x_{i}\right)\right\}}^{2}-\frac{b^{\prime \prime }\left(\zeta_{2i}\right)}{2}{\left\{f^{0}\left(x_{i}\right)-f\left(x_{i}\right)\right\}}^{2}\;\geq \underset{C}{\underset{︸}{\underset{i}{\text{min}}\left\{b^{\prime \prime }\left(\zeta_{1i}\right)-\frac{b^{\prime \prime }\left(\zeta_{2i}\right)}{2}\right\}}}{\left\|f^{0}-f\right\|}_{n}^{2},$$

where $\zeta_{1i},\zeta_{2i}$ lie between $f(x_{i})$, $f^{0}(x_{i})$. Thus for loss functions of the type (H.1), we only need to find a constant C in a neighborhood of $f^{0}$. As an example, consider the least squares loss where

$$b\left(\theta \right)=\theta^{2}/2,\;\;\;b^{\prime \prime }\left(\theta \right)=1\Rightarrow C=1/2.$$

Similarly, for logistic regression we have

$$b\left(\theta \right)=\text{log}\left\{\text{exp}\left(\theta \right)+1\right\},\;\;\;b^{\prime \prime }\left(\theta \right)=\frac{\text{exp}\left(\theta \right)}{1+\text{exp}\left(\theta \right)}.$$

Now on the set ${ℱ}_{\text{local}}^{0}=\left\{f:{\left\|f-f^{0}\right\|}_{\infty }\leq \eta \right\}$, we need only look at the interval $\left(f^{0}(x_{i})-\eta ,f^{0}(x_{i})+\eta \right)$. As a simple case, say $f^{0}(x_{i})=0$, then

$$b^{\prime \prime }\left(\zeta_{1i}\right)-\frac{b^{\prime \prime }\left(\zeta_{2i}\right)}{2}\geq \frac{\text{exp}\left\{\eta \right\}}{{\left[1+\text{exp}\left\{\eta \right\}\right]}^{2}}-\frac{\text{exp}\left\{0\right\}}{2{\left[1+\text{exp}\left\{0\right\}\right]}^{2}}>0,$$

for $\eta <\text{log}\left(3+2\sqrt{2}\right)$. While tedious, for any $f^{0}(x_{i})$, we can find an η such that $b^{\prime \prime }(\zeta_{1i})-b^{\prime \prime }(\zeta_{2i})/2>0$ for all $\zeta_{1i}$, $\zeta_{2i}$. The above examples indicate that the margin condition is satisfied in most of the relevant problems.

### H.2 Compatibility Condition

We now show that under suitable conditions, the theoretical compatibility condition implies the compatibility condition. Recall first our notation, $∥f{∥}^{2}=\int [f(x){]}^{2}dQ(x)$ where Q is the probability measure of our observed data. We denote by $Q_{n}$, the empirical measure associated with Q, and ${\left\|f\right\|}_{n}^{2}=n^{-1}\sum_{i=1}^{n}f^{2}\left(x_{i}\right)$.

**Lemma H.1** *On the set*

(H.2)
$$𝒮=\left\{\underset{f\in ℱ}{\text{sup}}\frac{\left|{\left\|f\right\|}_{n}-\left\|f\right\|\right|}{\sum_{j=1}^{p}\left\|f_{j}\right\|+\lambda P\left(f_{j}\right)}\leq \eta \right\},$$

*with* η∈(0,1/5), *suppose we have*

(H.3)
$$c_{1}=\eta \left\{\frac{2\left(2+\eta \right)}{1-\eta }+\frac{3\left(1+\eta \right)}{1-5\eta }\right\}\frac{\sqrt{\left|S\right|}}{\tilde{\phi }\left(S\right)}<1,$$

*if the theoretical compatibility condition holds with* η *as in* (H.2). *Then*, *the empirical compatibility condition holds with constant*

$${\left\{\phi \left(S\right)\right\}}^{-1}=\left\{\left(1+\eta \right)+\frac{3\eta \left(1+\eta \right)}{1-5\eta }\right\}\frac{1}{\left(1-c_{1}\right)\tilde{\phi }\left(S\right)}.$$

**Remark H.2** *The event* 𝒮, *ensures that our empirical norm is relatively close to the theoretical norm; existing literature shows that* 𝒮 *holds with high probability under suitable entropy conditions for small* η (*see for example*, Section 5 *of* Tan and Zhang, 2019). *The condition* (H.3), *simply forces our index set* S *to not be too large; thus*, *highlighting the role of sparsity in high dimensions*.

To prove Lemma H.1, we will state and prove a sequence of smaller lemmas, the proof will then follow immediately.

For ease of exposition/reference, we restate our theoretical and (empirical) compatibility conditions. For simplicity, we do not include the intercept term; explicitly including an intercept term does not change the proof.

**Definition H.3 (Empirical Compatibility Condition)** *The compatibility condition is said to hold for an index set* S⊂{1,2,…,p} *with* s=|S|, *and with compatibility constant* ϕ(S)>0, *if for all* λ>0 *and all functions* f *of the form* $f\left(x\right)=\sum_{j=1}^{p}f_{j}\left(x_{j}\right)$ *that satisfy*

$$\begin{array}{ll} \sum_{j\in S^{c}}{\left\|f_{j}\right\|}_{n}+\sum_{j=1}^{p}\lambda P_{st}\left(f_{j}\right)\leq 3\sum_{j\in S}{\left\|f_{j}\right\|}_{n}, & \left(\text{Empirical}-\text{A}\right) \end{array}$$

*it holds that*

$$\begin{array}{ll} \sum_{j\in S}{\left\|f_{j}\right\|}_{n}\leq {\left\|f\right\|}_{n}\sqrt{s}/\phi \left(S\right). & \left(\text{Empirical}-\text{B}\right) \end{array}$$

**Definition H.4 (Theoretical Compatibility Condition)** *The theoretical compatibility condition is said to hold for an index set* S⊂{1,2,…,p} *with* s=|S|, *and for a compatibility constant* $\tilde{\phi }(S)>0$, *if for some* η∈(0,1/5), *all* λ>0, *and all functions of the form* $f(x)=\sum_{j=1}^{p}f_{j}\left(x_{j}\right)$ *that satisfy*

$$\begin{array}{ll} \sum_{j\in S^{c}}\left\|f_{j}\right\|+\frac{1-5\eta }{1-\eta }\sum_{j=1}^{p}\lambda P_{st}\left(f_{j}\right)\leq \frac{3\left(1+\eta \right)}{1-\eta }\sum_{j\in S}\left\|f_{j}\right\|, & \left(\text{Theoretical}-\text{A}\right) \end{array}$$

*it holds that*

$$\begin{array}{ll} \sum_{j\in S}\left\|f_{j}\right\|\leq \left\|f\right\|\sqrt{s}/\tilde{\phi }\left(S\right). & \left(\text{Theoretical}-\text{B}\right) \end{array}$$

**Proof** [Proof of Lemma H.1] We will show that on the set 𝒮 given in (H.2), we have the following sequence of implications.

$$\left(\text{Empirical}-\text{A})\overset{1}{\Rightarrow }(\text{Theoretical}-\text{A})\overset{2}{\Rightarrow }(\text{Theoretical}-\text{B})\overset{3}{\Rightarrow }(\text{Empirical}-\text{B}\right)$$

1. The result of Lemma H.5.
2. By assuming the theoretical compatility condition.
3. Follows immediately from Lemmas H.6 and H.7.

■

**Lemma H.5** *On the set* 𝒮, (Empirical-A) ⇒ (Theoretical-A).

**Proof** We have

$$\left|{\left\|f_{j}\right\|}_{n}-\left\|f_{j}\right\|\right|\leq \eta \left(\left\|f_{j}\right\|+\lambda P\left(f_{j}\right)\right),$$

which is equivalent to

(H.4)
$$(1-\eta )\left\|f_{j}\right\|-\eta \lambda P\left(f_{j}\right)\leq {\left\|f_{j}\right\|}_{n}\leq \left(1+\eta \right)\left\|f_{j}\right\|+\eta \lambda P\left(f_{j}\right).$$

Thus we have the following:

$$\sum_{j\in S^{c}}\|f_{j}\|+\sum_{j=1}^{n}\lambda P\left(f_{j}\right)\;\leq \frac{1}{1-\eta }\sum_{j\in S^{c}}{\left\|f_{j}\right\|}_{n}+\frac{\eta }{1-\eta }\sum_{j\in S^{c}}\lambda P\left(f_{j}\right)+\sum_{j=1}^{p}\lambda P\left(f_{j}\right)\text{by}\;(\text{H}.4)\;=\frac{1}{1-\eta }\sum_{j\in S^{c}}{\left\|f_{j}\right\|}_{n}+\frac{1}{1-\eta }\sum_{j\in S^{c}}\lambda P\left(f_{j}\right)+\sum_{j\in S}\lambda P\left(f_{j}\right)\;=\frac{1}{1-\eta }\left\{\sum_{j\in S^{c}}{\left\|f_{j}\right\|}_{n}+\sum_{j=1}^{p}\lambda P\left(f_{j}\right)\right\}+\frac{\eta }{1-\eta }\sum_{j\in S}\lambda P\left(f_{j}\right)\;\leq \frac{3}{1-\eta }\sum_{j\in S}{\left\|f_{j}\right\|}_{n}+\frac{\eta }{1-\eta }\sum_{j\in S}\lambda P\left(f_{j}\right)\text{by}\;(\text{Empirical}-\text{A})\;\leq \frac{3}{1-\eta }\times \left\{\sum_{j\in S}\left(1+\eta \right)\left\|f_{j}\right\|+\eta \lambda P\left(f_{j}\right)\right\}+\frac{\eta }{1-\eta }\sum_{j\in S}\lambda P\left(f_{j}\right)\text{by}\;(\text{H}.4)\;=\frac{3\left(1+\eta \right)}{1-\eta }\sum_{j\in S}\left\|f_{j}\right\|+\frac{4\eta }{1-\eta }\sum_{j\in S}\lambda P\left(f_{j}\right).$$

Taking the smoothness norm term to the left hand side completes the proof. ■

**Lemma H.6** *On the set* 𝒮, *assuming the theoretical compatibility condition*, *if*

$$c_{1}=\eta \left\{\frac{2\left(2+\eta \right)}{1-\eta }+\frac{3\left(1+\eta \right)}{1-5\eta }\right\}\frac{\sqrt{s}}{\tilde{\phi }\left(S\right)}<1,$$

*then*

$$\left\|f\right\|\leq \frac{1}{1-c_{1}}{\left\|f\right\|}_{n}.$$

**Proof** On the set 𝒮 we have

$$\begin{array}{ll} \left\|f\right\|\leq {\left\|f\right\|}_{n}+\eta \left\{\sum_{j=1}^{p}\left\|f_{j}\right\|+\lambda P\left(f_{j}\right)\right\} & \text{by}\;\text{definition} \end{array}$$

For terms inside the brackets we have:

$$\sum_{j=1}^{p}\left\|f_{j}\right\|=\sum_{j\in S}\left\|f_{j}\right\|+\sum_{j\in S^{c}}\left\|f_{j}\right\|\;\leq \sum_{j\in S}\left\|f_{j}\right\|+\frac{3\left(1+\eta \right)}{1-\eta }\sum_{j\in S}\left\|f_{j}\right\|\text{by}\;\left(\text{Theoretical}-\text{A}\right)\;=\frac{2\left(2+\eta \right)}{1-\eta }\sum_{j\in S}\left\|f_{j}\right\|,$$

and

$$\begin{array}{ll} \sum_{j=1}^{p}\lambda P\left(f_{j}\right)\leq \frac{3\left(1+\eta \right)}{1-5\eta }\sum_{j\in S}\left\|f_{j}\right\| & \text{by}\;\left(\text{Theoretical}-\text{A}\right). \end{array}$$

So now we have:

$$\begin{array}{ll} \left\|f\right\|\leq {\left\|f\right\|}_{n}+\eta \left\{\frac{2\left(2+\eta \right)}{1-\eta }\sum_{j\in S}\|f_{j}\|+\frac{3\left(1+\eta \right)}{1-5\eta }\sum_{j\in S}\left\|f_{j}\right\|\right\} & \\ \leq {\left\|f\right\|}_{n}+\eta \left\{\frac{2\left(2+\eta \right)}{1-\eta }+\frac{3\left(1+\eta \right)}{1-5\eta }\right\}\frac{\sqrt{s}\left\|f\right\|}{\tilde{\phi }\left(S\right)}. & \text{by}\;(\text{Theoretical}-\text{B)} \end{array}$$

By definition of $c_{1}$ we get

$$\left(1-c_{1}\right)\left\|f\right\|\leq {\left\|f\right\|}_{n},$$

which completes the proof. ■

**Lemma H.7** *Under the conditions of Lemma H.6*, (Theoretical-B) ⇒ (Empirical-B) *with*

$${\left\{\phi \left(S\right)\right\}}^{-1}=\left\{\left(1+\eta \right)+\frac{3\eta \left(1+\eta \right)}{1-5\eta }\right\}\frac{1}{\left(1-c_{1}\right)\tilde{\phi }\left(S\right)}.$$

**Proof** We have that

$$\begin{array}{ll} \sum_{j\in S}{\left\|f_{j}\right\|}_{n}\leq \left(1+\eta \right)\sum_{j\in S}\left\|f_{j}\right\|+\eta \sum_{j\in S}\lambda P\left(f_{j}\right) & \text{by}\;(\text{H}.4)\\ \leq \left(1+\eta \right)\sum_{j\in S}\left\|f_{j}\right\|+\frac{3\eta \left(1+\eta \right)}{1-5\eta }\sum_{j\in S}\left\|f_{j}\right\| & \text{by}\;(\text{Theoretical}-\text{A})\\ \leq \left\{\left(1+\eta \right)+\frac{3\eta \left(1+\eta \right)}{1-5\eta }\right\}\frac{\sqrt{s}\left\|f\right\|}{\tilde{\phi }(S)} & \text{by}\;(\text{Theoretical}-\text{B})\\ \leq \left\{\left(1+\eta \right)+\frac{3\eta \left(1+\eta \right)}{1-5\eta }\right\}\frac{\sqrt{s}}{\tilde{\phi }(S)}\frac{{\left\|f\right\|}_{n}}{1-c_{1}}, & \text{by}\;\text{Lemma}\;\text{H}.6\\ \equiv {\left\|f\right\|}_{n}\sqrt{s}/\phi \left(S\right). & \end{array}$$

■

## Appendix I. Some Results from van de Geer (2000)

**Lemma I.1 (Lemma 8.2 of** van de Geer (2000)) *Suppose that* $Y_{1}-\mu_{1},\ldots ,Y_{n}-\mu_{n}$ *are mean zero sub-Gaussian random variables*, *satisfying* (G.1). *Then for all* $\gamma \in {\mathbb{R}}^{n}$ *and* ρ>0,

$$P\left(\left|\sum_{i=1}^{n}\left(Y_{i}-\mu_{i}\right)\gamma_{i}\right|\geq \rho \right)\leq 2\text{exp}\left[-\frac{\rho^{2}}{8\left(K^{2}+\sigma_{0}^{2}\right)\sum_{i=1}^{n}\gamma_{i}^{2}}\right],$$

*in particular if* $\gamma_{i}=1/n$ *then we have*

$$P\left(\left|\frac{1}{n}\sum_{i=1}^{n}Y_{i}-\mu_{i}\right|\geq \rho \right)\leq 2\text{exp}\left[-\frac{n\rho^{2}}{8\left(K^{2}+\sigma_{0}^{2}\right)}\right].$$

**Lemma I.2 (Corollary 8.3 of** van de Geer (2000)) *Suppose that* ${\text{sup}}_{f_{j}\in ℱ}{\left\|f_{j}\right\|}_{n}\leq R$ *for a univariate function class* ℱ *and that* $Y_{1}-\mu_{1},\ldots ,Y_{n}-\mu_{n}$ *are mean zero sub-Gaussian random variables*, *satisfying* (G.1). *Then for some constant* $C=C(K,\sigma_{0})$, *and for all* δ>0 *satisfying*

$$\sqrt{n}\delta \geq 2C\left(\int_{0}^{R}H^{1/2}\left(u,ℱ,Q_{n}\right)du\vee R\right),$$

we have

$$P\left(\underset{f_{j}\in ℱ}{\text{sup}}\left|\frac{1}{n}\sum_{i=1}^{n}\left(Y_{i}-\mu_{i}\right)f_{j}\left(x_{ij}\right)\right|\geq \delta \right)\leq C\text{exp}\left[-\frac{n\delta^{2}}{4C^{2}R^{2}}\right].$$
